# Development and Characterization of Novel Selective, Non-Basic Dopamine D_2_ Receptor Antagonists for the Treatment of Schizophrenia

**DOI:** 10.3390/molecules28104211

**Published:** 2023-05-20

**Authors:** Piotr Stępnicki, Sylwia Wośko, Agata Bartyzel, Agata Zięba, Damian Bartuzi, Klaudia Szałaj, Tomasz M. Wróbel, Emilia Fornal, Jens Carlsson, Ewa Kędzierska, Ewa Poleszak, Marián Castro, Agnieszka A. Kaczor

**Affiliations:** 1 Department of Synthesis and Chemical Technology of Pharmaceutical Substances with Computer Modeling Laboratory, Faculty of Pharmacy, Medical University of Lublin, 4A Chodźki St., PL-20093 Lublin, Poland; agata.zieba@umlub.pl (A.Z.); damian.bartuzi@umlub.pl (D.B.); tomasz.wrobel@umlub.pl (T.M.W.); 2 Laboratory of Preclinical Testing, Chair and Department of Applied and Social Pharmacy, Faculty of Pharmacy, Medical University of Lublin, Chodźki 1, PL-20093 Lublin, Poland; sylwia.wosko@umlub.pl (S.W.); ewa.poleszak@umlub.pl (E.P.); 3 Department of General and Coordination Chemistry and Crystallography, Institute of Chemical Sciences, Faculty of Chemistry, Maria Curie-Skłodowska University in Lublin, Maria Curie-Skłodowska Sq. 2, PL-20031 Lublin, Poland; agata.bartyzel@mail.umcs.pl; 4 Science for Life Laboratory, Department of Cell and Molecular Biology, Uppsala University, 75124 Uppsala, Sweden; jens.carlsson@icm.uu.se; 5 Department of Bioanalytics, Chair of Dietetics and Bioanalytics, Faculty of Biomedicine, Medical University of Lublin, Jaczewskiego 8b St., PL-20090 Lublin, Poland; klaudia.szalaj@umlub.pl (K.S.); emilia.fornal@umlub.pl (E.F.); 6 Department of Pharmacology and Pharmacodynamics, Faculty of Pharmacy, Medical University of Lublin, 4A Chodźki St., PL-20093 Lublin, Poland; ewa.kedzierska@umlub.pl; 7 Center for Research in Molecular Medicine and Chronic Diseases (CiMUS), Universidade de Santiago de Compostela, Avda de Barcelona, E-15782 Santiago de Compostela, Spain; marian.castro@usc.es; 8 Instituto de Investigación Sanitaria de Santiago de Compostela (IDIS), Travesía da Choupana s/n, E-15706 Santiago de Compostela, Spain; 9 School of Pharmacy, University of Eastern Finland, P.O. Box 1627, FI-70211 Kuopio, Finland

**Keywords:** antipsychotic, dopamine D_2_ receptor, schizophrenia, SAR studies, GPCRs, lead optimization

## Abstract

The dopamine D_2_ receptor, which belongs to the family of G protein-coupled receptors (GPCR), is an important and well-validated drug target in the field of medicinal chemistry due to its wide distribution, particularly in the central nervous system, and involvement in the pathomechanism of many disorders thereof. Schizophrenia is one of the most frequent diseases associated with disorders in dopaminergic neurotransmission, and in which the D_2_ receptor is the main target for the drugs used. In this work, we aimed at discovering new selective D_2_ receptor antagonists with potential antipsychotic activity. Twenty-three compounds were synthesized, based on the scaffold represented by the D2AAK2 compound, which was discovered by our group. This compound is an interesting example of a D_2_ receptor ligand because of its non-classical binding to this target. Radioligand binding assays and SAR analysis indicated structural modifications of D2AAK2 that are possible to maintain its activity. These findings were further rationalized using molecular modeling. Three active derivatives were identified as D_2_ receptor antagonists in cAMP signaling assays, and the selected most active compound **17** was subjected to X-ray studies to investigate its stable conformation in the solid state. Finally, effects of **17** assessed in animal models confirmed its antipsychotic activity in vivo.

## 1. Introduction

G protein-coupled receptors, also termed seven transmembrane (7TM) receptors, are commonly considered the largest group of molecular targets for medications on the market [1,2]. According to estimates, around one-third of approved drugs act through interacting with this group of proteins [3], which may be explained by the multitude of physiological processes that are regulated by these receptors and their impact on signaling pathways associated with the pathology of many medical conditions, such as metabolic and endocrine disorders, infections, cancer, and central nervous system disorders [4].

Dopamine receptors constitute one of the most important subfamilies within Class A of the G protein-coupled receptors (GPCRs), playing a vital role in regulating several key processes in the human body such as mood, learning, attention, and motor skills [5,6]. In particular, the dopamine D_2_ receptor is assigned an extremely important role in the pathomechanism of diseases of the central nervous system. One of the most common, and at the same time one of the most difficult to treat among the diseases related to disorders in dopaminergic transmission, is schizophrenia. Its clinical manifestations include positive symptoms (delusions, hallucinations), negative symptoms (anhedonia, social withdrawal), and cognitive symptoms associated with memory deficits and information-processing disorders [7]. Based on their mechanism of action, antipsychotics are classified into so-called classical drugs (first generation), which are non-selective D_2_ receptor antagonists, and atypical drugs (second and third generation), which usually display a weaker effect on the D_2_ receptor, but possess a higher affinity for additional molecular targets, especially serotonin 5-HT_2A_ and 5-HT_1A_ receptors. However, the D_2_ receptor still remains the main target for antipsychotic drugs and is crucial for the treatment of the main symptoms of schizophrenia, i.e., positive ones [8,9].

**Figure 1:**
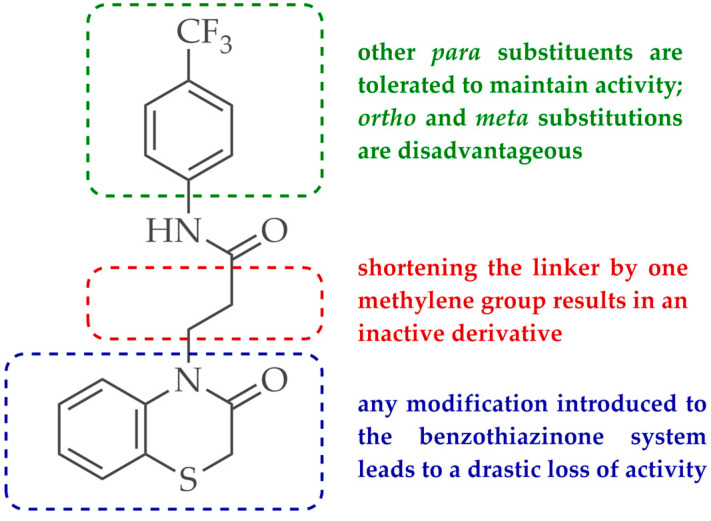
Chemical structure of D2AAK2 and the summary of SAR studies for its derivatives.

In previous studies, Kaczor et al. conducted a virtual screening campaign aimed at identifying ligands of the dopamine D_2_ receptor for potential use as lead structures for the development of new antipsychotic drugs, followed by a pharmacological evaluation of the activity of selected entries in relation to D_2_, as well as other dopamine and serotonin receptors [10]. Among the identified hits, the structure of compound **2** (hereinafter referred to as D2AAK2), shown in Figure 1, is of particular novelty due to the lack of a basic nitrogen atom, which constitutes a key element in the classical pharmacophore model for orthosteric ligands of aminergic GPCRs. When protonated at physiological pH, this nitrogen is able to form an ionic bond with aspartic acid Asp 3.32 at the orthosteric binding site of the receptor, which is usually considered the main interaction stabilizing the ligand-receptor complex. Despite the lack of a protonatable nitrogen atom, D2AAK2, as demonstrated in radioligand binding assays, shows an affinity for the D_2_ receptor (*K*_i_ = 321 nM). Moreover, it also exhibits high selectivity over other closely related targets, as it is not active at the tested dopamine D_1_ and D_3_, and serotonin 5-HT_1A_ and 5-HT_2A_ receptors.

To date, very few D_2_ receptor ligands are known that lack a basic nitrogen atom and thus do not follow the classical pharmacophore model for binding to this target. As an example, Free et al. developed a series of selective dopamine D_2_ receptor antagonists, most of which lack a protonatable amino nitrogen group [11,12]. In particular, the compound ML321 was selected and evaluated in detail, which confirmed its high selectivity for the D_2_ receptor, both in vitro and in vivo. In addition, molecular modeling has shown that the proton of the amide group present in the molecule can form a hydrogen bond with the carboxyl group in the side chain of Asp 3.32 [11].

The aim of our work presented here was to investigate which structural features of D2AAK2 are crucial for ensuring its affinity for the dopamine D_2_ receptor and how the introduction of various structural modifications affects this activity. For this purpose, a series of D2AAK2 derivatives were designed and synthesized. Their activity at the D_2_ receptor was then assessed in radioligand binding assays. A summary of the introduced structural modifications and their effect on the activity of the obtained derivatives is shown in Figure 1. As it turned out, only minor changes involving the *para* substituent in the D2AAK2 phenyl ring are tolerated to maintain activity at the D_2_ receptor. Both substitutions in a different position of the phenyl and modifications of the remaining fragments of the molecule are highly unfavorable and lead to loss of activity. This indicates that the *para*-substituted aniline and benzothiazinone systems are crucial for the binding of D2AAK2 and its corresponding analogs to the D_2_ receptor. Subsequently, selected compounds that showed activity in radioligand binding assays were evaluated in the functional assays of cAMP signaling, which revealed their antagonist properties at the D_2_ receptor. Molecular modeling was utilized to investigate the binding mode and interaction of the studied compounds in the complexes with the receptor. In addition, for compound **17**, which showed the highest, comparable to D2AAK2, an affinity for the D_2_ receptor out of the obtained series, crystallographic studies were performed, which enabled to study of its detailed conformation in a single crystal. This derivative has also been evaluated in a panel of behavioral tests to investigate its activity as a potential drug candidate for the treatment of schizophrenia.

## 2. Results and Discussion

### 2.1. Chemistry

**Scheme 1:**
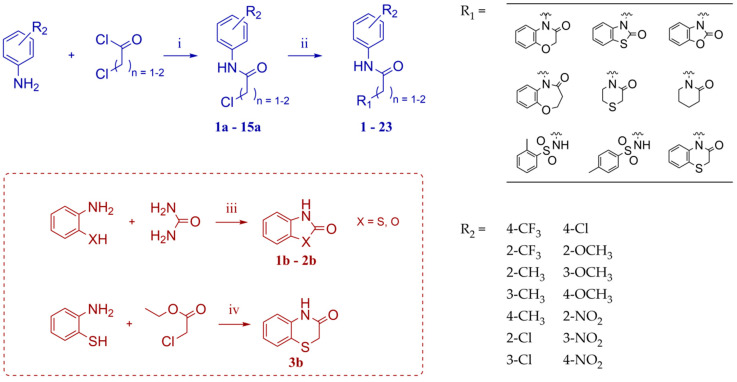
Synthetic route for obtaining compounds **1**–**23**. Reagents and conditions: (i) acetone, reflux, 1 h; (ii) K_2_CO_3_, ACN, reflux, overnight (for **1**–**3**, **5**, **7**–**23**); K_2_CO_3_, DMF, rt., overnight (for **4**); KOH, DMSO, rt., overnight (for **6**); (iii) water, reflux, 36 h; (iv) KOH, ethanol 96°, reflux, 3 h.

Compounds **1**–**23** were obtained according to the synthetic route shown in Scheme 1, which consisted of two steps. Initially, suitable anilines underwent acylation with chloroacetyl chloride or 3-chloropropionyl chloride. Following this step, the resulting intermediates (**1a**–**15a**) were employed for the alkylation of the respective lactam or sulfonamide, ultimately yielding the final compounds. Some of these lactams (**1b**–**3b**), were not commercially available, thus they were synthesized according to the synthetic pathways shown in Scheme 1. Benzo[*d*]thiazol-2(3*H*)-one and benzo[*d*]oxazol-2(3*H*)-one were obtained following the previously reported green method for synthesis of 2-substituted benzoxazoles and benzothiazoles [13]. 2*H*-benzo[*b*][1,4]thiazin-3(4*H*)-one, in turn, was synthesized by adopting the known method, in the reaction of 2-aminothiophenol with ethyl chloroacetate [14].

### 2.2. Affinity of Compounds at D_2_ Receptor and Structure-Activity Relationship

**Figure 2:**
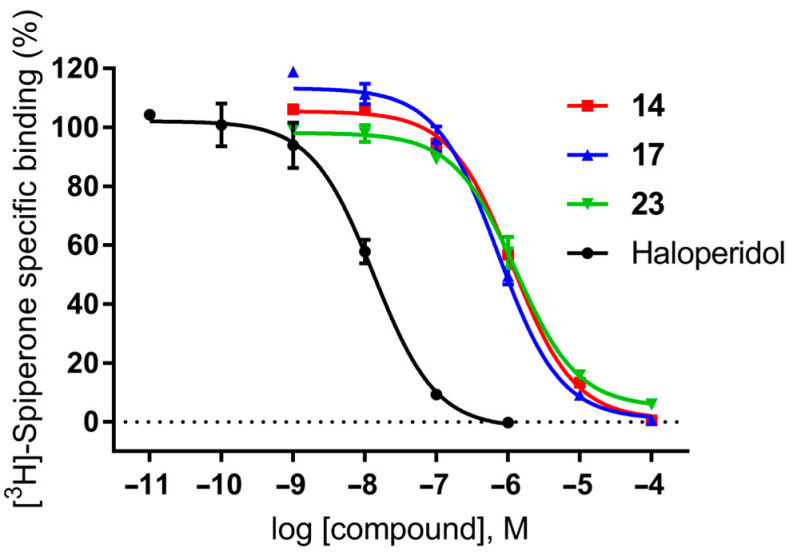
Competition radioligand binding curves for compounds **14**, **17** and **23**, and haloperidol as a standard competitor ligand at the human-cloned D_2_ receptor. The graph shows the data (mean ± SEM) of a representative experiment out of 2–3 independent experiments performed in duplicate.

In order to assess the affinity of the synthesized compounds for dopamine D_2_ receptor, the whole series was subjected to evaluation in competition radioligand binding assays. Experiments were carried out in membranes isolated from a CHO-K1 cell line stably overexpressing the cloned human D_2S_ receptor. In an initial screening, the compounds were tested at a single concentration of 10^−5^ M, and concentration-response curves were carried out for those that showed more than 50% inhibition of the specific radioligand binding. *K*_i_ values were determined for the compounds that achieved full displacement of the specific radioligand binding in a concentration-dependent manner at the concentrations assayed (concentration ranges from 10^−10^ or 10^−9^ M to 10^−5^ or 10^−4^ M). Affinities of the compounds, expressed as *K*_i_ [nM] or as % of inhibition of specific radioligand binding at the concentration of 10^−5^ M (% inh.), are shown in Table 1. Competition radioligand binding curves for the most active compounds **14**, **1**, **7** and **23**, and for haloperidol, which was used as the reference standard, are shown in Figure 2.

Analyzing the structure-activity relationship among the obtained series of compounds, it appears evident that only the replacement of the substituent in the *para* position of the phenyl ring is a tolerable structural modification that allows maintaining the activity towards the D_2_ receptor. The structure-activity relationship (SAR) for this series appears to be relatively flat. A chloro substituent is preferred, resulting in an affinity comparable to that of the trifluoromethyl group in the D2AAK2 structure [10]. In turn, both methyl and nitro substituents lead to an approximately two-fold lower affinity for the studied receptor. The least preferred substitution at this position appears to be a methoxy group. Changing the position of the substituents from *para* to *meta* or *ortho* results in a loss of activity, however, it seems that of the two positions, the *meta* position is preferable. The lack of substitution on the phenyl ring also results in a similar loss of activity as in the case of installing substituents at the *meta* position, indicating the importance of the *para* substitution in maintaining the activity of the compound. The introduction of any modifications to the benzothiazinone system, as well as the shortening of the linker connecting the distal fragments of the molecule by one methylene group, results in inactive derivatives, which again underlines the fact that only minor structural modifications of the D2AAK2 compound are acceptable to maintain an affinity for the D_2_ receptor.

### 2.3. Determination of the Affinity Profile of Selected Compounds for Additional GPCRs of Interest

Compounds **14**, **17**, and **23**, which showed good to moderate affinity at the D_2_ receptor, were subjected to further evaluation in order to determine their selectivity over additional GPCRs, namely dopaminergic D_1_ and D_3_, and serotonergic 5-HT_1A_, 5-HT_2A_ and 5-HT_7_ receptors stably overexpressed in cell lines. None of the compounds showed affinity at the tested receptors, proving their high selectivity towards the D_2_ receptor (Table 2). This is in line with the receptor profile of the compound D2AAK2 [10], indicating that changing the substituent in the phenyl ring while retaining its position maintains selective activity at the D_2_ receptor.

### 2.4. Efficacy of Selected Compounds at D_2_ Receptor

The efficacy of compounds **14**, **17**, and **23** as antagonists of the D_2_ receptor was evaluated in functional assays of cAMP signaling in CHO-K1 cells stably overexpressing the human cloned dopamine D_2S_ receptor, with the use of haloperidol as a reference antagonist of D_2_ receptor. The estimated potencies of the studied compounds extracted from the assays are expressed as p*K*_b_ and *K*_b_ (nM) values (Table 3). According to the obtained results compounds **14**, **17**, and **23** display antagonism at the dopamine D_2_ receptor, with potencies in good agreement with their rank of affinities toward this target. Based on the radioligand binding assays and functional assays results, compound **17** was chosen for further evaluation as the most active compound from the series.

### 2.5. Molecular Modeling

The original D2AAK2 structure was discovered using virtual screening, with the D_2_ receptor homology model as the target. Since then, various X-ray and cryo-EM structures of the D_2_ receptor have been reported, revealing significant variability in the conformations of extracellular loops ECL1 and ECL2 that impact the binding pocket’s shape [15,16,17]. Based on these findings, we investigated the docking poses of D2AAK2 and its derivatives at different conformations of the inactive-state D_2_ receptor, represented by the structures deposited to date. Our goal was to identify which of these conformations are most favorable for D2AAK2 binding and find the most plausible ligand binding mode, allowing us to rationalize further attempts at structure optimization.

The earliest report on inactive D_2_ receptor structure was provided by Wang et al. in 2018 [15]. Their 2.87 Å X-ray structure (PDB ID: 6CM4) differs significantly from other D_2_-family receptor structures in the arrangement of ECL1, with a very distinct, exposed conformation of the conserved Trp100 residue. The authors of the contribution mention the apparent interactions of the extracellular loops with the engineered T4 lysozyme in the crystal lattice. While in their interpretation those interactions were too weak to induce the rearrangement, such conformation is not seen in any other D_2_-family receptor resolved structure, including the more recent structure by Im et al. [17] (PDB ID: 7DFP) of the D_2_ receptor binding spiperone, which, similarly to risperidone, reaches the deep secondary binding pocket in the area of the conserved Trp 6.48. In the latter structure, in contrast to risperidone from the earlier report, ECL1 is arranged in a way analogical to other available dopamine receptor structures [18,19], and accommodates its outward-protruding aromatic moiety in a cleft created by Phe 3.28 rotation. However, the 7DFP structure contains an antibody bound to the extracellular side of the receptor, with some of the antibody residues reaching down to the area neighboring the orthosteric pocket, leading to a question of whether the conformation of the ligand is affected by this interaction. In the work of Fan et al. [16] the authors did not use any antibody, and the construct was similar to the 6CM4 structure with the T4L fragment used to replace intracellular loop ICL3 (PDB ID: 6LUQ). In that structure, while ECL2 engages in crystal-packing interactions and remains flipped outward compared to other D_2_-family receptor structures similar to 6CM4, ECL1 assumes more conventional conformation, but compared to 7DFP structure, the Phe 3.28 side chain is flipped inward. Therefore, likely due to a different experimental setup, all the mentioned structures differ in the manner significantly affecting the extracellular side of the binding pocket.

**Figure 3:**
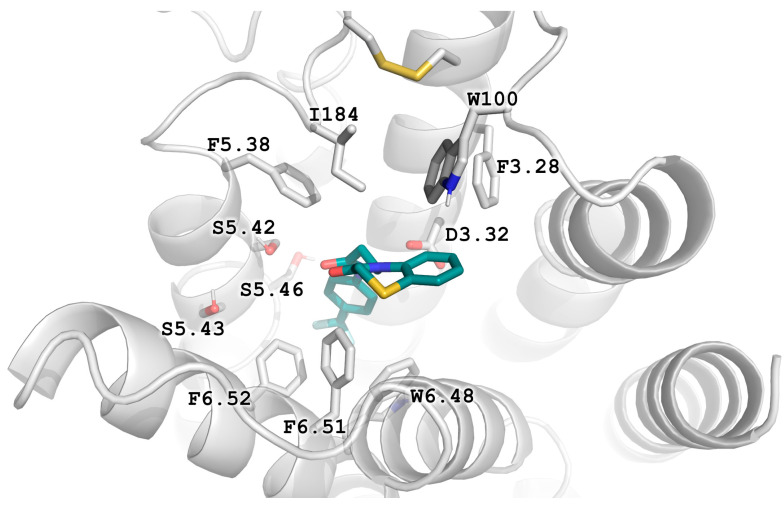
Prevailing binding mode of D2AAK2 and its derivatives as seen in the results of docking to the 6CM4 structure. Apparent T-stacking of the ligand’s condensed ring and the conserved Trp100 residue seem to stabilize this orientation.

**Figure 4:**
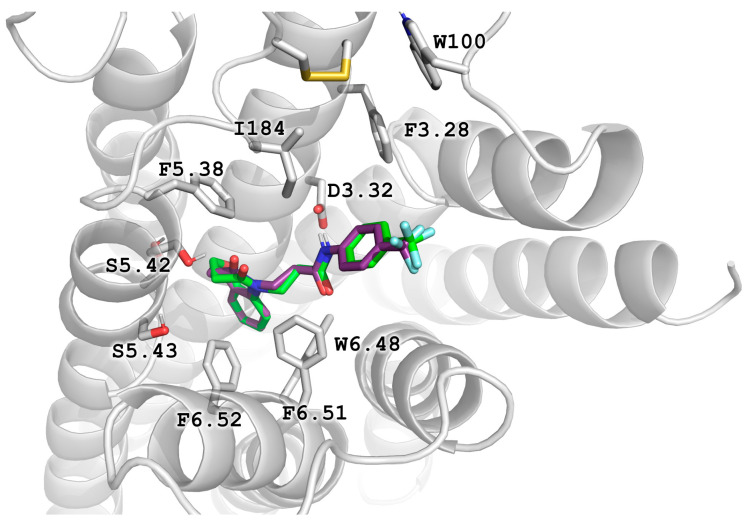
Binding poses of the active compounds D2AAK2 (violet) and its derivative **4** (green), as docked to the structure 6LUQ with top scoring values.

**Figure 5:**
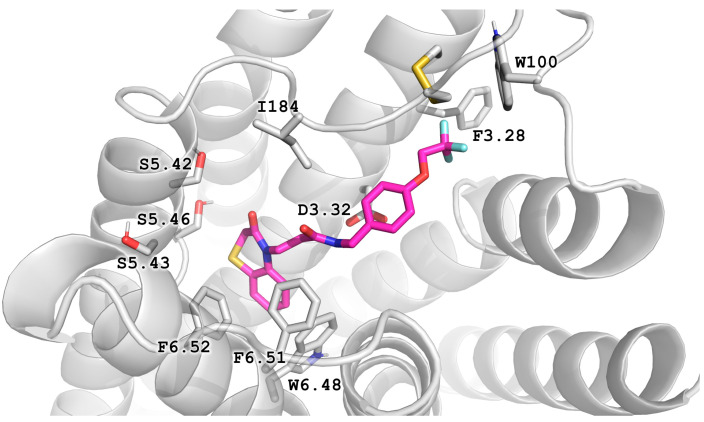
The docking pose of the compound Z238240766 (compound **22** in Kaczor et al., 2016 [10]) in the 7DFP structure indicates the possibility of filling the cleft created by Phe 3.28 rotation with -CF_3_ substituent.

Among the results of D2AAK2 docking to all three structures, poses at the 6CM4 structure were the least consistent regarding the ligand orientation. Moreover, only in this structure does a large fraction of the top-scored results places the ligand’s condensed ring moiety at the extracellular side of the binding pocket. This can be attributed to the unusual location of the Trp100 side chain, engaging in favorable aromatic interactions with the ligand (Figure 3). This is contrary to conformations obtained during the virtual screening campaign, as well as in the docking poses at 7DFP and 6LUQ structures, where the benzothiazinone moiety and its analogs are usually situated deeper and near the TM5. Such orientation is assumed in a vast majority of docking poses in these structures. Furthermore, docking to the 6LUQ structure accurately identifies D2AAK2 as the best-fitting compound from the set, and ranks another active molecule, **4**, in second place (Figure 4). Ligand positions in the docking to 7DFP structure are generally very similar to those seen in both docking to 6LUQ and the virtual screen, except for the apparent preference of the trifluoromethyl moiety to occupy the cleft created after Phe 3.28 rotation, not present in other structures (Figure 5). Such consistency of docking poses, together with satisfactory ranking of ligands by scoring function suggests that 7DFP and 6LUQ X-ray structures are more likely to represent protein conformations corresponding to the one responsible for binding D2AAK2 and its derivatives than the 6CM4 structure.

Results of the molecular docking were further analyzed to better understand ligand-protein interactions and rationalize the observed structure-activity relationship. The visual inspection of the docking poses well explains the complete loss of activity in derivatives **5** and **6**, as they are deprived of the aromatic ring supposed to bind in the region of TM5, responsible for accommodating aromatic moieties of both endogenous and exogenous ligands in aminergic receptors. This observation provides further support to the assumption of 7DFP and 6LUQ structures better representing the D2AAK2-binding conformation, as in the results of docking to the 6CM4 structure the TM5-neighbouring part of the binding pocket is in most cases occupied by the phenyl moiety (Figure 3). The lack of activity of the derivative **7** can be therefore explained by the methyl substituent disrupting T-stacking interaction with Trp 6.48 or being forced against Ser 5.42 and Ser 5.43 residues, as well as by locating sulfonamide in a hydrophobic pocket composed of Trp 6.48, Phe 6.51 and Phe 6.52. The latter would also apply to the derivative **8**.

Dependence of ligand activity on the size of the lactam ring and substituting sulfur with oxygen seems to result from a combination of three factors: protein-ligand shape complementarity, hydrogen bonding with Ser 5.42 and Ser 5.43, and aromatic stacking with Trp 6.48. Replacing the benzothiazine ring with a more strained 2-benzothiazole in derivative **2** results in a more rigid and flat condensed ring system, apparently less complementary to the shape of the binding pocket, potentially causing the carbonyl oxygen position to be less favorable for establishing hydrogen bonds with TM5 serine residues. The same mechanism would apply to compound **3**. The seven-membered ring of **4**, in turn, seems to fit the binding pocket, while still decreasing activity compared to the initial compound by 10-fold, probably due to disturbance of aromatic stacking with Phe 6.51 and Phe 6.52. The case of compound **1** is particularly interesting, as the replacement of sulfur with oxygen alone cancels the activity of the compound completely, which is even more surprising considering the neighborhood of TM5 serines that could be suspected to form hydrogen bonds with oxygen. This would suggest that hydrogen bonds, if present, are formed rather with the carbonyl oxygen of the lactam ring. In that case, a possible explanation of the activity loss would be the decrease in spatial complementarity due to the alteration of angles in the ring, which could also move the carbonyl oxygen into an orientation unfavorable for hydrogen bonding.

While the docking position of the benzothiazinone moiety is very consistent in 7DFP and 6LUQ structures and corresponds to the observed SAR, it is more difficult to draw conclusions and make predictions about the effects of possible modifications of the phenyl moiety, as it is docked differently in both target structures. Especially surprising is the decrease in the activity of the Enamine compound Z238240766 (compound **22** in Kaczor et al. [10]) derivative reported in our previous work [10], where the trifluoroethoxy substituent seems to perfectly fit the cleft under ECL2 of the 7DFP structure. This, together with the inactivity of the 4-methoxy derivative **20** shows that neither extending the *para* substituent nor introducing a methylene linker between amide and phenyl groups is desired, and the phenyl moiety may prefer a different arrangement. On the other hand, the moiety seems to fit a well-defined pocket as seen in 7DFP dockings rather than protrude to the solvent as in 6LUQ-derived poses, since moving the trifluoromethyl or methyl substituent to *meta* or *ortho* position results in complete loss of activity, while substitutions in *para* position seem to be more acceptable. Moreover, the area near the disulfide bridge under ECL2 would seem to be a plausible candidate for trifluoromethyl moiety binding, as such substituents are known to form favorable interactions with sulfur [20].

**Figure 6:**
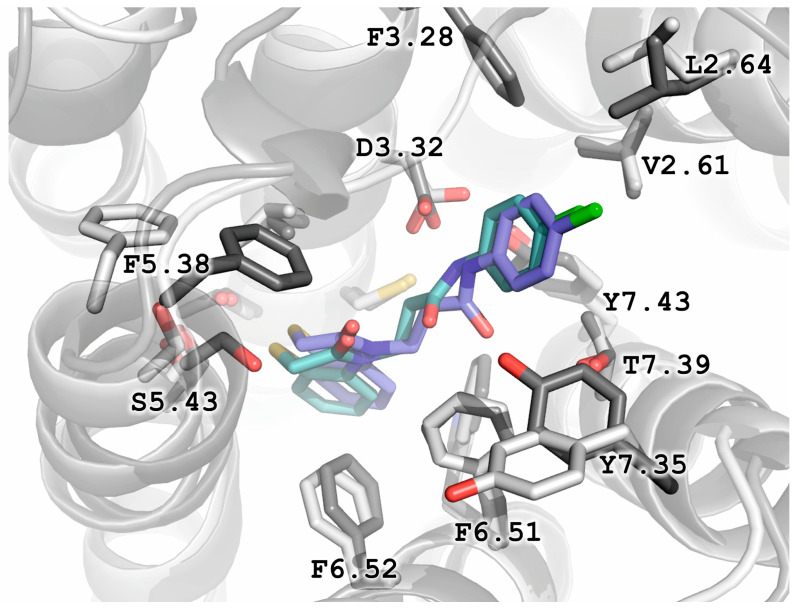
Docking poses of compound **17** in X-ray models of dopamine D_2_ receptor (PDB IDs: 6LUQ, sidechains in dark gray; and 7DFP, sidechains in white).

Figure 6 shows the docking poses of the most potent compound **17** in the binding pocket of dopamine D_2_ receptor (X-ray models, PDB IDs: 6LUQ and 7DFP; 6CM4 omitted for clarity). Notably, the binding pose of compound **17** overlaps in all three considered X-ray models of dopamine D2 receptor, in contrast to the poses of the virtual hit D2AAK2. It should be stressed that the interaction of the amide group of compound 17 with the conserved Asp3.32 is key in the case of this compound, irrespectively of the used X-ray receptor model. Interestingly, the positions of Phe3.28 and Tyr7.35 side chains in 6LUQ PDB structure (but not in 7DFP structure), directed to the receptor interior and involved in interactions with compound **17** tighten the pocket around the 4-chlorophenyl moiety, which would pose a steric hindrance to meta-substituted derivatives and thus explain their lack of potency.

### 2.6. X-ray Studies

**Figure 7:**
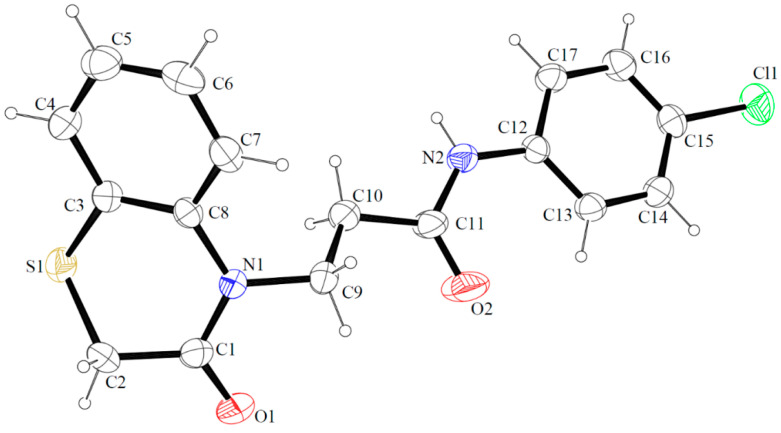
The molecular structures of compound **17** showing atomic labeling. Displacement ellipsoids are drawn at the 30% probability level.

The molecular structure of compound **17** with the atomic numbering scheme is shown in Figure 7 and selected bond distances and angles are reported in Table S1 (See Supplementary Materials). Single crystal X-ray analysis reveals that the compound crystallizes in the centrosymmetric monoclinic system (*P*2_1_/*c*) with one molecule in the asymmetric unit. The structure consists of one fused-ring system (2*H*-1,4-benzothiazin-3(4*H*)-one fragment) linked by propanamide moiety with a 4-chlorophenyl unit. Interatomic distances and bond angles are in the expected ranges [21] and are comparable to those observed in the related 3,4-dihydro-2*H*-1,4-benzothiazin-3-one [22,23,24,25,26,27,28,29] and *N*-(4-chlorophenyl)propanamide [30,31,32,33,34,35] derivatives. The fused ring system is nonplanar (r.m.s. deviation = 0.2515 Å for all non-H atoms with a maximum deviation of −0.605(3) Å for C(2)), the dihedral angle between the planes formed by non-hydrogen atoms of the phenyl (C3/C4/C5/C5/C7/C8) and thiazine (C1/C2/S1/C3/C8/N1) rings is 15.8(2)°. The mean plane formed by non-hydrogen atoms of the fused ring system (C1/C2/S1/C3/C4/C5/C5/C7/C8/N1) is inclined to the plane of phenyl (C12/C13/C14/C15/C16/C17) ring by 54.1(1)°. The thiazine ring is in a screw-boat conformation, as indicated by the following puckering parameters: Q = 0.645(3)°, θ = 65.5(4)°, φ = 32.3(4)° [36,37,38]. As can be seen from Figure S1 in the crystal, molecules of **17** are linked by strong N2-H2⋯O2^i^ hydrogen-bonding interactions (symmetry code i = x, y−1, z) forming the chain structures along [010] direction with graph-set motif C(9) (Figure S2) [39]. The hydrogen bonding parameters are given in Table 4.

**Figure 8:**
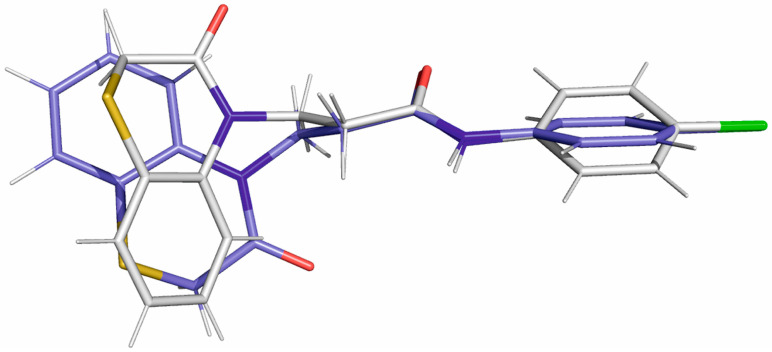
The superposition of docked (white) and X-ray (blue) conformations of compound **17** is based on the superposition of the amide group.

Figure 8 shows the superposition of the docked (white) and X-ray conformation (blue) of compound **17** based on the superposition of the amide group. Importantly, while in the docking pose the conjugated ring system is rotated, both conformations assume similar shape and occupy similar volume. However, due to the differences in the environment and the occurring interactions (intermolecular vs. with amino acids), it would be difficult to draw relevant conclusions by comparing the conformation of compound **17** in the solid state with its arrangement in the receptor.

### 2.7. Behavioral Studies

#### 2.7.1. Effect of Compound **17** on Locomotor Activity in Mice

The effect of compound **17** (25, 50, and 75 mg/kg) on the spontaneous locomotor activity in mice is shown in Table 5. Statistical analysis of the results demonstrated that **17** at doses of 25, 50, and 75 mg/kg had no statistically significant effects on the animal’s locomotor activity versus the control group after 10 min (one-way ANOVA: F (3, 36) = 0.7972, *p* = 0.5035) and after 30 min (one-way ANOVA: F (3, 36) = 0.6207, *p* = 0.6062).

#### 2.7.2. Motor Coordination Evaluated by Chimney Test

**Figure 9:**
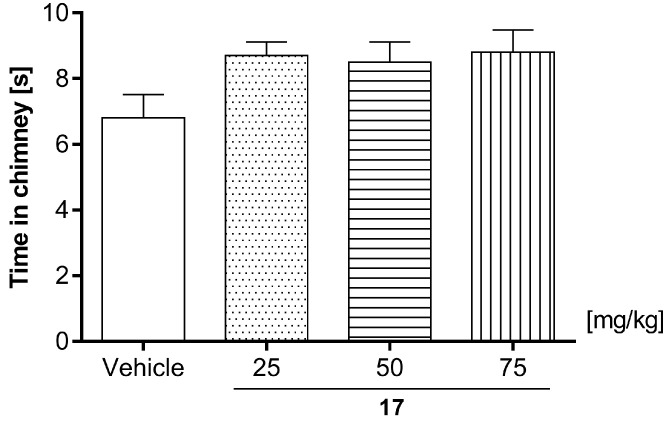
Effect of compound **17** (25, 50, and 75 mg/kg) on the motor coordination evaluated by chimney test in mice. **17** (25, 50, and 75 mg/kg) and vehicle were injected (i.p) 60 min before testing. The values represent mean + SEM (*n* = 10 animals per group); (one-way ANOVA followed by Dunnett’s post hoc test).

Compound **17** administered at doses of 25, 50, and 75 mg/kg did not change the behavior of mice in the chimney (one-way ANOVA: F (3, 36) = 2.316; *p* = 0.0921). The mice were leaving the chimney within the allotted time of 30 s. The effect of **17** (25, 50, and 75 mg/kg) on the motor coordination evaluated by chimney test in mice is shown in Figure 9.

#### 2.7.3. Effects of Compound **17** in the Rotarod Test

**Figure 10:**
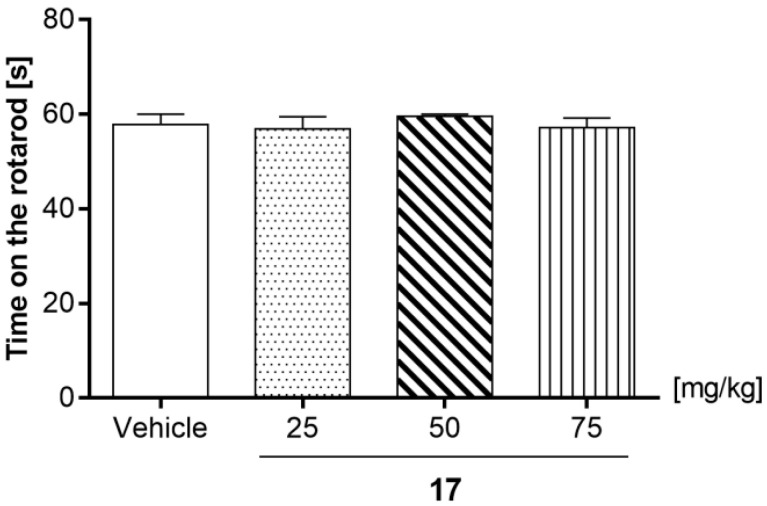
Effect of compound **17** (25, 50, and 75 mg/kg) on the motor coordination evaluated by rotarod test in mice. **17** (25, 50, and 75 mg/kg) and vehicle were injected (i.p) 60 min before testing. The values represent mean + SEM (*n* = 10 animals per group). The data are presented as the mean ± SEM. The results were considered statistically significant if *p* < 0.05 (one-way ANOVA followed by Dunnett’s post hoc test).

An acute administration of compound **17** at the tested doses (25, 50, and 75 mg/kg) did not influence mice behavior in the rotarod test [one-way ANOVA: F (3, 36) = 0.3809; *p* = 0.7673]. The effect of **17** (25, 50, and 75 mg/kg) on the motor coordination evaluated by the rotarod test in mice is shown in Figure 10.

Locomotor activity and motor coordination assays are generally accepted as basic tests for investigations of the central activity of new agents [40]. Compound **17** administered at the doses 25, 50, and 75 mg/kg did not impair the coordination of animals, as evaluated in the chimney and rotarod tests (Figure 9 and Figure 10) or alter the spontaneous activity (Table 5), therefore further behavioral tests were conducted for all above doses of **17**.

#### 2.7.4. Spontaneous Locomotor Activity and Amphetamine-Induced Hyperactivity

**Figure 11:**
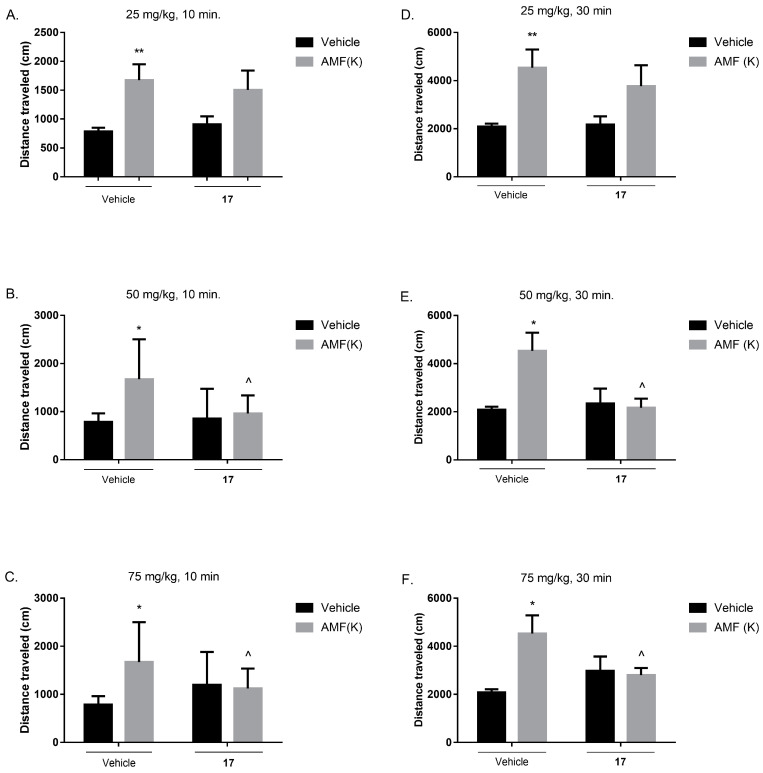
Acute effect of **17** on the amphetamine-induced hyperactivity in mice after 10 min, administered at the dose of 25 mg/kg (**A**), 50 mg/kg **(B**) and 75 mg/kg (**C**), and after 30 min at the dose of 25 mg/kg (**D**), 50 mg/kg (**E**) and 75 mg/kg (**F**). Compound **17** (25, 50, and 75 mg/kg) and vehicle were injected i.p. Amphetamine (3 mg/kg) (s.c) was administered 30 min after injection of **17** (25, 50, and 75 mg/kg). The values represent mean ± SEM (*n* = 8–10 animals per group). The data are presented as the mean ± SEM. ** *p* ˂ 0.01, * *p* ˂ 0.05 versus control group; ^ *p* ˂ 0.05 versus amphetamine (3 mg/kg) group (two-way ANOVA followed by Bonferroni’s post hoc test).

The effect of **17** co-administration with amphetamine after 10 min (10 min from cpd injection) in mice is shown in Figure 11A–C and after 30 min in Figure 11D–F.

Two-way ANOVA revealed that 10 min after compound **17** (25, 50, and 75 mg/kg, i.p.) administration followed by amphetamine co-injection (3 mg/kg, s.c., 30 min after compound administration) there is a statistically significant treatment effect (amphetamine administration) in each case, respectively: [F (1, 33) = 9.420, *p* = 0.0043; Figure 11A; [F (1, 33) = 7.197, *p* = 0.0113; Figure 11B] and [F (1, 32) = 4.181, *p* = 0.0492; Figure 11C] and statistically significant interaction effect between treatment (amphetamine administration) and pretreatment (compound administration) only after higher doses, respectively for 50 mg/kg [F (1, 33) = 4.407, *p* = 0.0435; Figure 11B] and 75 mg/kg [F (1, 32) = 5.876, *p* = 0.0212; Figure 11C], whereas no statistically significant pretreatment effect was observed [F (1, 33) = 0.3645, *p* = 0.5502; Figure 11A]. Moreover, Bonferroni’s post hoc test revealed that amphetamine treatment increased mice locomotor activity when compared to the control group (** *p* < 0.01; Figure 11A, * *p* < 0.05; Figure 11B,C), and that amphetamine-induced hyperactivity was decreased by compound **17** administration at the dose of 50 and 75 mg/kg (^ *p* < 0.05).

Two-way ANOVA revealed that 30 min after compound **17** (25, 50 and 75 mg/kg, i.p.) administration followed by amphetamine co-injection (3 mg/kg, s.c., 30 min after compound administration) there is a statistically significant pretreatment effect (amphetamine administration) in each case, respectively: [F (1, 31) = 9.997, *p* = 0.0035; Figure 11D]; [F (1, 31) = 4.498, *p* = 0.0420; Figure 11E] and [F (1, 31) = 4.886, *p* = 0.0346; Figure 11F] and statistically significant interaction effect between treatment (amphetamine administration) and pretreatment (compound administration) only after higher doses, respectively for 50 mg/kg [F (1, 31) = 5.998, *p* = 0.0202; Figure 11E] and 75 mg/kg [F (1, 31) = 6.526, *p* = 0.0158; Figure 11F], whereas no statistically significant pretreatment effect was observed [F (1, 31) = 0.4432, *p* = 0.5105; Figure 11D]. Moreover, Bonferroni’s post hoc test revealed that amphetamine treatment increased mice locomotor activity when compared to the control group (** *p* < 0.01; Figure 11D, * *p* < 0.05; Figure 11E,F) and that amphetamine-induced hyperactivity was decreased by compound **17** administration at the dose of 50 and 75 mg/kg (^ *p* < 0.05).

Amphetamine-induced hyperactivity test is an animal model reflecting the positive symptoms of schizophrenia commonly employed for preclinical studies [41]. The model is based on the manipulation of the dopaminergic system, and it may primarily respond to drugs that affect this neurotransmitter system. Many neuroleptics acting as dopaminergic antagonists reverse this effect [42]. It is noteworthy that amphetamine-induced hyperlocomotion is also sensitive to other classes of drugs [43,44]. Behavioral studies confirmed the antipsychotic activity of **17** in relation to the positive symptoms of schizophrenia, which was expected based on in vitro test results. The compound decreased the hyperactivity induced by the administration of amphetamine in mice at studied doses of 50 and 75 mg/kg, whereas it was inactive at the lower dose of 25 mg/kg. These results should be further supplemented with an assessment of the effect of compound **17** on the negative and cognitive symptoms of schizophrenia.

#### 2.7.5. The Effect of Acute Administration of Compound **17** on Anxiety Responses in Mice

**Figure 12:**
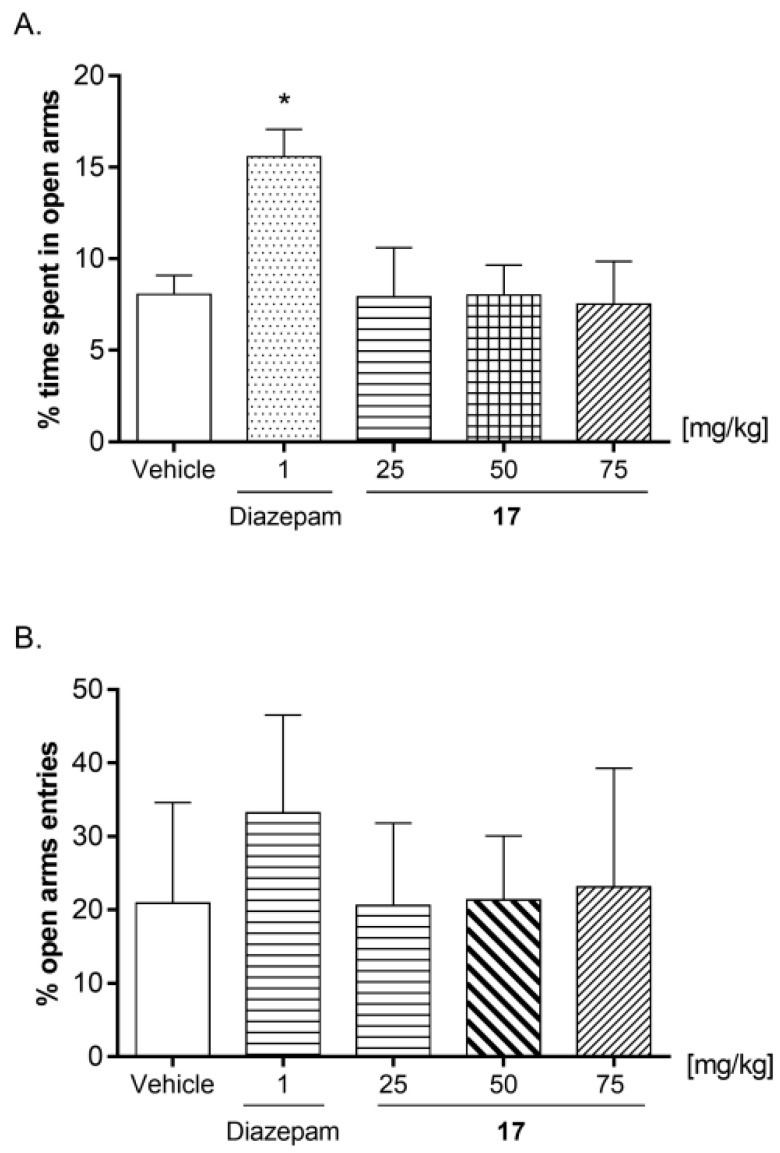
The effects of compound **17** (25, 50, and 75 mg/kg) on the percentage of time spent in open arms (**A**) and percentage of entries into open arms (**B**) in the EPM procedure. A single dose of **17** (25, 50, and 75 mg/kg) and vehicle were injected i.p. Diazepam at the dose of 1 mg/kg was administered s.c. All substances were injected 60 min before the test. The values represent mean ± SEM (*n* = 7–8 mice per group). * *p* < 0.05 versus vehicle (post-hoc Bonferroni’s test).

The influence of compound **17** (25, 50, and 75 mg/kg) on the anxiety-like responses was determined in the EPM test (Figure 12). Diazepam at a dose of 1 mg/kg was administrated as a reference drug with documented anxiolytic activity. Compound **17** administered at a dose of 25, 50, and 75 mg/kg did not change the percentage of the time spent (Figure 12A; ANOVA: F (4, 30) = 3.419; *p* = 0.0204) or entries into open arms of maze (Figure 12B; ANOVA: F (4, 31) = 1.318; *p* = 0.2851). Diazepam (1 mg/kg) induced a statistically significant increase in the time animals spent in the open arms of the maze (Figure 12A; * *p* < 0.05 versus vehicle; post-hoc Bonferroni’s test). The obtained results indicated that compound **17** does not influence anxiety-like behavior in mice.

#### 2.7.6. Compound **17** Dose-Effect Relationship in the FST

**Figure 13:**
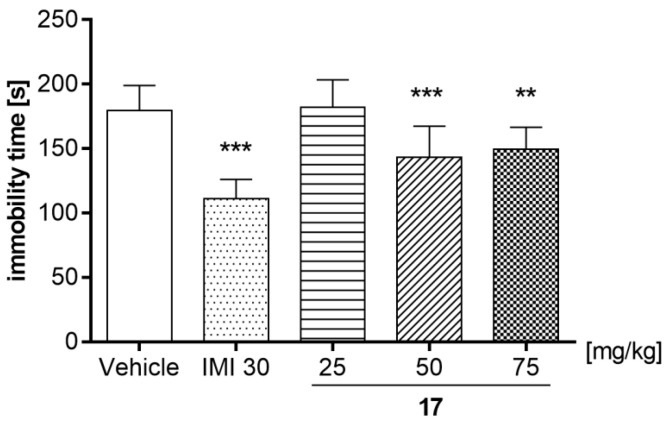
Effects of an acute administration of compound **17** in the forced swim test in mice. **17** (25, 50, and 75 mg/kg), imipramine hydrochloride (IMI, 30 mg/kg), and vehicle were administered i.p. 60 min before the test. The obtained data are presented as the mean ± SEM (*n* = 10 animals per group). ** *p* < 0.01, *** *p* < 0.001 versus the vehicle group (one-way ANOVA followed by Bonferroni’s post-hoc test).

In order to determine its antidepressant-like activity, compound **17** was used in the following doses: 25, 50, and 75 mg/kg (Figure 13) [one-way ANOVA: F (4, 45) = 21.67; *p* < 0.0001]. Statistical analysis of the results showed that **17** used at a dose of 25 mg/kg had no statistically significant effect (*p* > 0.05) on the immobility time in mice. However, the test compound administered at a dose of 50 and 75 mg/kg significantly reduced the total time of immobility in comparison with the control group.

The results obtained in the FST test indicated that **17** showed antidepressant-like properties, observed as a reduction in the immobility time of the mice, but differently and depending on the dose—only higher doses of the compound were active, 50 mg/kg (*p* < 0.001) and 75 mg/kg (*p* < 0.01; Figure 13). In addition, imipramine (IMI) at the dose of 30 mg/kg, used as a reference drug, caused a statistically significant decrease in immobility time (*p* < 0.001). FST is a simple and fast test established in experimental pharmacology for detecting the antidepressant effect of tested substances [45]. The mouse placed in a beaker with water, without the possibility of escaping, initially makes intensive attempts to get out, but after a short time, it gives up and adopts an attitude known as immobility. The effect of **17** should be considered specific as the tested compound did not show a negative effect on the locomotor activity of mice at any tested dose (25, 50 and 75 mg/kg; Table 5). This suggests that the mice were able to cope with the stressful confined space swimming situation.

## 3. Conclusions

The compound D2AAK2 reported in our previous studies represents an interesting case of a highly selective ligand of the dopamine D_2_ receptor, which, moreover, does not follow the classical pharmacophore model for orthosteric ligands of this target. In order to investigate the binding mode of the studied compound to the D_2_ receptor and to elucidate which structural features are crucial for its affinity for this molecular target, we designed and synthesized 23 of its derivatives. Subsequent pharmacological evaluation and SAR studies demonstrated that the D2AAK2 structure tolerates only minor changes in the phenyl system to maintain its activity, while modifications of the benzothiazinone fragment and the linker connecting the two above groups are unfavorable and lead to the loss of activity. Selected derivatives were confirmed as D_2_ receptor antagonists, and the most active compound, having a comparable affinity for the D_2_ receptor as D2AAK2, showed antipsychotic potential in behavioral studies. As anxiety and depressive disorders often accompany schizophrenia, the selected compound has also been studied for its effects on these conditions, revealing its antidepressant potential in vivo. Our study demonstrates that D2AAK2 and its active derivatives constitute an interesting group of D_2_ receptor ligands, as they exhibit a unique mode of interacting with this target protein—they do not possess an amine nitrogen atom, which, positively charged at physiological pH, can interact electrostatically with Asp 3.32 of the binding pocket. Further research will allow us to better understand how the presented compounds interact with the target of interest and what features are responsible for their selectivity over closely related GPCRs.

## 4. Materials and Methods

### 4.1. Chemistry

The reagents used in the synthetic procedures were obtained from commercial sources or were available in-house. NMR spectra of the reported compounds were acquired on a Bruker AVANCE III 600 MHz apparatus (Bruker, Billerica, MA, USA). Chemical shifts (δ) are given in ppm, and are referenced to tetramethylsilane (TMS) or the residual proton signal in DMSO-*d*_6_, which was used as a solvent. Coupling constants (*J*) are measured in Hertz (Hz). Recorded spectral data were processed with MestReNova v.14.0.0 software (Mestrelab Research, Santiago de Compostela, Spain). High-resolution mass spectra (HRMS) of samples were acquired using an Agilent 1290 Infinity HPLC system (Agilent Technologies, Santa Clara, CA, USA) coupled to an Agilent 6550 iFunnel QTOF mass spectrometer (Agilent Technologies, Santa Clara, CA, USA) equipped with a Jet Stream Technology electrospray ion source (ESI). 0.1% formic acid in 50:50 *v/v* water:acetonitrile was used as a mobile phase, with a flow rate 0.4 mL/min. Samples were analyzed by flow injection analysis. Ions were acquired in positive ion mode (ESI+) from 100 to 1000 *m*/*z*. Two reference ions 121.0508 and 922.0097 were used for internal mass correction. Data were acquired using Mass Hunter Acquisition B.09 (Agilent Technologies, Santa Clara, CA, USA) and processed with Mass Hunter Qualitative B.07 software (Agilent Technologies, Santa Clara, CA, USA). NMR and HRMS spectra for the reported compounds are available in Supplementary Materials.

#### 4.1.1. General Procedure for Synthesis of the Intermediates **1a**–**15a**

A solution of 3-chloropropanoyl chloride or chloroacetyl chloride (20 mmol) in acetone (8 mL) was added dropwise to the refluxing solution of appropriate aniline (40 mmol) in acetone (16 mL). After all the substrates had been added, the mixture was refluxed for one hour and then cooled to room temperature. The resulting suspension was poured into 6M aqueous HCl (32 mL), which led to the precipitation of a given amide, which was then filtered off and dried.

#### 4.1.2. General Procedure for Synthesis of the Intermediates **1b**–**2b**

2-Aminothiophenol or 2-aminophenol (10 mmol) was stirred with 50 mL of water. Urea (15 mmol) was added to the resulting suspension and the mixture was refluxed for 36 h. After cooling down the obtained precipitate was filtered off, dissolved in DCM, and washed with 1M aqueous HCl and brine. The organic layer was then dried over anhydrous sodium sulfate and the solvent was removed under reduced pressure to give the solid product, which was used in the next step without further purification.

#### 4.1.3. Synthesis of the Intermediate **3b**

To the solution of 2-aminothiophenol (50 mmol) and ethyl chloroacetate (50 mmol) in ethanol 96° (150 mL), 10% water solution of potassium hydroxide (25 mL) was added, and the resulting reaction mixture was refluxed for 3 h, after which it was poured onto ice. The obtained solid was filtered off, washed with water, and then recrystallized from ethanol.

#### 4.1.4. General Procedure for Synthesis of the Final Compounds **1**–**3**, **5**, **7**–**23**

The appropriate intermediate **1a**–**15a** (2 mmol), the corresponding amide (2 mmol), and anhydrous potassium carbonate (4 mmol) were placed in a reaction vial, to which 6 mL of ACN was added, and the resulting mixture was stirred and refluxed overnight. After cooling down the reaction was filtered and the solvent was distilled off on a rotavap. The obtained crude product was purified by washing with ACN, recrystallization from ACN or MeOH, or by DCVC (hexane/ethyl acetate).

#### 4.1.5. Synthesis of the Final Compound **4**

3-Chloro-*N*-(4-(trifluoromethyl)phenyl)propanamide (1 mmol), 2,3-dihydrobenzo[*b*][1,4]oxazepin-4(5*H*)-one (1 mmol) and anhydrous potassium carbonate (2 mmol) were placed in a reaction vial, to which 4 mL of DMF was added, and the resulting mixture was stirred overnight at room temperature. After that the reaction mixture was concentrated on a rotavap and the resulting residue was dissolved in ethyl acetate and washed with water. The organic layer was dried over anhydrous sodium sulfate and the solvent was evaporated. The obtained crude product was purified by DCVC (hexane/ethyl acetate 1:1).

#### 4.1.6. Synthesis of the Final Compound **6**

3-Chloro-*N*-(4-(trifluoromethyl)phenyl)propanamide (1 mmol), piperidin-2-one (2 mmol), and potassium hydroxide (4 mmol) were placed in a reaction vial, to which 4 mL of DMSO was added, and the resulting mixture was stirred overnight at room temperature. After that 15 mL of water was added to the reaction mixture and it was extracted three times with ethyl acetate. Combined organic layers were washed with water and brine, and dried over anhydrous sodium sulfate. Evaporation of the solvent gave the crude product which was purified by washing with ACN.

#### 4.1.7. Purification and Spectral Data for the Synthesized Final Compounds **1**–**23**

*3-(3-Oxo-2,3-dihydro-4H-benzo[b][1,4]oxazin-4-yl)-N-(4-(trifluoromethyl)phenyl)propanamide* (**1**). Compound purified by crystallization from ACN: white solid. Yield: 38%. ^1^H NMR (600 MHz, DMSO) δ 10.41 (s, 1H), 7.77 (d, *J* = 8.5 Hz, 2H), 7.67 (d, *J* = 8.5 Hz, 2H), 7.31 (d, *J* = 8.0 Hz, 1H), 7.07 (dq, *J* = 8.5, 4.3 Hz, 1H), 7.02 (d, *J* = 4.4 Hz, 2H), 4.64 (s, 2H), 4.22 (t, *J* = 7.4 Hz, 2H), 2.70 (t, *J* = 7.4 Hz, 2H). ^13^C NMR (151 MHz, DMSO) δ 169.92, 164.49, 145.45, 143.01, 128.78, 126.49 (q, *J* = 3.9 Hz), 124.85 (q, *J* = 271.3 Hz), 124.05, 123.69 (q, *J* = 32.6 Hz), 123.25, 119.50, 117.12, 115.83, 67.54, 37.47, 34.69. HRMS (ESI) *m/z* [M + H]^+^ calculated for C_18_H_15_F_3_N_2_O_3_: 365.1108, found: 365.1109.
*3-(2-Oxobenzo[d]thiazol-3(2H)-yl)-N-(4-(trifluoromethyl)phenyl)propanamide* (**2**). Compound was purified by washing with ACN: white solid. Yield: 34%. ^1^H NMR (600 MHz, DMSO) δ 10.45 (s, 1H), 7.75 (d, *J* = 8.4 Hz, 2H), 7.66 (dd, *J* = 8.4, 3.1 Hz, 3H), 7.45 (d, *J* = 8.1 Hz, 1H), 7.38 (t, *J* = 7.8 Hz, 1H), 7.20 (t, *J* = 7.6 Hz, 1H), 4.27 (t, *J* = 7.0 Hz, 2H), 2.80 (t, *J* = 7.0 Hz, 2H). ^13^C NMR (151 MHz, DMSO) δ 169.67, 169.18, 142.91, 137.18, 127.04, 126.51 (q, *J* = 3.7 Hz), 124.82 (q, *J* = 270.8 Hz), 123.73 (q, *J* = 32.0 Hz), 123.60, 123.35, 121.93, 119.51, 111.99, 39.27, 35.03. HRMS (ESI) *m/z* [M + H]^+^ calculated for C_17_H_13_F_3_N_2_O_2_S: 367.0723, found: 367.0724.
*3-(2-Oxobenzo[d]oxazol-3(2H)-yl)-N-(4-(trifluoromethyl)phenyl)propanamide* (**3**). Compound purified by crystallization from ACN: yellowish solid. Yield: 43%. ^1^H NMR (600 MHz, DMSO) δ 10.42 (s, 1H), 7.73 (d, *J* = 8.5 Hz, 2H), 7.65 (d, *J* = 8.6 Hz, 2H), 7.36–7.31 (m, 2H), 7.24–7.18 (m, 1H), 7.14–7.09 (m, 1H), 4.14 (t, *J* = 6.7 Hz, 2H), 2.86 (t, *J* = 6.7 Hz, 2H). ^13^C NMR (151 MHz, DMSO) δ 169.71, 154.06, 142.85, 142.39, 131.39, 126.47 (q, *J* = 4.0 Hz), 124.78 (q, *J* = 272.0 Hz), 124.23, 123.71 (q, *J* = 32.0 Hz), 122.58, 119.46, 110.02, 109.93, 38.66, 34.89. HRMS (ESI) *m/z* [M + H]^+^ calculated for C_17_H_13_F_3_N_2_O_3_: 351.0951, found: 351.0950.
*3-(4-Oxo-3,4-dihydrobenzo[b][1,4]oxazepin-5(2H)-yl)-N-(4-(trifluoromethyl)phenyl)propanamide* (**4**). Compound purified by DCVC (hexane/ethyl acetate 1:1): yellowish solid. Yield: 27%. ^1^H NMR (600 MHz, DMSO) δ 10.29 (s, 1H), 7.71 (d, *J* = 8.5 Hz, 2H), 7.64 (d, *J* = 8.5 Hz, 2H), 7.50 (dt, *J* = 7.8, 1.6 Hz, 1H), 7.24 (dtt, *J* = 20.0, 7.5, 1.5 Hz, 2H), 7.12 (dt, *J* = 7.7, 1.5 Hz, 1H), 4.45 (td, *J* = 6.7, 1.3 Hz, 2H), 4.10–3.98 (m, 2H), 2.61–2.57 (m, 2H), 2.52 (t, *J* = 4.5 Hz, 2H). ^13^C NMR (151 MHz, DMSO) δ 170.69, 169.96, 150.18, 143.00, 136.99, 127.54, 126.43 (q, *J* = 3.9 Hz), 125.76, 124.21, 123.69 (q, *J* = 271.2 Hz), 123.68 (q, *J* = 31.9 Hz), 123.20, 119.41, 74.56, 43.82, 35.49, 35.00. HRMS (ESI) *m/z* [M + H]^+^ calculated for C_19_H_17_F_3_N_2_O_3_: 379.1264, found: 379.1266.
*3-(3-Oxothiomorpholino)-N-(4-(trifluoromethyl)phenyl)propanamide* (**5**). Compound was purified by washing with ACN: white solid. Yield: 20%. ^1^H NMR (600 MHz, DMSO) δ 10.39 (s, 1H), 7.80 (d, *J* = 8.5 Hz, 2H), 7.67 (d, *J* = 8.5 Hz, 2H), 3.67–3.58 (m, 4H), 3.23 (s, 2H), 2.85 (t, *J* = 5.7 Hz, 2H), 2.63 (t, *J* = 6.9 Hz, 2H). ^13^C NMR (151 MHz, DMSO) δ 170.61, 167.07, 143.07, 126.52 (q, *J* = 3.7 Hz), 124.85 (q, *J* = 271.2 Hz), 123.67 (q, *J* = 31.9 Hz), 119.47, 49.40, 44.39, 35.59, 29.67, 26.39. HRMS (ESI) *m/z* [M + H]^+^ calculated for C_14_H_15_F_3_N_2_O_2_S: 333.0879, found: 333.0879.
*3-(2-Oxopiperidin-1-yl)-N-(4-(trifluoromethyl)phenyl)propanamide* (**6**). Compound was purified by washing with ACN: white solid. Yield: 24%. ^1^H NMR (600 MHz, DMSO) δ 10.37 (s, 1H), 7.79 (d, *J* = 8.4 Hz, 2H), 7.67 (d, *J* = 8.5 Hz, 2H), 3.54 (t, *J* = 7.1 Hz, 2H), 3.29 (t, *J* = 5.6 Hz, 2H), 2.59 (t, *J* = 7.0 Hz, 2H), 2.20 (t, *J* = 6.3 Hz, 2H), 1.72–1.61 (m, 4H). ^13^C NMR (151 MHz, DMSO) δ 170.81, 169.02, 143.13, 126.50 (q, *J* = 4.0 Hz), 124.85 (q, *J* = 271.0 Hz), 123.51 (q, *J* = 31.9 Hz), 119.43, 48.37, 43.81, 35.27, 32.47, 23.28, 21.39. HRMS (ESI) *m/z* [M + H]^+^ calculated for C_15_H_17_F_3_N_2_O_2_: 315.1315, found: 315.1315.
*3-((2-Methylphenyl)sulfonamido)-N-(4-(trifluoromethyl)phenyl)propanamide* (**7**). Compound purified by crystallization from ACN: white solid. Yield: 27%. ^1^H NMR (600 MHz, DMSO) δ 10.31 (s, 1H), 7.83 (dd, *J* = 8.2, 1.1 Hz, 1H), 7.76 (d, *J* = 8.6 Hz, 2H), 7.66 (d, *J* = 8.8 Hz, 2H), 7.50 (td, *J* = 7.5, 1.2 Hz, 1H), 7.41–7.35 (m, 2H), 3.08 (t, *J* = 7.0 Hz, 2H), 2.57 (s, 3H), 2.54 (t, *J* = 7.0 Hz, 2H). ^13^C NMR (151 MHz, DMSO) δ 169.97, 143.06 (d, *J* = 1.4 Hz), 138.94, 137.02, 132.97, 132.84, 128.89, 126.64, 126.44 (q, *J* = 3.7 Hz), 124.86 (q, *J* = 271.3 Hz), 123.57 (q, *J* = 31.9 Hz), 119.40, 38.92, 37.17, 20.23. HRMS (ESI) *m/z* [M + H]^+^ calculated for C_17_H_17_F_3_N_2_O_3_S: 387.0985, found: 387.0986.
*3-((4-Methylphenyl)sulfonamido)-N-(4-(trifluoromethyl)phenyl)propanamide* (**8**). Compound was purified by washing with ACN: white solid. Yield: 36%. ^1^H NMR (600 MHz, DMSO) δ 10.37 (s, 1H), 7.77 (d, *J* = 8.5 Hz, 2H), 7.72–7.63 (m, 4H), 7.39 (d, *J* = 8.0 Hz, 2H), 3.03 (t, *J* = 7.0 Hz, 2H), 2.53 (t, *J* = 7.1 Hz, 2H), 2.37 (s, 3H). ^13^C NMR (151 MHz, DMSO) δ 169.93, 143.10, 143.07, 137.87, 130.11, 127.03, 126.46 (q, *J* = 4.0 Hz), 124.85 (q, *J* = 271.3 Hz), 123.59 (q, *J* = 31.9 Hz), 119.40, 39.13, 37.14, 21.41. HRMS (ESI) *m/z* [M + H]^+^ calculated for C_17_H_17_F_3_N_2_O_3_S: 387.0985, found: 387.0986.
*2-(3-Oxo-2,3-dihydro-4H-benzo[b][1,4]thiazin-4-yl)-N-(4-(trifluoromethyl)phenyl)acetamide* (**9**). The compound precipitated after cooling down the reaction mixture, then purified by washing with ACN: beige solid. Yield: 37%. ^1^H NMR (600 MHz, DMSO) δ 10.70 (s, 1H), 7.82 (d, *J* = 8.5 Hz, 2H), 7.70 (d, *J* = 8.5 Hz, 2H), 7.44 (dd, *J* = 7.8, 1.5 Hz, 1H), 7.28 (ddd, *J* = 8.5, 7.4, 1.6 Hz, 1H), 7.16 (dd, *J* = 8.3, 1.2 Hz, 1H), 7.08 (td, *J* = 7.5, 1.2 Hz, 1H), 4.78 (s, 2H), 3.59 (s, 2H). ^13^C NMR (151 MHz, DMSO) δ 167.19, 166.12, 142.81, 140.20, 128.46, 127.81, 126.62 (q, *J* = 4.0 Hz), 124.84 (q, *J* = 269.5 Hz), 123.91, 123.86 (q, *J* = 31.5 Hz), 123.08, 119.50, 118.54, 48.86, 30.66. HRMS (ESI) *m/z* [M + H]^+^ calculated for C_17_H_13_F_3_N_2_O_2_S: 367.0723, found: 367.0724.
*3-(3-Oxo-2,3-dihydro-4H-benzo[b][1,4]thiazin-4-yl)-N-phenylpropanamide* (**10**). Compound was purified by washing with ACN: white solid. Yield: 47%. ^1^H NMR (600 MHz, DMSO) δ 10.00 (s, 1H), 7.59–7.53 (m, 2H), 7.42 (ddd, *J* = 10.9, 8.0, 1.3 Hz, 2H), 7.34–7.27 (m, 3H), 7.09–7.01 (m, 2H), 4.29–4.20 (m, 2H), 3.51 (s, 2H), 2.69–2.61 (m, 2H). ^13^C NMR (151 MHz, DMSO) δ 168.17, 164.20, 138.54, 138.40, 128.05, 127.54, 126.79, 122.74, 122.67, 122.58, 118.56, 117.48, 40.32, 33.88, 29.87. HRMS (ESI) *m/z* [M + H]^+^ calculated for C_17_H_16_N_2_O_2_S: 313.1005, found: 313.1005.
*3-(3-Oxo-2,3-dihydro-4H-benzo[b][1,4]thiazin-4-yl)-N-(2-(trifluoromethyl)phenyl)propanamide* (**11**). Compound was purified by washing with ACN: white solid. Yield: 42%. ^1^H NMR (300 MHz, DMSO) δ 9.71 (s, 1H), 7.76–7.62 (m, 2H), 7.49–7.36 (m, 4H), 7.36–7.28 (m, 1H), 7.07 (td, *J* = 7.5, 1.3 Hz, 1H), 4.22 (t, *J* = 7.5 Hz, 2H), 3.51 (s, 2H), 2.67 (t, *J* = 7.6 Hz, 2H). ^13^C NMR (151 MHz, DMSO) δ 170.29, 165.31, 139.59, 135.71, 133.39, 130.75, 128.66, 127.86, 127.25, 126.72 (q, *J* = 5.3 Hz), 125.43 (q, *J* = 30.6 Hz), 123.85 (q, *J* = 269.5 Hz), 123.85, 123.76, 118.47, 41.34, 34.13, 30.94. HRMS (ESI) *m/z* [M + H]^+^ calculated for C_18_H_15_F_3_N_2_O_2_S: 381.0879, found: 381.0878.
*3-(3-Oxo-2,3-dihydro-4H-benzo[b][1,4]thiazin-4-yl)-N-(o-tolyl)propanamide* (**12**). Compound purified by crystallization from ACN: white solid. Yield: 37%. ^1^H NMR (600 MHz, DMSO) δ 9.37 (s, 1H), 7.43 (td, *J* = 5.3, 2.8 Hz, 2H), 7.35 (d, *J* = 7.9 Hz, 1H), 7.34–7.29 (m, 1H), 7.18 (d, *J* = 7.4 Hz, 1H), 7.14 (td, *J* = 7.6, 1.6 Hz, 1H), 7.07 (ddd, *J* = 10.5, 5.2, 2.9 Hz, 2H), 4.25 (t, *J* = 7.5 Hz, 2H), 3.51 (s, 2H), 2.68 (t, *J* = 7.5 Hz, 2H), 2.16 (s, 3H). ^13^C NMR (151 MHz, DMSO) δ 168.16, 164.22, 138.52, 135.55, 131.04, 129.61, 127.55, 126.78, 125.22, 124.51, 124.47, 122.74, 122.69, 117.54, 40.37, 33.33, 29.90, 17.19. HRMS (ESI) *m/z* [M + H]^+^ calculated for C_18_H_18_N_2_O_2_S: 327.1162, found: 327.1163.
*3-(3-Oxo-2,3-dihydro-4H-benzo[b][1,4]thiazin-4-yl)-N-(m-tolyl)propanamide* (**13**). Compound purified by DCVC (hexane/ethyl acetate 1:1): white solid. Yield: 49%. ^1^H NMR (600 MHz, DMSO) δ 9.92 (s, 1H), 7.44–7.38 (m, 3H), 7.32 (ddd, *J* = 17.4, 8.2, 1.9 Hz, 2H), 7.16 (t, *J* = 7.8 Hz, 1H), 7.06 (td, *J* = 7.5, 1.1 Hz, 1H), 6.85 (d, *J* = 7.5 Hz, 1H), 4.28–4.18 (m, 2H), 3.51 (s, 2H), 2.64 (dd, *J* = 8.5, 6.6 Hz, 2H), 2.26 (s, 3H). ^13^C NMR (151 MHz, DMSO) δ 168.10, 164.20, 138.55, 138.32, 137.19, 127.89, 127.54, 126.79, 123.29, 122.74, 122.66, 119.13, 117.47, 115.79, 40.34, 33.89, 29.87, 20.58. HRMS (ESI) *m/z* [M + H]^+^ calculated for C_18_H_18_N_2_O_2_S: 327.1162, found: 327.1161.
*3-(3-Oxo-2,3-dihydro-4H-benzo[b][1,4]thiazin-4-yl)-N-(p-tolyl)propanamide* (**14**). Compound purified by DCVC (hexane/ethyl acetate 7:3): white solid. Yield: 38%. ^1^H NMR (600 MHz, DMSO) δ 9.91 (s, 1H), 7.46–7.39 (m, 4H), 7.31 (ddd, *J* = 8.4, 7.4, 1.6 Hz, 1H), 7.09 (d, *J* = 8.2 Hz, 2H), 7.06 (td, *J* = 7.5, 1.1 Hz, 1H), 4.29–4.17 (m, 2H), 3.51 (s, 2H), 2.70–2.58 (m, 2H), 2.24 (s, 3H). ^13^C NMR (151 MHz, DMSO) δ 167.91, 164.18, 138.55, 135.90, 131.47, 128.43, 127.54, 126.79, 122.74, 122.65, 118.59, 117.47, 40.37, 33.82, 29.86, 19.82. HRMS (ESI) *m/z* [M + H]^+^ calculated for C_18_H_18_N_2_O_2_S: 327.1162, found: 327.1170.
*N-(2-Chlorophenyl)-3-(3-oxo-2,3-dihydro-4H-benzo[b][1,4]thiazin-4-yl)propanamide* (**15**). Compound purified by DCVC (hexane/ethyl acetate 7:3): white solid. Yield: 42%. ^1^H NMR (600 MHz, DMSO) δ 9.63 (s, 1H), 7.66 (d, *J* = 8.2 Hz, 1H), 7.48 (d, *J* = 8.0 Hz, 1H), 7.45–7.39 (m, 2H), 7.31 (t, *J* = 7.8 Hz, 2H), 7.19 (t, *J* = 7.8 Hz, 1H), 7.07 (t, *J* = 7.5 Hz, 1H), 4.24 (t, *J* = 7.5 Hz, 2H), 3.51 (s, 2H), 2.73 (t, *J* = 7.6 Hz, 2H). ^13^C NMR (151 MHz, DMSO) δ 168.62, 164.21, 138.52, 134.15, 128.81, 127.55, 126.78, 126.70, 126.01, 125.82, 125.74, 122.74, 122.66, 117.48, 40.27, 33.33, 29.88. HRMS (ESI) *m/z* [M + H]^+^ calculated for C_17_H_15_ClN_2_O_2_S: 347.0616, found: 347.0616.
*N-(3-Chlorophenyl)-3-(3-oxo-2,3-dihydro-4H-benzo[b][1,4]thiazin-4-yl)propanamide* (**16**). Compound purified by washing with ACN: white solid. Yield: 28%. ^1^H NMR (600 MHz, DMSO) δ 10.19 (s, 1H), 7.77 (t, *J* = 2.0 Hz, 1H), 7.44–7.38 (m, 3H), 7.34–7.28 (m, 2H), 7.09 (ddd, *J* = 7.9, 2.1, 1.0 Hz, 1H), 7.06 (td, *J* = 7.6, 1.2 Hz, 1H), 4.27–4.20 (m, 2H), 3.51 (s, 2H), 2.69–2.62 (m, 2H). ^13^C NMR (151 MHz, DMSO) δ 168.61, 164.24, 139.80, 138.50, 132.39, 129.77, 127.55, 126.78, 122.76, 122.72, 122.30, 118.04, 117.49, 116.91, 40.15, 33.95, 29.88. HRMS (ESI) *m/z* [M + H]^+^ calculated for C_17_H_15_ClN_2_O_2_S: 347.0616, found: 347.0616.
*N-(4-Chlorophenyl)-3-(3-oxo-2,3-dihydro-4H-benzo[b][1,4]thiazin-4-yl)propanamide* (**17**). Compound purified by crystallization from MeOH: white solid. Yield: 21%. ^1^H NMR (600 MHz, DMSO) δ 10.14 (s, 1H), 7.61–7.55 (m, 2H), 7.42 (td, *J* = 7.3, 1.4 Hz, 2H), 7.38–7.33 (m, 2H), 7.33–7.27 (m, 1H), 7.06 (td, *J* = 7.6, 1.3 Hz, 1H), 4.24 (dd, *J* = 8.5, 6.7 Hz, 2H), 3.51 (s, 2H), 2.65 (dd, *J* = 8.6, 6.5 Hz, 2H). ^13^C NMR (151 MHz, DMSO) δ 168.35, 164.22, 138.51, 137.34, 127.97, 127.54, 126.78, 126.12, 122.75, 122.70, 120.08, 117.49, 40.21, 33.90, 29.87. HRMS (ESI) *m/z* [M + H]^+^ calculated for C_17_H_15_ClN_2_O_2_S: 347.0616, found: 347.0617.
*N-(2-Methoxyphenyl)-3-(3-oxo-2,3-dihydro-4H-benzo[b][1,4]thiazin-4-yl)propanamide* (**18**). Compound purified by washing with ACN: white solid. Yield: 41%. ^1^H NMR (600 MHz, DMSO) δ 9.25 (s, 1H), 7.89 (d, *J* = 8.0 Hz, 1H), 7.42 (dd, *J* = 8.2, 4.2 Hz, 2H), 7.31 (t, *J* = 7.9 Hz, 1H), 7.09–7.00 (m, 3H), 6.89 (t, *J* = 7.7 Hz, 1H), 4.21 (t, *J* = 7.5 Hz, 2H), 3.80 (s, 3H), 3.51 (s, 2H), 2.73 (t, *J* = 7.5 Hz, 2H). ^13^C NMR (151 MHz, DMSO) δ 168.38, 164.13, 149.11, 138.55, 127.51, 126.77, 126.50, 123.83, 122.70, 122.59, 121.65, 119.53, 117.49, 110.53, 54.99, 40.40, 33.63, 29.86. HRMS (ESI) *m/z* [M + H]^+^ calculated for C_18_H_18_N_2_O_3_S: 343.1111, found: 343.1112.
*N-(3-Methoxyphenyl)-3-(3-oxo-2,3-dihydro-4H-benzo[b][1,4]thiazin-4-yl)propanamide* (**19**). Compound purified by DCVC (hexane/ethyl acetate 7:3): white solid. Yield: 37%. ^1^H NMR (600 MHz, DMSO) δ 9.99 (s, 1H), 7.42 (td, *J* = 6.1, 5.6, 3.2 Hz, 2H), 7.36–7.30 (m, 1H), 7.27 (t, *J* = 2.3 Hz, 1H), 7.19 (t, *J* = 8.1 Hz, 1H), 7.07 (q, *J* = 7.8 Hz, 2H), 6.62 (dd, *J* = 8.3, 2.6 Hz, 1H), 4.23 (t, *J* = 7.6 Hz, 2H), 3.72 (s, 3H), 3.51 (s, 2H), 2.64 (t, *J* = 7.6 Hz, 2H). ^13^C NMR (151 MHz, DMSO) δ 168.23, 164.20, 158.85, 139.56, 138.53, 128.85, 127.54, 126.79, 122.75, 122.66, 117.46, 110.86, 108.04, 104.40, 54.34, 40.29, 33.92, 29.87. HRMS (ESI) *m/z* [M + H]^+^ calculated for C_18_H_18_N_2_O_3_S: 343.1111, found: 343.1111.
*N-(4-Methoxyphenyl)-3-(3-oxo-2,3-dihydro-4H-benzo[b][1,4]thiazin-4-yl)propanamide* (**20**). Compound purified by DCVC (hexane/ethyl acetate 7:3): white solid. Yield: 41%. ^1^H NMR (600 MHz, DMSO) δ 9.86 (s, 1H), 7.50–7.44 (m, 2H), 7.42 (t, *J* = 7.3 Hz, 2H), 7.31 (t, *J* = 7.8 Hz, 1H), 7.06 (t, *J* = 7.5 Hz, 1H), 6.93–6.82 (m, 2H), 4.22 (t, *J* = 7.6 Hz, 2H), 3.71 (s, 3H), 3.51 (s, 2H), 2.61 (t, *J* = 7.5 Hz, 2H). ^13^C NMR (151 MHz, DMSO) δ 167.63, 164.17, 154.56, 138.56, 131.56, 127.54, 126.79, 122.74, 122.64, 120.14, 117.47, 113.18, 54.53, 40.45, 33.75, 29.87. HRMS (ESI) *m/z* [M + H]^+^ calculated for C_18_H_18_N_2_O_3_S: 343.1111, found: 343.1113.
*N-(2-Nitrophenyl)-3-(3-oxo-2,3-dihydro-4H-benzo[b][1,4]thiazin-4-yl)propanamide* (**21**). Compound purified by DCVC (hexane/ethyl acetate 7:3): yellow solid. Yield: 37%. ^1^H NMR (600 MHz, DMSO) δ 10.36 (s, 1H), 7.94 (dd, *J* = 8.2, 1.5 Hz, 1H), 7.70 (ddd, *J* = 8.7, 7.4, 1.5 Hz, 1H), 7.60 (dd, *J* = 8.1, 1.4 Hz, 1H), 7.42 (dd, *J* = 7.7, 1.5 Hz, 1H), 7.37 (td, *J* = 8.4, 7.9, 1.4 Hz, 2H), 7.32 (ddd, *J* = 8.4, 7.3, 1.5 Hz, 1H), 7.07 (td, *J* = 7.5, 1.2 Hz, 1H), 4.25–4.16 (m, 2H), 3.51 (s, 2H), 2.74–2.62 (m, 2H). ^13^C NMR (151 MHz, DMSO) δ 169.55, 165.35, 142.97, 139.52, 134.39, 131.36, 128.65, 127.88, 125.94, 125.80, 125.32, 123.88, 123.78, 118.46, 41.12, 34.53, 30.93. HRMS (ESI) *m/z* [M + H]^+^ calculated for C_17_H_15_N_3_O_4_S: 358.0856, found: 358.0856.
*N-(3-Nitrophenyl)-3-(3-oxo-2,3-dihydro-4H-benzo[b][1,4]thiazin-4-yl)propanamide* (**22**). Compound purified by DCVC (hexane/ethyl acetate 7:3): yellow solid. Yield: 41%. ^1^H NMR (600 MHz, DMSO) δ 10.49 (s, 1H), 8.59 (t, *J* = 2.1 Hz, 1H), 7.90 (ddd, *J* = 8.2, 2.3, 1.0 Hz, 1H), 7.85 (ddd, *J* = 8.1, 2.1, 1.0 Hz, 1H), 7.60 (t, *J* = 8.2 Hz, 1H), 7.42 (ddd, *J* = 14.3, 8.0, 1.3 Hz, 2H), 7.32 (ddd, *J* = 8.5, 7.3, 1.6 Hz, 1H), 7.06 (td, *J* = 7.5, 1.1 Hz, 1H), 4.34–4.24 (m, 2H), 3.51 (s, 2H), 2.75–2.66 (m, 2H). ^13^C NMR (151 MHz, DMSO) δ 170.09, 165.36, 148.39, 140.53, 139.55, 130.62, 128.63, 127.85, 125.57, 123.85, 123.82, 118.57, 118.22, 113.71, 41.17, 35.10, 30.95. HRMS (ESI) *m/z* [M + H]^+^ calculated for C_17_H_15_N_3_O_4_S: 358.0856, found: 358.0855.
*N-(4-Nitrophenyl)-3-(3-oxo-2,3-dihydro-4H-benzo[b][1,4]thiazin-4-yl)propanamide* (**23**). Compound purified by DCVC (hexane/ethyl acetate 7:3): yellow solid. Yield: 33%. ^1^H NMR (600 MHz, DMSO) δ 10.60 (s, 1H), 8.26–8.19 (m, 2H), 7.83–7.76 (m, 2H), 7.42 (ddd, *J* = 11.3, 8.0, 1.4 Hz, 2H), 7.31 (ddd, *J* = 8.4, 7.3, 1.5 Hz, 1H), 7.06 (td, *J* = 7.5, 1.2 Hz, 1H), 4.36–4.21 (m, 2H), 3.51 (s, 2H), 2.76–2.68 (m, 2H). ^13^C NMR (151 MHz, DMSO) δ 170.37, 165.37, 145.61, 142.62, 139.53, 128.64, 127.86, 125.43, 123.86, 123.83, 119.24, 118.60, 41.03, 35.19, 30.95. HRMS (ESI) *m/z* [M + H]^+^ calculated for C_17_H_15_N_3_O_4_S: 358.0856, found: 358.0855.

### 4.2. Receptor Radioligand Binding Assays

Competition radioligand binding assays were performed on membranes isolated from cell lines stably expressing the cloned human receptors, which were either in-house or commercially available. CHO-K1 cell lines with stable expression of the cloned human dopamine D_1_ [46], D_2S_ [47] and D_3_ [46], and serotonin 5-HT_2A_ [48] receptors, as well as a HEK293 cell line with stable expression of the cloned human 5-HT_1A_ [10] and 5-HT_7_ [46] receptors, were used. Studied compounds were tested either at a single concentration of 10 μM or in competition binding curves, which were typically constructed with six different concentrations of a given compound. As internal controls, the following reference compounds were included in the assays: haloperidol (D_1_, D_2_, D_3_), 5-carboxamidotryptamine (5-CT) (5-HT_1A_), risperidone (5-HT_2A_) and methiothepin (5-HT_7_). Assays (250 μL assay final volume) were carried out in 96-well polypropylene plates in duplicate. In short, the suspension of appropriate membrane protein was incubated in an assay buffer with the addition of the corresponding radioligand, in the presence or absence of a tested compound or reference compound. [^3^H]- SCH-23390 (0.7 nM; D_1_ receptor), [^3^H]-Spiperone (0.6 nM for D_2_ receptor, 1 nM for D_3_ receptor), [^3^H]-8-OH-DPAT (1 nM; 5-HT_1A_ receptor), [^3^H]-Ketanserin (1.25 nM; 5-HT_2A_) and [^3^H]-SB269970 (2 nM; 5-HT_7_ receptor) were employed as radioligands. To assess non-specific binding, membrane protein, and radioligand were incubated in the presence of 1 μM (+)-butaclamol (D_1_) 100 μM sulpiride (D_2_), 1 µM haloperidol (D_3_), 10 μM serotonin (5-HT_1A_), 1 μM methysergide (5-HT_2A_) and 20 μM clozapine (5-HT_7_). After the incubation, the content of the test plates was transferred to 96 well plates with glass fiber filter GF/B or GF/C, previously pretreated with polyethyleneimine (PEI), and rapidly filtered with the use of vacuum, followed by rapid washing with cold (4 °C) wash buffer. After that, the filter plates were dried at 60 °C for 1 h. Then, 30 μL of liquid scintillation cocktail (UniverSol-ES, MP Biomedicals, Solon, OH, USA) was added to each well of the filter plates, and the radioactivity was measured in a MicroBeta^2^ microplate scintillation counter (PerkinElmer, Madrid, Spain). Table S2, which can be found in Supplementary Materials, shows the detailed conditions of experimental protocols applied in radioligand binding assays for each receptor.

#### Radioligand Binding Data Analysis

Competition binding curves were fitted to a one-site competition binding model (Fit Ki) with the use of Prism 7 software (GraphPad, San Diego, CA, USA). log*K*_i_ (log of the equilibrium dissociation constant (*K*_i_)) values were obtained after constraining the concentration of radioligand employed in the assay and its dissociation constant (*K*_D_) as determined in saturation radioligand binding assays.

### 4.3. Functional Assays of cAMP Signalling at D_2_ Receptors

The assessment of the activity of selected compounds at the D_2_ receptor was performed in cell-based functional assays of cAMP signaling, in the CHO-K1 cell line with stable expression of the cloned human D_2S_ receptor, which was also employed for the radioligand binding assays. For D_2_ receptor antagonism, test compounds were dispensed into an empty 96-well plate (Isoplate-96 Black Frame White Well, PerkinElmer España SL, Madrid, Spain) with the use of the dispensing noncontact Echo 550 acoustic liquid handler (LABCYTE Inc., San Jose, CA, USA). Compounds were handled from frozen 10^−2^ M stock solutions in 100% DMSO, from which serial 1000× solutions were prepared in 100% DMSO at the time of the assay, keeping vehicle concentration (0.1% DMSO) constant in the assay. Vials containing viable frozen cells were quickly thawed in a 37 °C water bath and cells were seeded into the assay plate in assay buffer (kit stimulation buffer containing 500 µM 3-isobutyl-1-methylxanthine (IBMX), which was directly added to the buffer as powder). After 5 min incubation at 37 °C, reference agonist dopamine (Dopamine hydrochloride, Merck Life Science S.L.U., Madrid, Spain) (10^−6^ M final concentration, prepared from freshly made aqueous stock solution) was added to the corresponding wells. After 10 min incubation at 37 °C, 10 µM forskolin was added to the corresponding wells, and incubation was continued for 5 min. After this time, cellular cAMP levels were quantified using the homogeneous time-resolved fluorescence (HTRF)-based cAMP Gs dynamic kit (Cisbio, Bioassays, Codolet, France) according to the manufacturer’s protocol. Haloperidol (10^−10^ M–10^−5^ M) was used as a control antagonist in these assays. Basal cAMP levels were determined in control wells in the absence of compound, agonist, and forskolin. Concentration (10^−10^ M–10^−4^ M) -response curves of dopamine were included in the individual experiments for internal EC_50_ calculation. Individual concentration-response curves of the compounds were fitted to the model of log(inhibitor) vs. response (three parameters) (Hill slope (n_H_) = 1; best fit in comparison to log(inhibitor) vs. response—Variable slope (four parameters) model, *p* < 0.05, extra sum-of-squares F test) described by the equation Y = Bottom + (Top-Bottom)/(1 + 10^((X-−LogIC50))) using Prism 7 software (GraphPad, San Diego, CA, USA) and pIC_50_ (-−logIC_50_) values were extracted from the fitting. *K*_b_ value (equilibrium dissociation constant of a competitive antagonist extracted from a functional assay) of the compounds was estimated according to the Leff-Dougall variant of the Cheng-Prusoff equation *K*_b_ = IC_50_/((2 + ([Ag]/[EC_50_])*^n^*)^1/*n*^ − 1), where IC_50_ is the concentration of antagonist that inhibits agonist response by a 50%; [Ag] is the concentration of agonist employed in the assay, [EC_50_] is the agonist EC_50_ value in the assay and n is the Hill slope of the concentration-response curve of the agonist [49].

### 4.4. Molecular Modeling

Ligands were modeled with the LigPrep module [50] of the Schrödinger suite of software, v. 2021-4. In order to find the protonation state, the Epik module [51,52] of the Schrödinger suite of software, v. 2021-4 was applied. Available X-ray structures of dopamine D_2_ receptor in complex with antagonists in inactive conformation were used for molecular docking (PDP ID: 6CM4 in complex with risperidone [15], PDB ID: 6LUQ in complex with haloperidol [16] and PDB ID: 7DFP in complex with spiperone [17]. The structures of the receptor were preprocessed using the Protein Preparation Wizard of Maestro Release 2021-4 [50] and mutations introduced to X-ray constructs were reversed. In particular receptor protonation states were assigned using the PROPKA module. The Standard Precision (SP) method of molecular docking with Glide [53] from Schrödinger release 2021-4 was applied. The grid files were obtained based on co-crystallized ligands. The selected receptor hydroxyl groups in the active site were made flexible as previously reported [54]. 50 poses were generated in the case of each ligand-receptor complex. PyMol 2.5.4 [55] software was used for the visualization of molecular modeling results.

### 4.5. X-ray Studies

A colorless single-crystal of **17** was selected for the X-ray diffraction analysis at room temperature. The measurement was made using an Oxford Diffraction Xcalibur CCD diffractometer (MoK*_α_*, *λ* = 0.71073 Å). The CrysAlis software [56] was used for data collection and reduction. Details of the X-ray crystal data structure determination and refinement are provided in Table S3. The structure was solved by direct method with SHELXS-2018 and refined by full matrix least-squares procedures with SHELXL-2018 program [57] within WinGX software [58]. Non-H atoms were found from different Fourier maps and refined anisotropically. H atoms attached to carbon were positioned geometrically (C–H = 0.93–0.97 Å) and refined as riding with U_iso_(H) = 1.2 U_eq_(C). The N–H hydrogen atom was found from the differential electron map and refined isotropically. The molecular graphics were created by using the ORTEP3 [58] and Mercury [59] programs. The geometrical calculations were performed using the PLATON program [60]. The CIF files have been deposited in the Cambridge Crystallographic Data Center (CCDC). These data can be obtained free of charge from The Cambridge Crystallographic Data Centre via www.ccdc.cam.ac.uk/data_request/cif (or from the CCDC, 12 Union Road, Cambridge CB2 1EZ, UK; Fax: +44-1223-336033; E-mail: deposit@ccdc.cam.ac.uk).

### 4.6. Behavioral Studies

#### 4.6.1. Animals

The experiments were carried out on 6 weeks old naive Swiss male mice (*Experimental Medicine* Center (*OMD*), Lublin, Poland), weighing 28–30 g. The cages with mice were located in air-conditioned rooms maintaining the ambient temperature within the range of 23–25 °C. The animals were provided with a natural 12 h day/night cycle, replicating the day and night mode. Each testing group was represented by 8–10 animals, depending on the research schedule. All experiments were conducted according to the National Institute of Health Guidelines for the Care and Use of Laboratory Animals (8th edition) and to the European Community Council Directive for the Care and Use of Laboratory Animals of 22 September 2010 (2010/63/EU) and were approved by the Local Ethical Committee (number of ethical approval: 147/2018).

#### 4.6.2. Drugs

Compound **17** (25, 50, 75 mg/kg) was suspended in 1% Tween 80 in saline solution and was administered intraperitoneally (i.p.) 60 min before the test. Diazepam (DZ, Relanium, Polfa, Poland) at a dose of 1 mg/kg was diluted in 0.9% saline containing 1% Tween 80 and was administered subcutaneously (s.c.), 60 min before the test. Imipramine hydrochloride (30 mg/kg, Sigma-Aldrich was administered intraperitoneally (i.p.) 60 min before the test. D-amphetamine sulfate (3 mg/kg; Sigma-Aldrich, USA) was given subcutaneously (s.c.). Prior to administration, the agent was dissolved in saline and injected 30 min before the test. Control animals received an (i.p.) or (s.c.) injection of a respective vehicle. All compounds were injected in a manner generally accepted in experimental pharmacology, in an amount of 10 mL/kg body weight.

#### 4.6.3. Motor Coordination Evaluated by Chimney and Rotarod Tests

Motor coordination has been assessed in mice using the chimney test and the rotarod test, according to the procedure we had used before [46]. Both tests were performed for 60 min. after injection of compound **17** (25, 50 and 75 mg/kg; i.p.) or vehicle (i.p.) (*n* = 8–10).

#### 4.6.4. Spontaneous Locomotor Activity and Amphetamine-Induced Hyperactivity

The measurement of the spontaneous locomotor activity was carried out using the OptoVarimex 4 Auto-Track device (Columbus Instruments, Columbus, OH, USA). Tested animals are kept in cages for 30 min. Their activity was recorded as interruptions of the light beams after 10 and 30 min of the test, and it was calculated automatically as a traveled distance in cm (±SEM). To determine whether compound **17** has an influence on amphetamine-induced hyperactivity, each mouse received **17** (25, 50 and 75 mg/kg; i.p.) and amphetamine (3 mg/kg; s.c.) 30 min after injection of **17** or vehicle and then spontaneous locomotor activity was measured.

#### 4.6.5. Elevated Plus-Maze (EPM) Test

The experiment was carried out on mice according to the method of Lister [61]. The plus-maze apparatus was made of black Plexiglas and consisted of two open (30 cm × 5 cm) and two enclosed (30 cm × 5 cm × 15 cm) arms. The arms extended from a central platform of 5 cm × 5 cm. The apparatus was mounted on a stable base raising it 45 cm above the floor and was illuminated by red light. The test consisted of placing a mouse in the center of the apparatus (facing an enclosed arm) and allowing it to freely explore the maze. The number of entries into the open arms and the time spent in these arms were scored for a 5-min test period. An entry was defined as placing all four paws within the boundaries of the arm. The following measures were obtained from the test: the total number of arm entries, the percentage of entries into the open arms, the time spent in the open arms expressed as a percentage of the time spent in both the open and closed arms. The anxiolytic activity was indicated by increases in time spent in open arms or in a greater number of open arm entries. The total number of entries into either type of an arm was used as a measure of the overall motor activity.

#### 4.6.6. Forced Swim Test (FST)

Forced Swim Test was carried out according to the method of Porsolt et al. [62]. It is a standard behavioral test, called the resignation test, which is used to determine the effectiveness of antidepressant drugs in laboratory rodents. Each mouse was placed individually for 6 min into a glass cylinder (height 25 cm, diameter 10 cm) with 15 cm of water at 23–25 °C. After the first 2 min of the test, the total duration of immobility (in seconds) was measured. An animal was judged to be immobile when it ceased struggling and remained floating motionless and making only movements allowing it to keep the head just above the surface of the water.

#### 4.6.7. Statistical Analysis

Statistical analysis of the obtained results was performed by either one-way or two-way analysis of variance (ANOVA) with Dunnett’s or Bonferroni’s multiple comparisons test. The outcomes were given as the means ± standard error of the mean (SEM). Between-group differences with *p* lower than 0.05 were considered statistically significant (where: * *p* < 0.05, ** *p* < 0.01).

## Figures and Tables

**Table 1:** Competition radioligand binding data at human D_2_ receptor. Data are expressed as % inh. at 10 μM, or and *K*_i_ (nM) when full displacement was achieved (mean ± SEM of 2–3 independent experiments performed in duplicate).

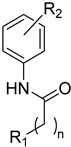					
	R_1_	R_2_	n	*hu*D_2_	
				% inh. at 10 µM	*K*_i_ [nM]
**1**	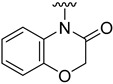	4-CF_3_	2	35 ± 14	
**2**	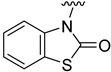	4-CF_3_	2	11 ± 5	
**3**	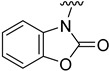	4-CF_3_	2	8 ± 8	
**4**	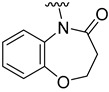	4-CF_3_	2		3846 ± 35
**5**	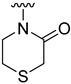	4-CF_3_	2	14 ± 10	
**6**	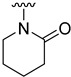	4-CF_3_	2	14 ± 11	
**7**	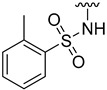	4-CF_3_	2	13 ± 13	
**8**	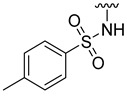	4-CF_3_	2	8 ± 7	
**9**	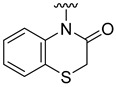	4-CF_3_	1	8 ± 8	
**10**		–	2	39 ± 4	
**11**		2-CF_3_	2	9 ± 1	
**12**		2-CH_3_	2	10 ± 0	
**13**		3-CH_3_	2	32 ± 4	
**14**		4-CH_3_	2		676 ± 134
**15**		2-Cl	2	9 ± 3	
**16**		3-Cl	2	42 ± 8	
**17**		4-Cl	2		381 ± 48
**18**		2-OCH_3_	2	6 ± 0	
**19**		3-OCH_3_	2	10 ± 4	
**20**		4-OCH_3_	2	47 ± 1	
**21**		2-NO_2_	2	5 ± 3	
**22**		3-NO_2_	2	27 ± 2	
**23**		4-NO_2_	2		724 ± 114
**D2AAK2**		4-CF_3_	2		321 ± 25 [10]
**Haloperidol**					6.56 ± 0.88

**Table 2:** Competition radioligand binding data for compounds **14**, **17**, and **23** at human-cloned D_1_, D_3_, 5-HT_1A_, 5-HT_2A_ and 5-HT_7_ receptors. Data (mean ± SEM of 2 independent experiments performed in duplicate) are expressed as % of inhibition of specific radioligand binding at 10 µM compound concentration (% inh.).

	*hu*D_1_		*hu*D_3_		*hu*5-HT_1A_		*hu*5-HT_2A_		*hu*5-HT_7_	
	% inh. at 10 µM	*K*_i_ (nM)	% inh. at 10 µM	*K*_i_ (nM)	% inh. at 10 µM	*K*_i_ (nM)	% inh. at 10 µM	*K*_i_ (nM)	% inh. at 10 µM	*K*_i_ (nM)
14	15 ± 2		8 ± 4		27 ± 2		15 ± 1		0	
17	21 ± 2		6 ± 3		29 ± 9		28 ± 1		5 ± 11	
23	12 ± 2		4 ± 3		22 ±3		23 ± 3		0	
Haloperidol		6.31 ± 0.79		4.95 ± 1.13						
5-CT						0.12 ± 0.02				
Risperidone								0.42 ± 0.03		
Methiothepin										2.78 ± 1.37

**Table 3:** Antagonist potency of selected compounds at D_2_ receptor. Compounds were evaluated in cell-based functional assays of second messenger production (inhibition of forskolin-stimulated cAMP production) in cells stably overexpressing the cloned human receptor. D_2_ antagonistic activity of the compounds was quantified as inhibition of 1 µM dopamine (DA) response. The table shows potency values, expressed as p*K*_b_ (-log*K_b_*) and *K_b_* (nM). *K*_b_ values were calculated from the inhibition concentration–response curves of the compounds against a single agonist concentration of 1 µM. The average EC_50_ value for DA in this assay was 203 nM (pEC_50_ DA (mean ± SEM) = 6.69 ± 0.14). Data shown in the table are mean ± SEM of three independent experiments performed in duplicate.

	D_2_ Signalling (cAMP)	
	p*K*_b_ (Mean ± SEM)	*K*_b_ (nM) (Mean)
**14**	6.93 ± 0.11	119
**17**	7.57 ± 0.19	27
**23**	6.95 ± 0.19	113
**Haloperidol**	9.97 ± 0.22	0.11

**Table 4:** Hydrogen-bond geometry for compound **17**.

Hydrogen Bond [Å, º]				
D–H···A	d(D–H)	d(H···A)	d(D···A)	∠ DHA
N2–H2N···O1 ^i^	0.82(3)	2.18(3)	2.962(4)	159(4)
C13–H13···O2	0.93	2.24	2.834(5)	121

Symmetry code: (i) x, y − 1, z.

**Table 5:** The effect of compound **17** on the spontaneous locomotor activity in mice. **17** and vehicle were administered intraperitoneally (i.p) 60 min before the test. Distance traveled was recorded after 10 and 30 min of the test. Each experimental group consisted of ten animals. The data are presented as the means ± SEM. The results were considered statistically significant if *p* < 0.05 (one-way ANOVA followed by Dunnett’s post hoc test).

Treatment	Distance Traveled (cm) after 10 min	Distance Traveled (cm) after 30 min
vehicle (control group)	1002 ± 104.0	2529 ± 460.6
**17** (25 mg/kg)	910.1 ± 139.3	2003 ± 355.7
**17** (50 mg/kg)	855.9 ± 195.1	2345 ± 616.0
**17** (75 mg/kg)	1200 ± 216.0	2983 ± 595.8

## Data Availability

The data that support the findings of this study are available in the Supplementary Materials of this article.

## Supplementary Materials

The following supporting information can be downloaded at: https://www.mdpi.com/article/10.3390/molecules28104211/s1, Figure S1. Packing diagrams of 17 viewed along the b-axis showing the formation of the infinite chains along [010] generated by N2-H2N···O2 hydrogen bonds; Figure S2. Part of the crystal structure of 17 showing C(8) infinite chain motif linked by N2-H2N···O2 hydrogen bonds propagating in the b-axis direction. Atoms involved in chain motif formation are shown in ball stick format. Symmetry code: (i) x, y−1, z; Table S1. Interatomic distances and selected bond angles for compound 17; Table S2. Detailed experimental conditions employed in radioligand binding assays; Table S3. Crystal data and structure refinement for compound 17; NMR and HRMS spectra of reported compounds.

## Sample Availability

Samples of the compounds are available from the authors.
