# Supplementary material for: Development and Characterization of Novel Selective, Non-Basic Dopamine D2 Receptor Antagonists for the Treatment of Schizophrenia
Source: Molecules. 2023 May 20;28(10):4211. doi: 10.3390/molecules28104211 (PMC10223470; doi:10.3390/molecules28104211)
Supplement: Supplementary file 1 [file molecules-28-04211-s001.zip › molecules-2401760-supplementary.pdf]

Supplementary Information

Development and characterization of novel selective, non-basic dopamine D_2 receptor antagonists for the treatment of schizophrenia

Piotr Stępnicki [1,*], Sylwia Wośko [2], Agata Bartyzel [3], Agata Zięba [1], Damian Bartuzi [1,4], Klaudia Szałaj [5], Tomasz M. Wróbel [1], Emilia Fornal [5], Jens Carlsson [4], Ewa Kędzierska [6], Ewa Poleszak [2], Marián Castro [7,8] and Agnieszka A. Kaczor [1,9,*]

[1] Department of Synthesis and Chemical Technology of Pharmaceutical Substances, Faculty of Pharmacy, Medical University of Lublin, 4A Chodźki St., PL-20093 Lublin, Poland;
[2] Laboratory of Preclinical Testing, Chair and Department of Applied and Social Pharmacy, Faculty of Pharmacy, Medical University of Lublin, Chodźki 1, PL-20093 Lublin, Poland;
[3] Department of General and Coordination Chemistry and Crystallography, Institute of Chemical Sciences, Faculty of Chemistry, Maria Curie-Skłodowska University in Lublin, Maria Curie-Skłodowska Sq. 2, PL-20031 Lublin, Poland;
[4] Science for Life Laboratory, Department of Cell and Molecular Biology, Uppsala University, 75124, Uppsala, Sweden;
[5] Department of Bioanalytics, Chair of Dietetics and Bioanalytics, Faculty of Biomedicine, Medical University of Lublin, Jaczewskiego 8b St., PL-20090 Lublin, Poland;
[6] Department of Pharmacology and Pharmacodynamics, Faculty of Pharmacy, Medical University of Lublin, 4A Chodźki St., PL-20093 Lublin, Poland;
[7] Center for Research in Molecular Medicine and Chronic Diseases (CiMUS), Universidade de Santiago de Compostela, Avda de Barcelona, E-15782 Santiago de Compostela, Spain;
[8] Instituto de Investigación Sanitaria de Santiago de Compostela (IDIS), Travesía da Choupana s/n, E-15706 Santiago de Compostela, Spain;
[9] School of Pharmacy, University of Eastern Finland, P.O. Box 1627, FI-70211 Kuopio, Finland;
* Correspondence: piotrstepnicki93@gmail.com (P.S.), agnieszka.kaczor@umlub.pl (A.A.K.)

Content:

Figure S1. Packing diagrams of **17** viewed along the *b*-axis showing formation of the infinite chains along [010] generated by N2-H2N···O2 hydrogen bonds.

Figure S2. Part of the crystal structure of **17** showing C(8) infinite chain motif linked by N2-H2N···O2 hydrogen bonds propagating in the *b*-axis direction. Atoms involved in chain motif formation are shown in ball stick format. Symmetry code: (i) x, y-1, z.

Table S1. Interatomic distances and selected bond angles for compound **17**.

Table S2. Detailed experimental conditions employed in radioligand binding assays.

Table S3. Crystal data and structure refinement for compound **17**.

NMR and HRMS spectra of reported compounds.

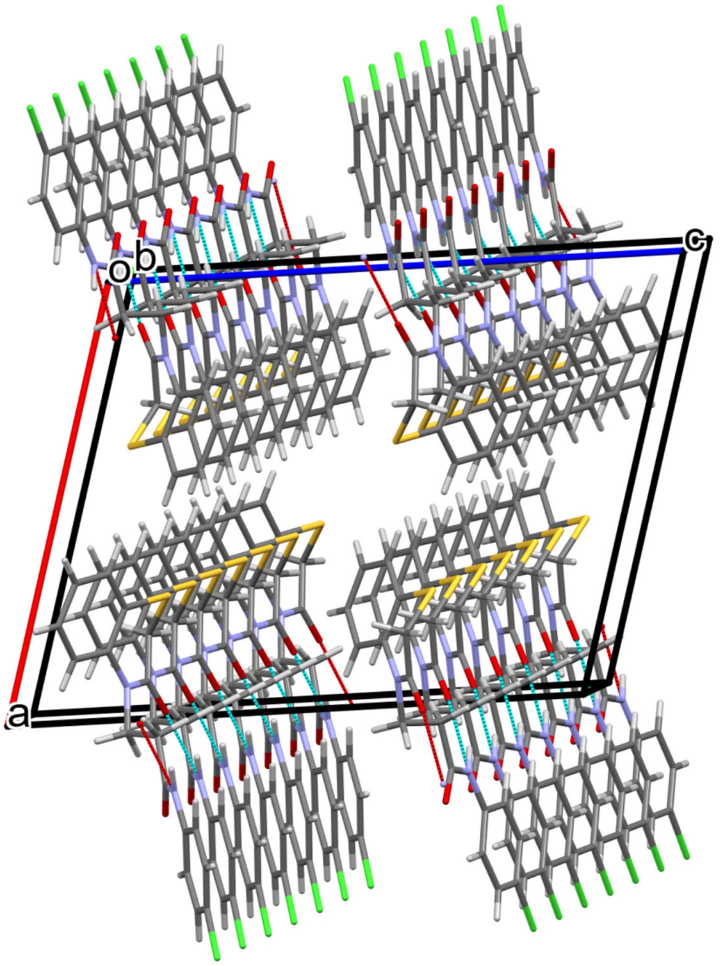

Figure S1. Packing diagrams of **17** viewed along the *b*-axis showing formation of the infinite chains along [010] generated by N2-H2N···O2 hydrogen bonds.

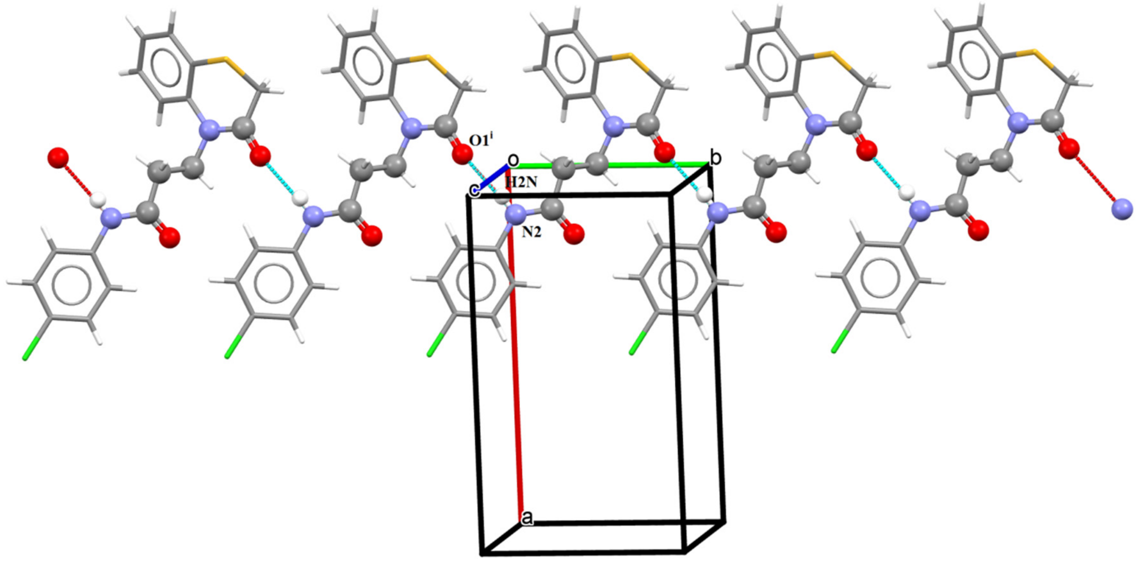

Figure S2. Part of the crystal structure of **17** showing C(8) infinite chain motif linked by N2-H2N···O2 hydrogen bonds propagating in the *b*-axis direction. Atoms involved in chain motif formation are shown in ball stick format. Symmetry code: (i) x, y-1, z.

Table S1. Interatomic distances and selected bond angles for compound **17**.

Bond lengths (Å)			
C1–O1	1.226(4)	C9–C10	1.507(5)
C1–N1	1.355(4)	C10–C11	1.509(5)
C1–C2	1.498(5)	O2–C11	1.204(4)
C2–S1	1.789(4)	C12–C17	1.384(4)
C3–C8	1.387(4)	C12–C13	1.385(4)
C3–C4	1.389(4)	C12–N2	1.414(4)
C3–S1	1.754(4)	C13–C14	1.376(4)
C4–C5	1.367(5)	C14–C15	1.375(5)
C5–C6	1.364(5)	C15–C16	1.365(5)
C6–C7	1.381(5)	C15–Cl1	1.740(4)
C7–C8	1.386(5)	C16–C17	1.381(5)
C8–N1	1.434(4)	N2–C11	1.341(5)
C9–N1	1.473(4)		
Bond angles (º)			
O1–C1–N1	122.2(3)	C14–C15–Cl1	119.4(3)
O1–C1–C2	121.1(4)	C11–N2–C12	128.8(3)
N1–C9–C10	111.3(3)	O2–C11–N2	122.7(4)
C16–C15–Cl1	120.2(3)	O2–C11–C10	122.1(4)
Torsion angles (º)			
C3–C8–N1–C1	-26.9(5)	C10–C9–N1–C8	-65.8(5)
C2–C1–N1–C8	-2.7(5)	C10–C9–N1–C1	109.7(4)
N1–C1–C2–S1	49.3(4)	C12–N2–C11–O2	3.3(8)
C1–C2–S1–C3	-59.2(3)	C9–C10–C11–O2	28.9(7)
C8–C3–S1–C2	35.1(3)	C17–C12–N2–C11	175.7(4)
S1–C3–C8–N1	3.2(5)	C13–C12–N2–C11	-5.3(7)

Table S2. Detailed experimental conditions employed in radioligand binding assays.

	hD_1	*h*D_2	*h*D_3	*h*5-HT_{1A}	*h*5-HT_{2A}	*h*5-HT_7
Protein per well	12 µg	35 µg	2 µg	10 µg	130 µg	2 µg
Assay buffer	50 mM Tris-HCl, 5 mM $MgCl_2$ (pH = 7.4)	50 mM Tris-HCl, 120 mM NaCl, 5 mM KCl, 5 mM $MgCl_2$, 1 mM EDTA (pH = 7.4)	50 mM Tris-HCl, 5 mM $MgCl_2$ (pH = 7.4)	50 mM Tris-HCl, 5 mM $MgSO_4$ (pH = 7.4)	50 mM Tris-HCl (pH = 7.5)	50 mM Tris-HCl, 4 mM $CaCl_2$, 1 mM L-Ascorbic acid, 0.1 mM Pargyline hydrochloride (pH = 7.4)
Radioligand	0.7 nM [^{3}H]- SCH-23390[a]	0.6 nM [^{3}H]-Spiperone[b]	1 nM [^{3}H]- Spiperone[b]	1 nM [^{3}H]-8-OH-DPAT[c]	1.25 nM [^{3}H]-Ketanserin[d]	2 nM [^{3}H]-SB269970[e]
Nonspecific binding	1 µM (+)-Butaclamol	100 µM Sulpiride	1 µM Haloperidol	10 µM Serotonin	1 µM Methysergide	20 µM Clozapine
Incubation	27ºC/60 min	25ºC/120 min	25ºC/60 min	37ºC/120 min	37ºC/30 min	37ºC/60 min
Filter plate	GF/C[f]	GF/C[f]	GF/C[f]	GF/C[f]	GF/B[g]	GF/C[f]
Filter plate pre-treatment	presoaked with 0.5% PEI[h] for 1 h and washed with 250 µl incubation buffer	presoaked with 0.5% PEI[h] for 1 h and washed with 250 µl incubation buffer	presoaked with 0.5% PEI[h] for 1 h and washed with 250 µl incubation buffer	presoaked with 0.5% PEI[h] for 1 h and washed with 250 µl incubation buffer	presoaked with 0.5% PEI[h] for 1 h and washed with 250 µl incubation buffer	presoaked with 0.5% PEI[h] for 1 h and washed with 250 µl incubation buffer
Wash buffer	50 mM Tris-HCl (pH = 7.4)	50 mM Tris-HCl, 0.9% NaCl (pH = 7.4)	50 mM Tris-HCl (pH = 7.4)	50 mM Tris-HCl (pH = 7.4)	50 mM Tris-HCl (pH = 6.6)	50 mM Tris-HCl, 4 mM $CaCl_2$, 1 mM L-Ascorbic acid, 0.1 mM Pargyline hydrochloride (pH = 7.4)
Washing steps	4 x 250 µl wash buffer	4 x 250 µl wash buffer	4 x 250 µl wash buffer	4 x 250 µl wash buffer	6 x 250 µl wash buffer	4 x 250 µl wash buffer

[a] 83.2 Ci/mmol, 1 mCi/ml, PerkinElmer NET930250UC. [b] 54.3 Ci/mmol, 1 mCi/ml, PerkinElmer NET1187250UC. [c] 200 Ci/mmol, 1 mCi/ml, PerkinElmer NET929250UC. [d] 47.3 Ci/mmol, 1 mCi/ml, PerkinElmer NET791250UC. [e] 32.7 Ci/mmol, 0.25 mCi/ml, PerkinElmer NET1198U250UC. [f] MultiScreen$_{HTS}$ FB Filter Plate, PerkinElmer MSFCN6. [g] MultiScreen$_{HTS}$ FB Filter Plate, PerkinElmer MSFBN6. [h] PEI, Polyethyleneimine.

Table S3. Crystal data and structure refinement for compound **17**.

Formula	$C_{17}H_{15}ClN_2O_2S$
Formula weight	346.82
Temperature K	298(2)
Crystal system	monoclinic
Space group	$P2_1/c$
a (Å)	13.5626(11)
b (Å)	7.2478(5)
c (Å)	17.2446(17)
β (°)	106.262(9)
Volume (Å^3)	1627.3(2)
Z	4
Calculated density (g cm^{-3})	1.416
μ (mm^{-1})	0.373
Absorption correction	multi-scan
F(000)	720
Crystal size (mm)	0.15 x 0.45 x 0.50
θ range (º)	2.461to 26.733
Limiting indices	-17≤h≤17, -9≤k≤8, -19≤l≤21
Reflections collected/unique	12931/3458
R_{int}	0.0860
Data/restraints/parameters	3458/0/213
GooF on F^2	1.029
Final R indices[I>2σ(I)]	R_1 = 0.0684, wR_2 = 0.1165
R indices(all data)	R_1 = 0.1636, wR_2 = 0.1551
Extinction coefficient	0.0031(7)
Largest diff. peak/hole, e Å^{-3}	0.406/-0.367
CCDC No.	2259221

3-(3-oxo-2,3-dihydro-4*H*-benzo[*b*][1,4]oxazin-4-yl)-*N*-(4-(trifluoromethyl)phenyl)propanamide (1)

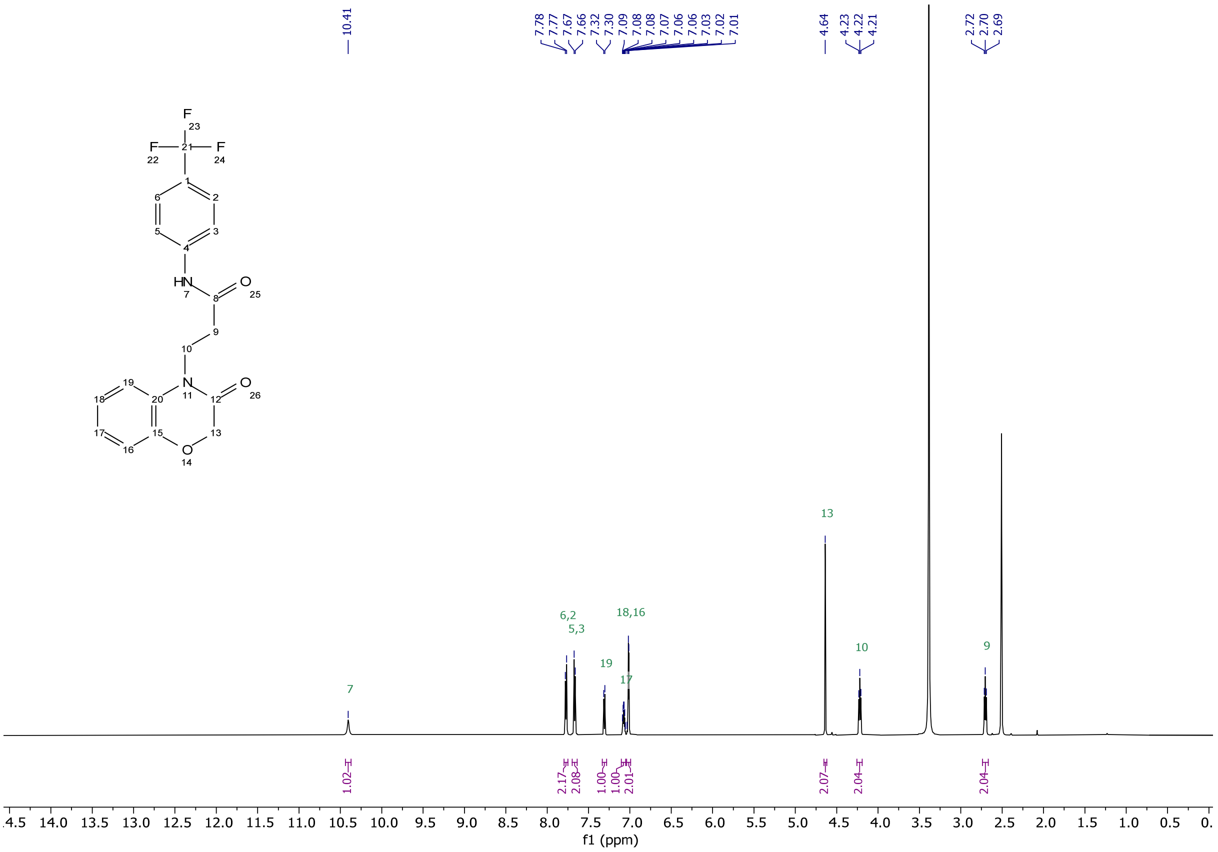

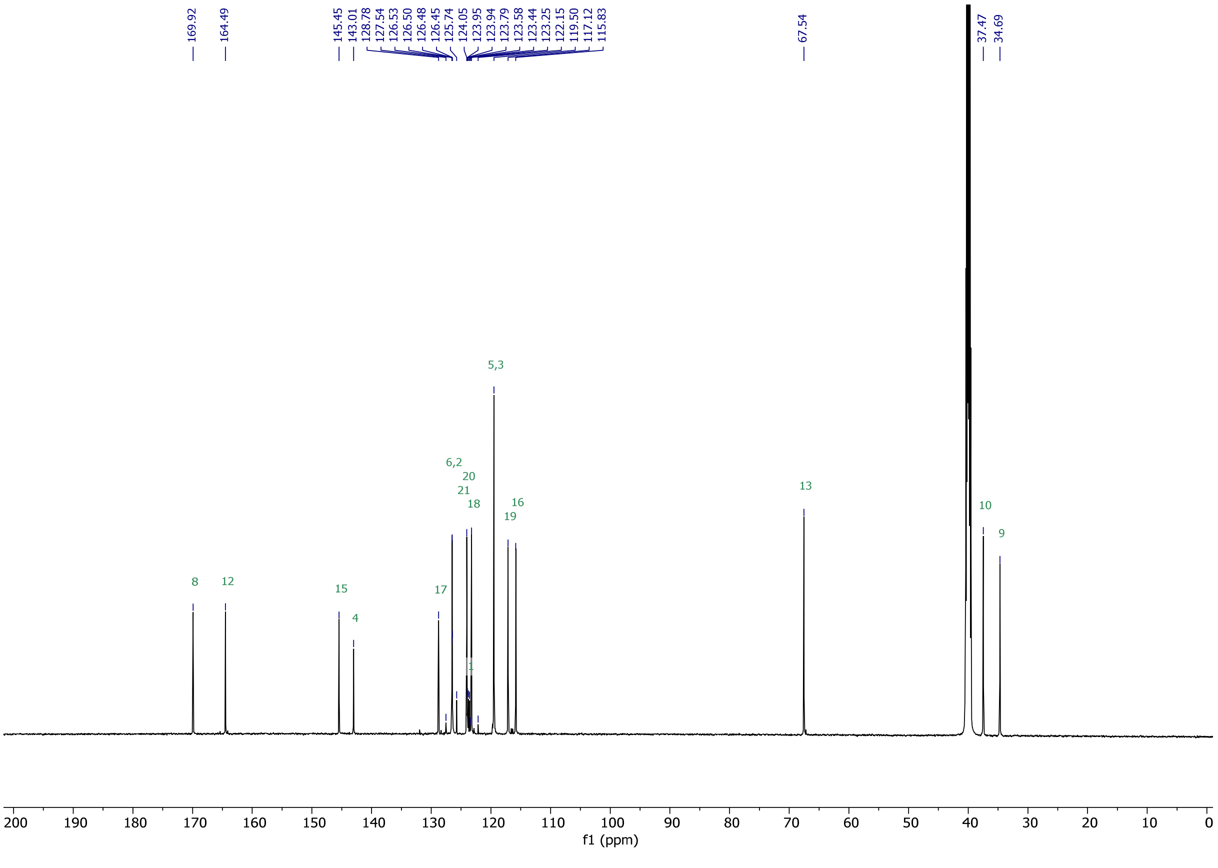

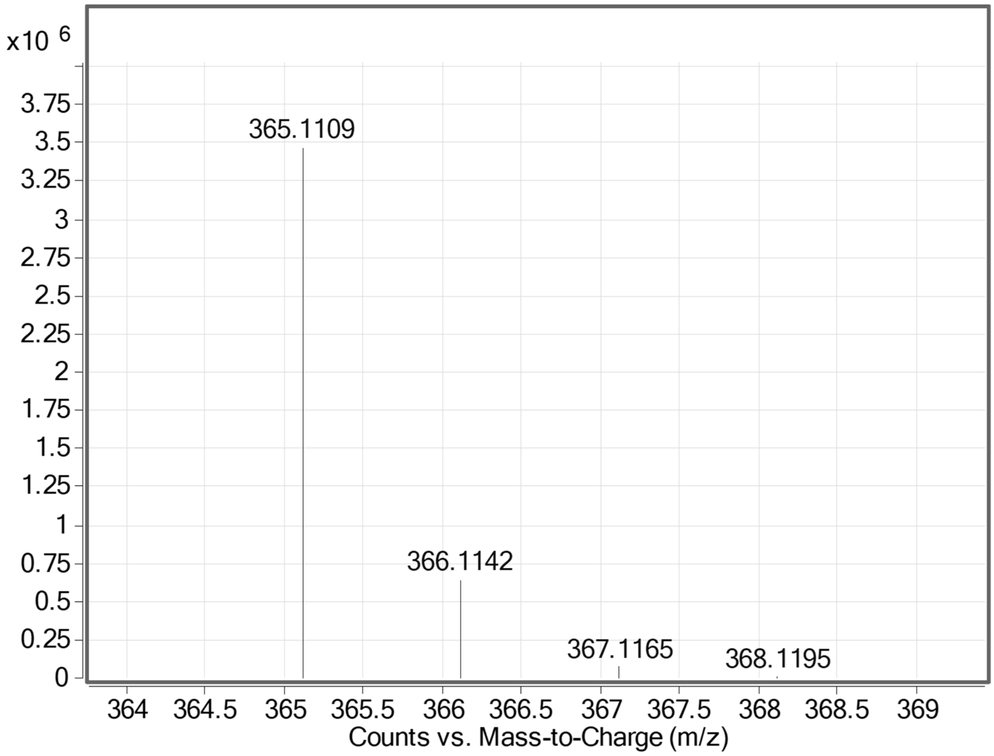
x10 6
3.75
3.5
3.25
3
2.75
2.5
2.25
2
1.75
1.5
1.25
1
0.75
0.5
0.25
0
365.1109
366.1142
367.1165
368.1195
364
364.5
365
365.5
366
366.5
367
367.5
368
368.5
369
Counts vs. Mass-to-Charge (m/z)

3-(2-oxobenzo[*d*]thiazol-3(2*H*)-yl)-*N*-(4-(trifluoromethyl)phenyl)propanamide (2)

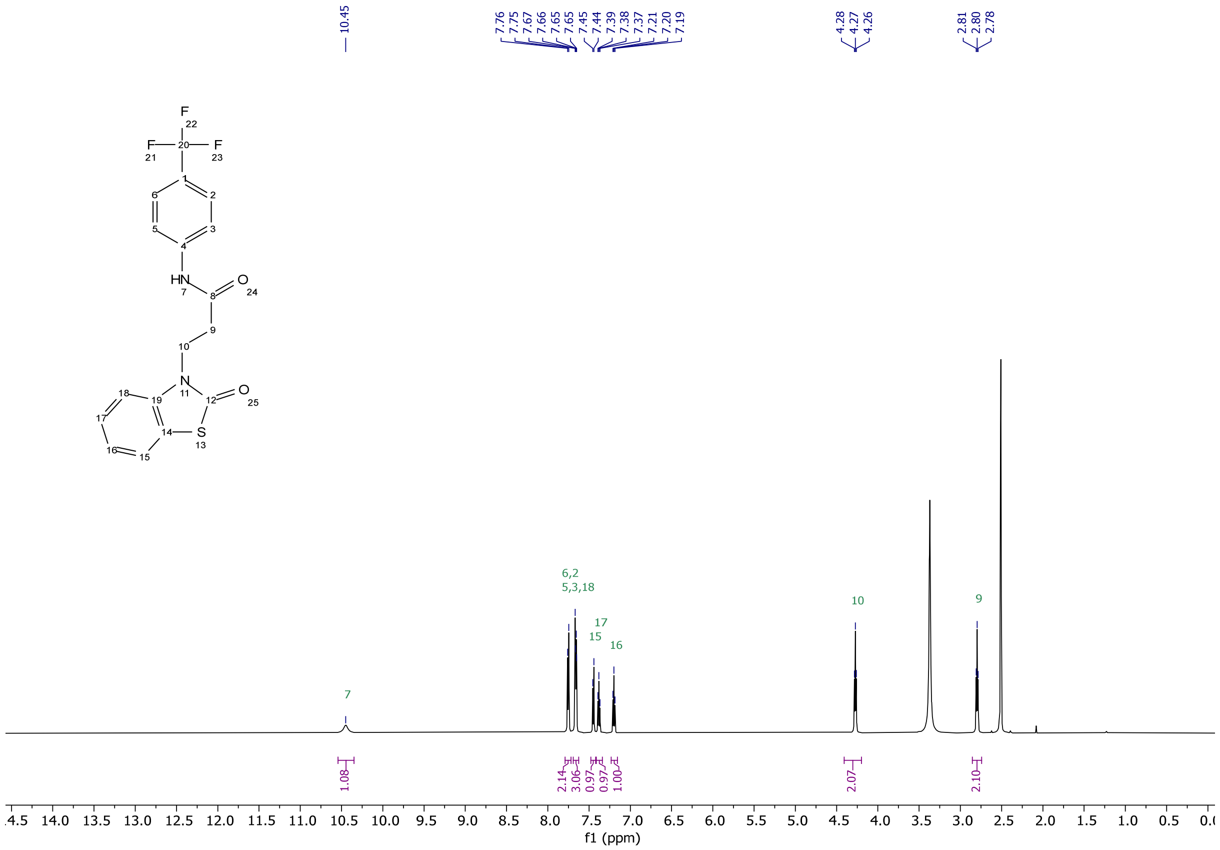

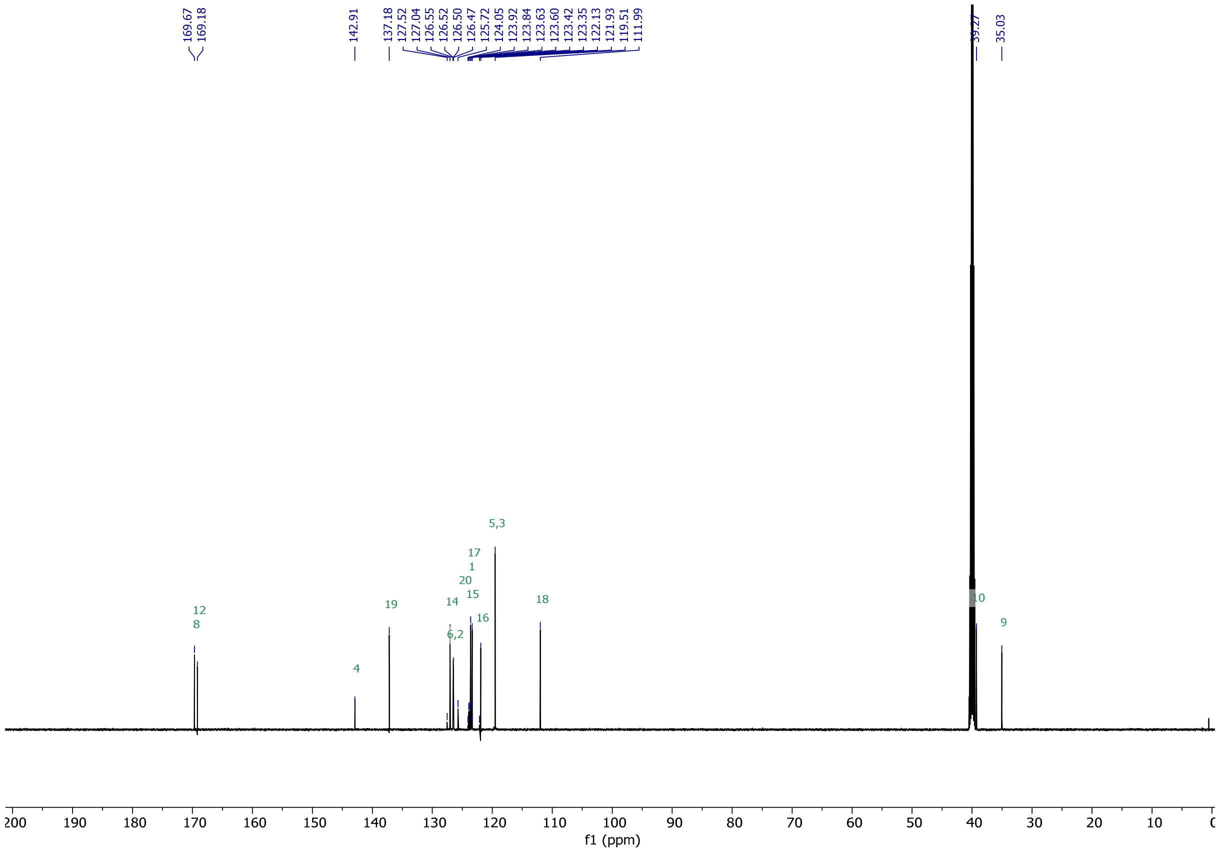

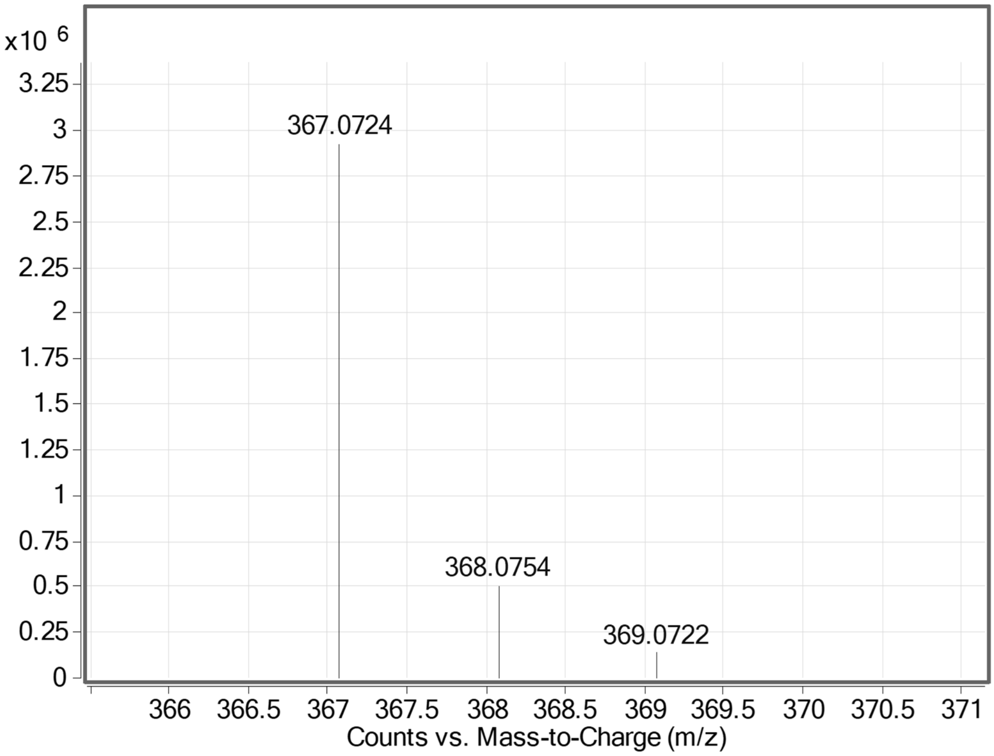
x10 6
3.25
3
2.75
2.5
2.25
2
1.75
1.5
1.25
1
0.75
0.5
0.25
0
367.0724
368.0754
369.0722
366
366.5
367
367.5
368
368.5
369
369.5
370
370.5
371
Counts vs. Mass-to-Charge (m/z)

3-(2-oxobenzo[*d*]oxazol-3(2*H*)-yl)-*N*-(4-(trifluoromethyl)phenyl)propanamide (3)

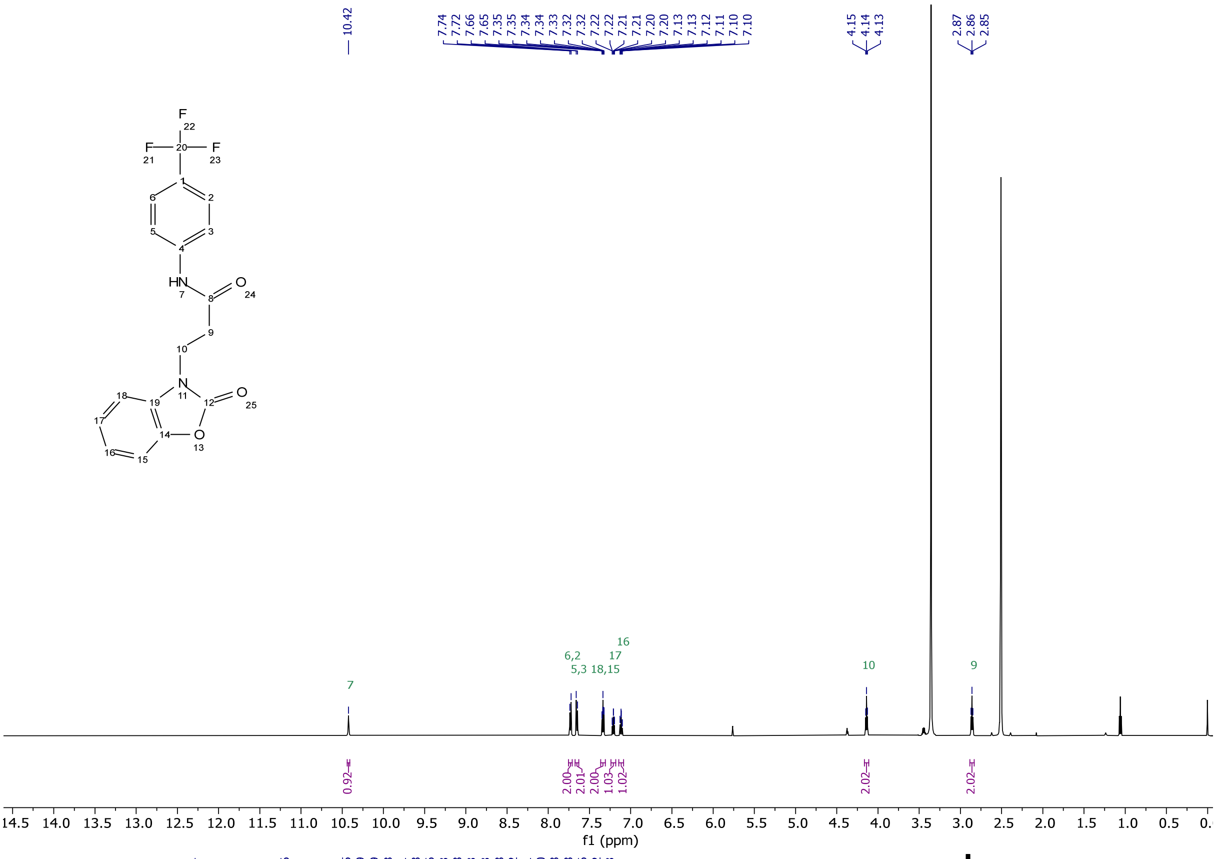

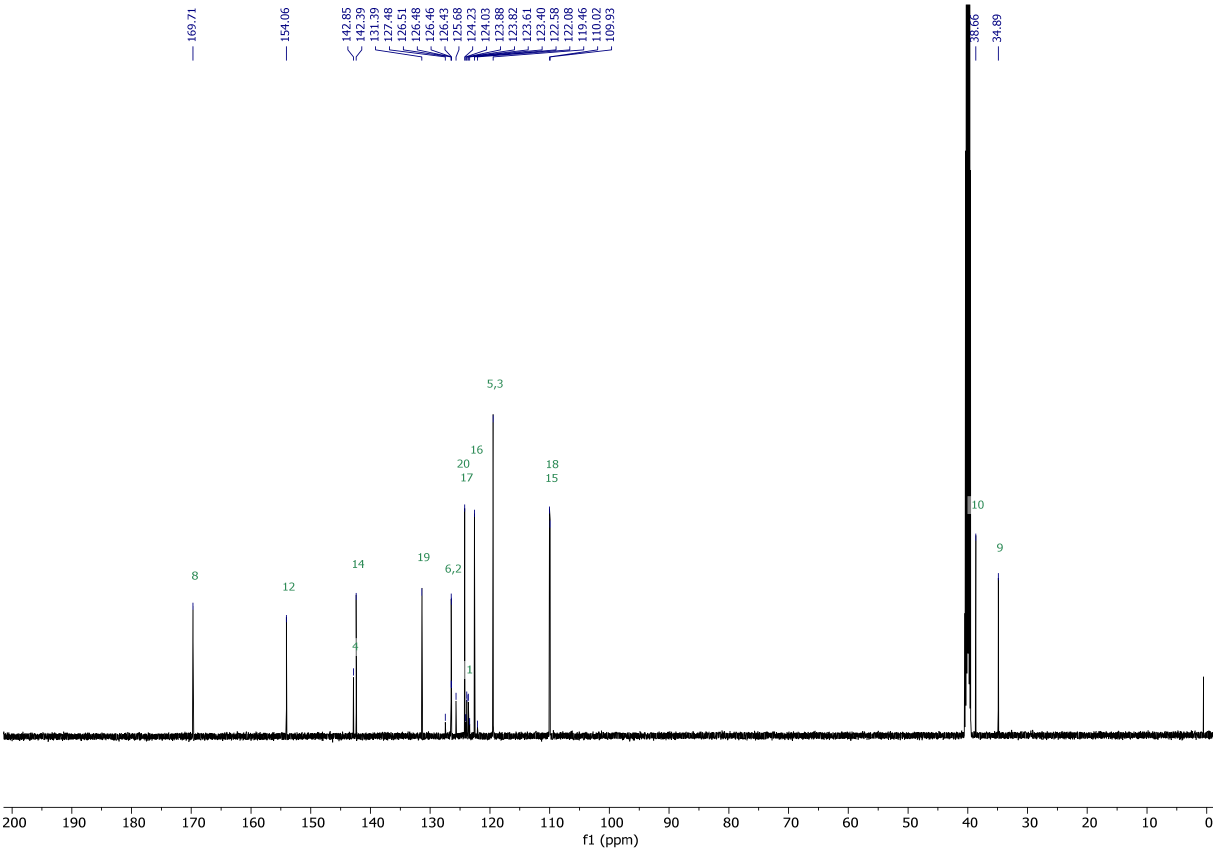

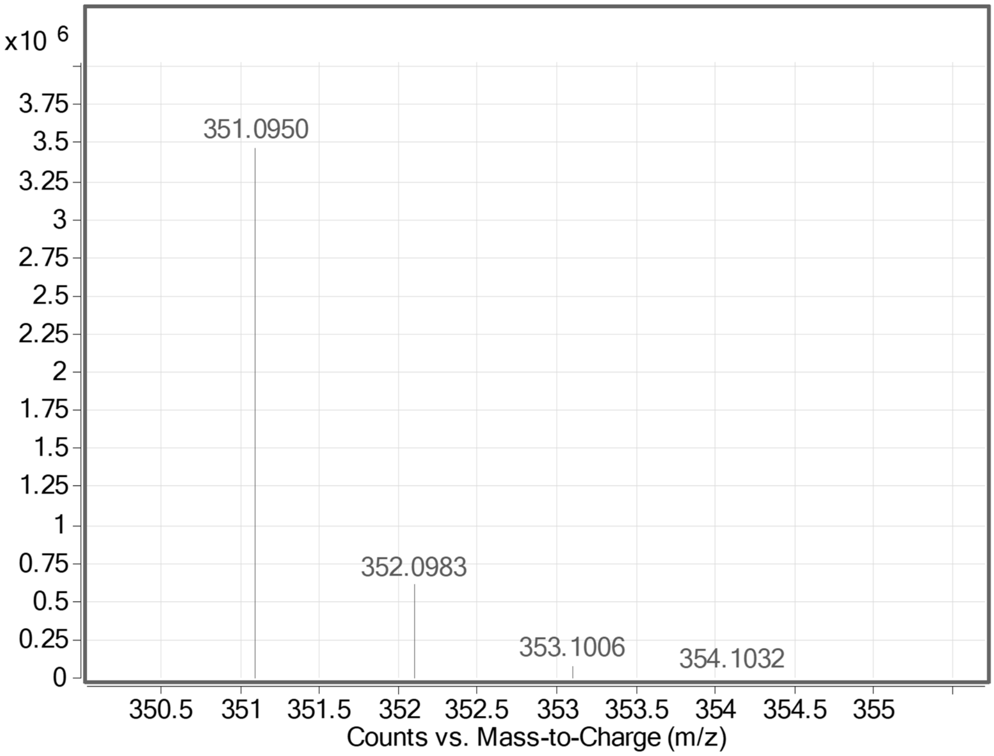

x10 6
3.75
3.5
3.25
3
2.75
2.5
2.25
2
1.75
1.5
1.25
1
0.75
0.5
0.25
0
351.0950
352.0983
353.1006
354.1032
350.5
351
351.5
352
352.5
353
353.5
354
354.5
355
Counts vs. Mass-to-Charge (m/z)

3-(4-oxo-3,4-dihydrobenzo[b][1,4]oxazepin-5(2*H*)-yl)-*N*-(4-(trifluoromethyl)phenyl)propanamide (4)

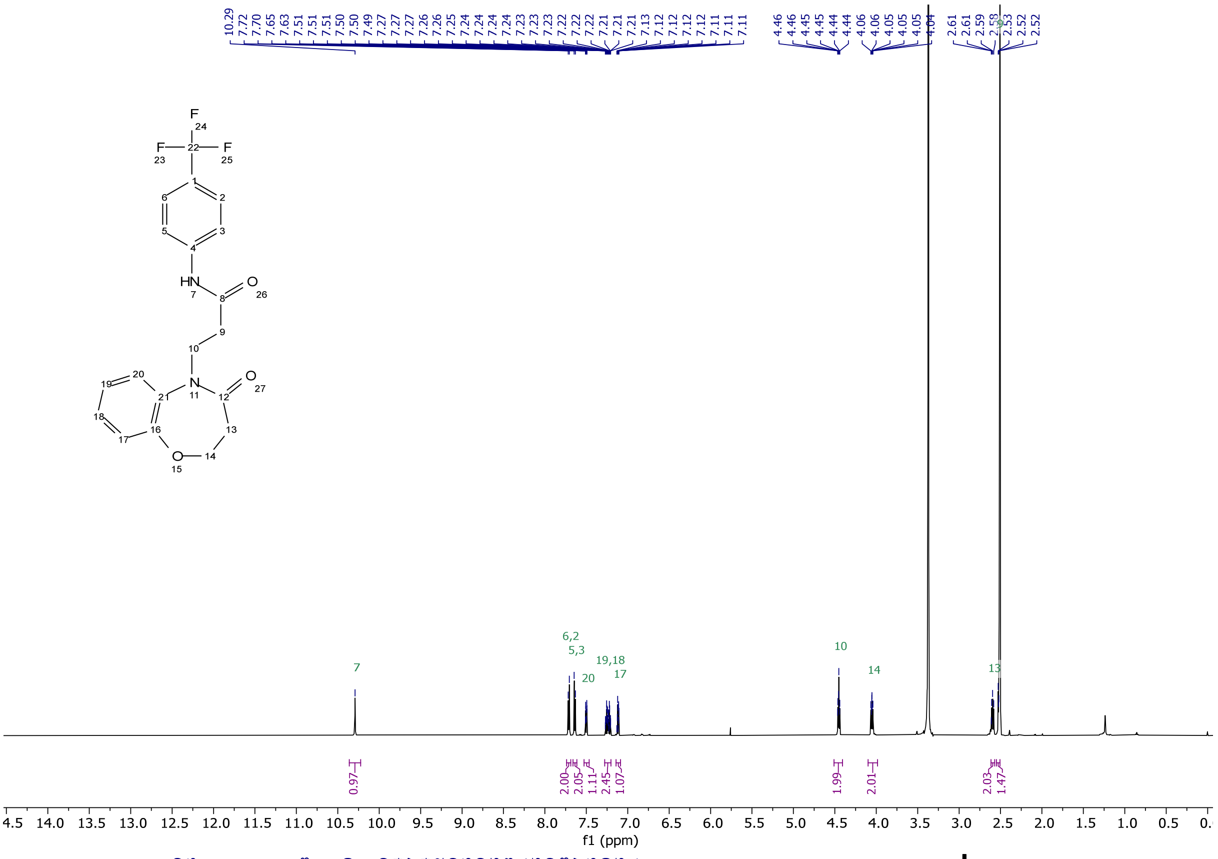

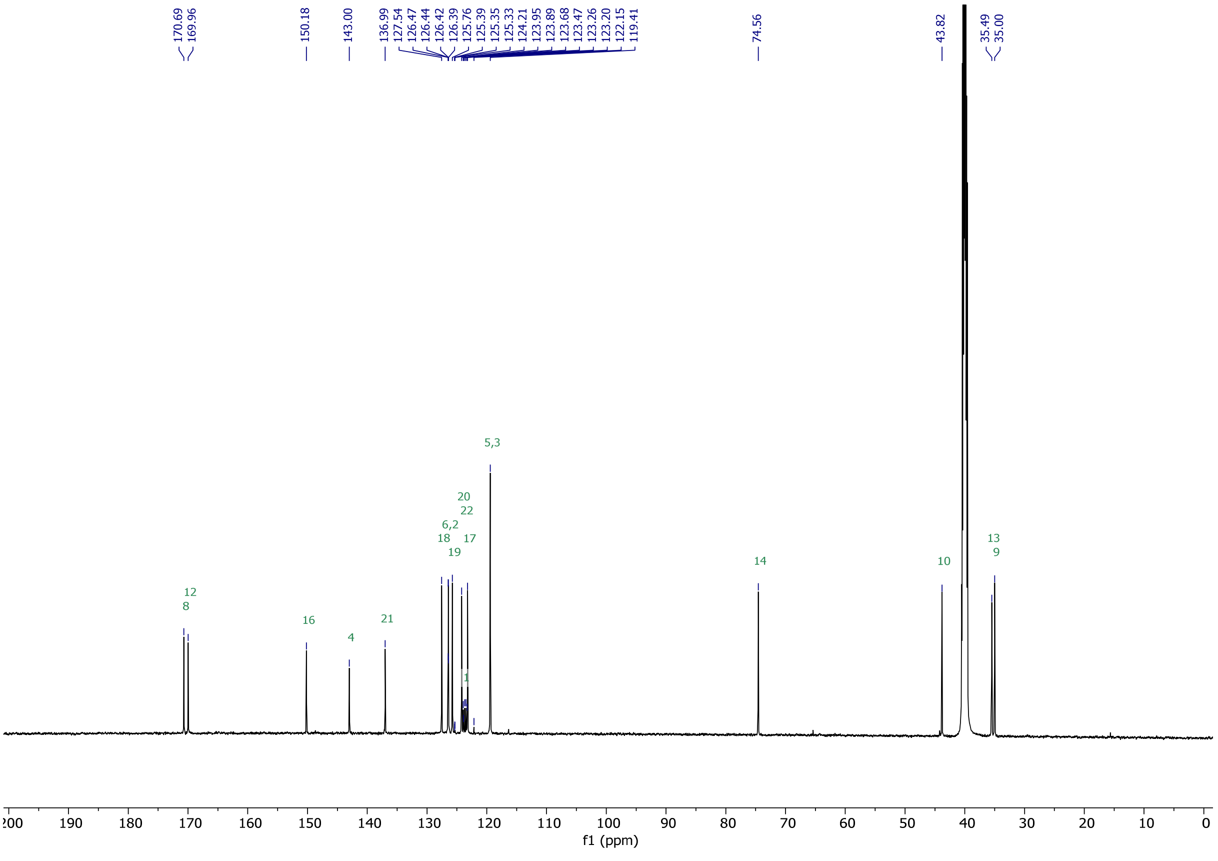

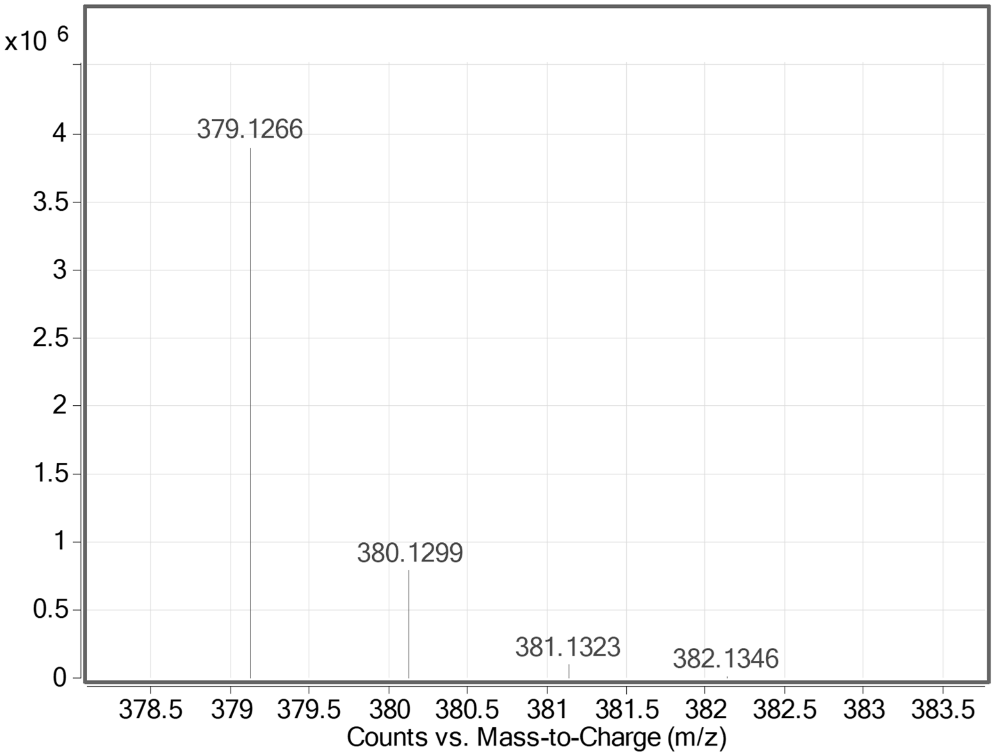
x10 6
4
3.5
3
2.5
2
1.5
1
0.5
0
379.1266
380.1299
381.1323
382.1346
378.5
379
379.5
380
380.5
381
381.5
382
382.5
383
383.5
Counts vs. Mass-to-Charge (m/z)

3-(3-oxothiomorpholino)-*N*-(4-(trifluoromethyl)phenyl)propanamide (5)

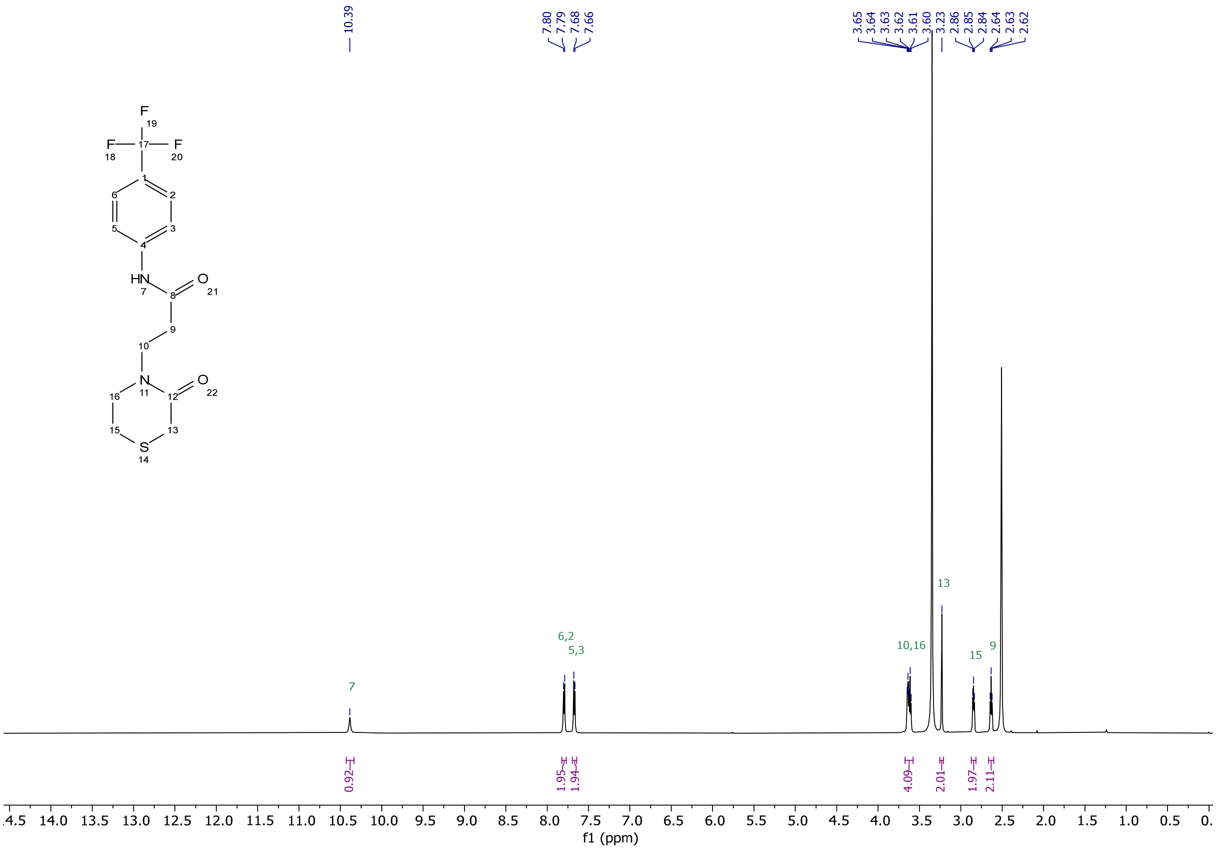

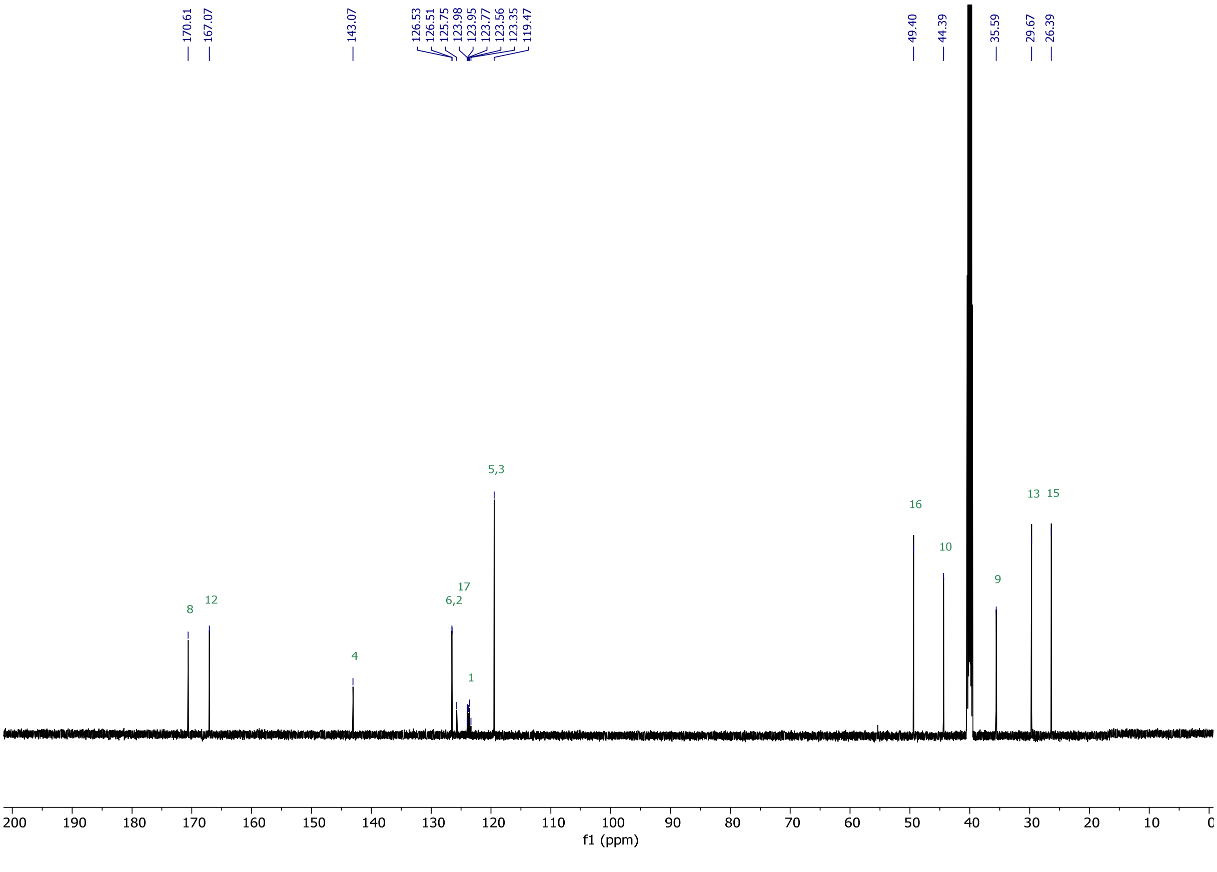

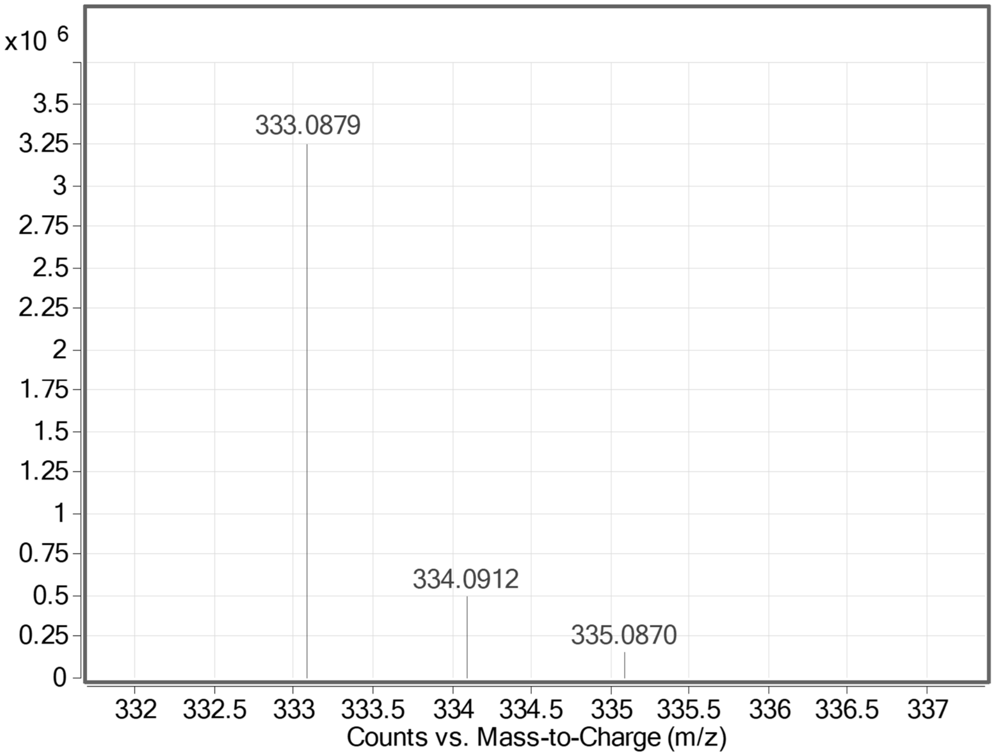
x10 6
3.5
3.25
3
2.75
2.5
2.25
2
1.75
1.5
1.25
1
0.75
0.5
0.25
0
333.0879
334.0912
335.0870
332
332.5
333
333.5
334
334.5
335
335.5
336
336.5
337
Counts vs. Mass-to-Charge (m/z)

3-(2-oxopiperidin-1-yl)-*N*-(4-(trifluoromethyl)phenyl)propanamide (6)

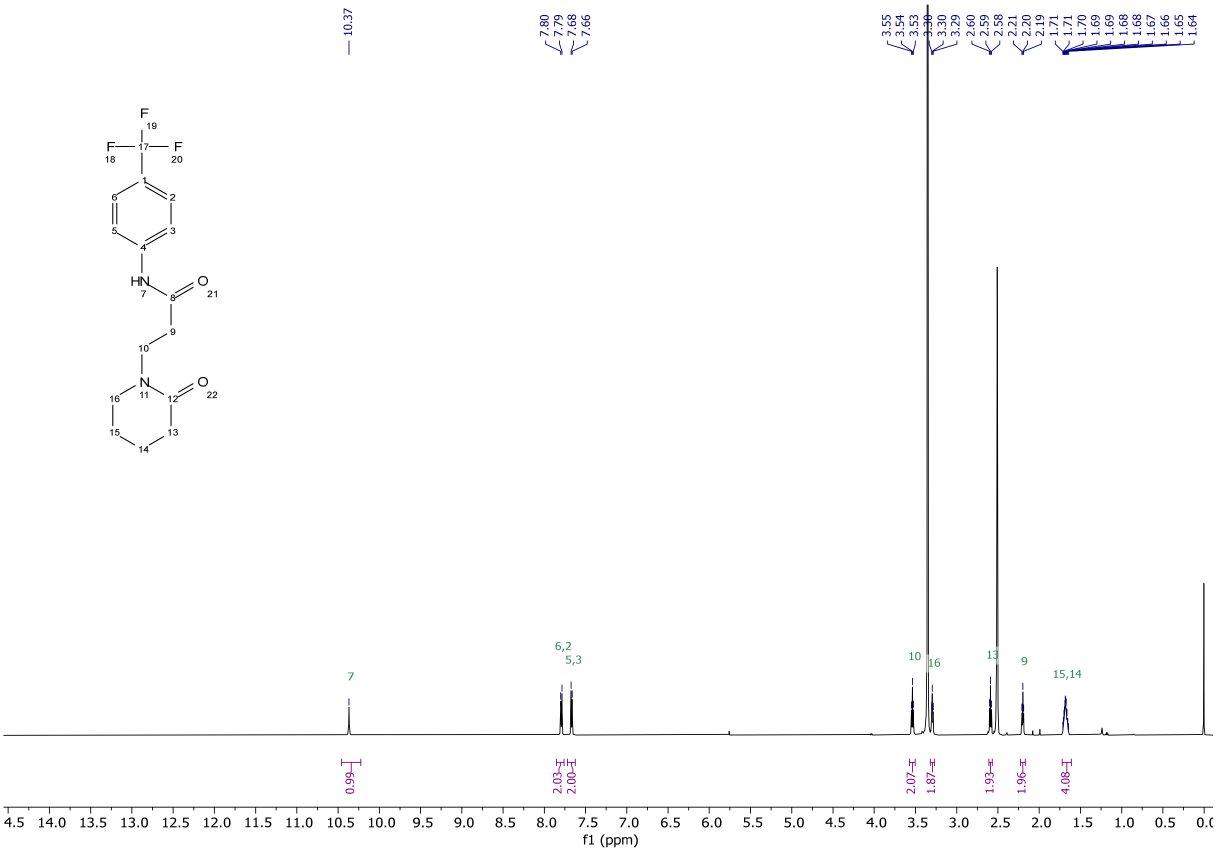

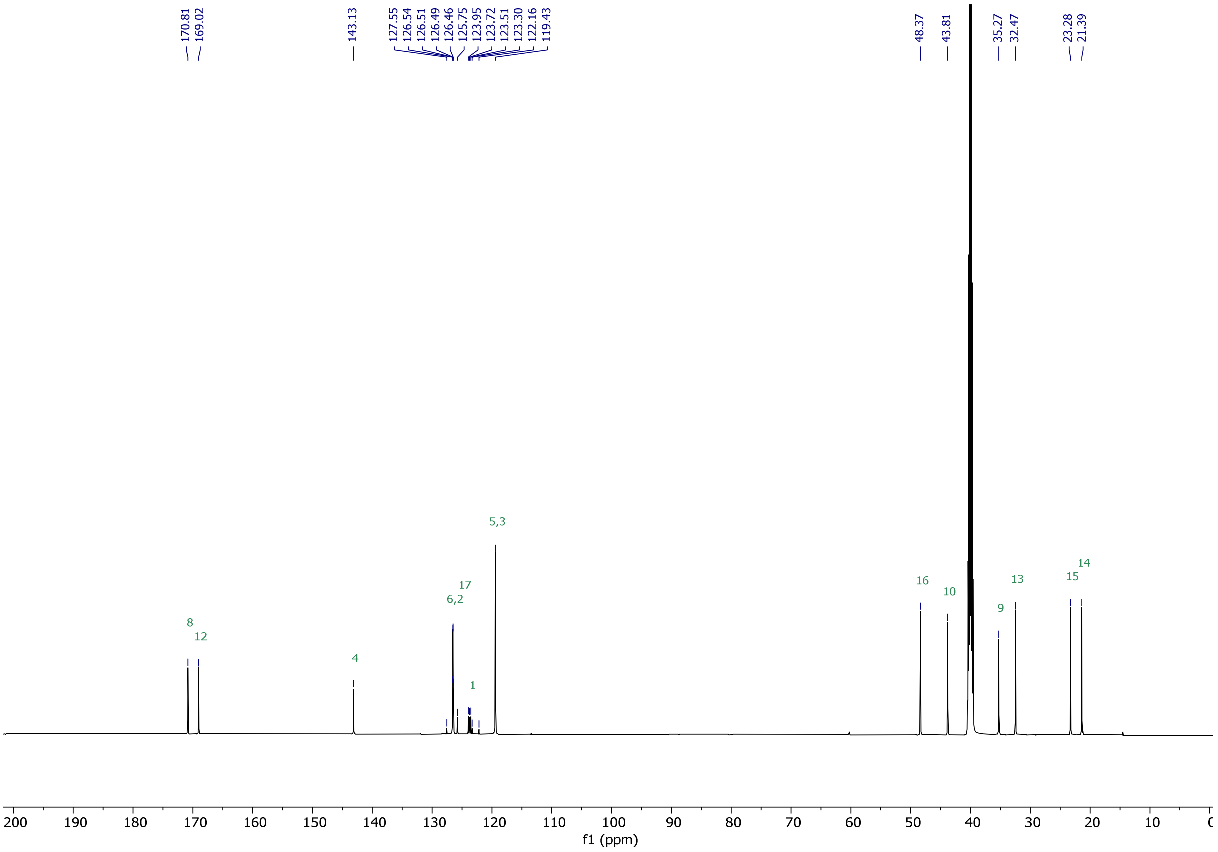

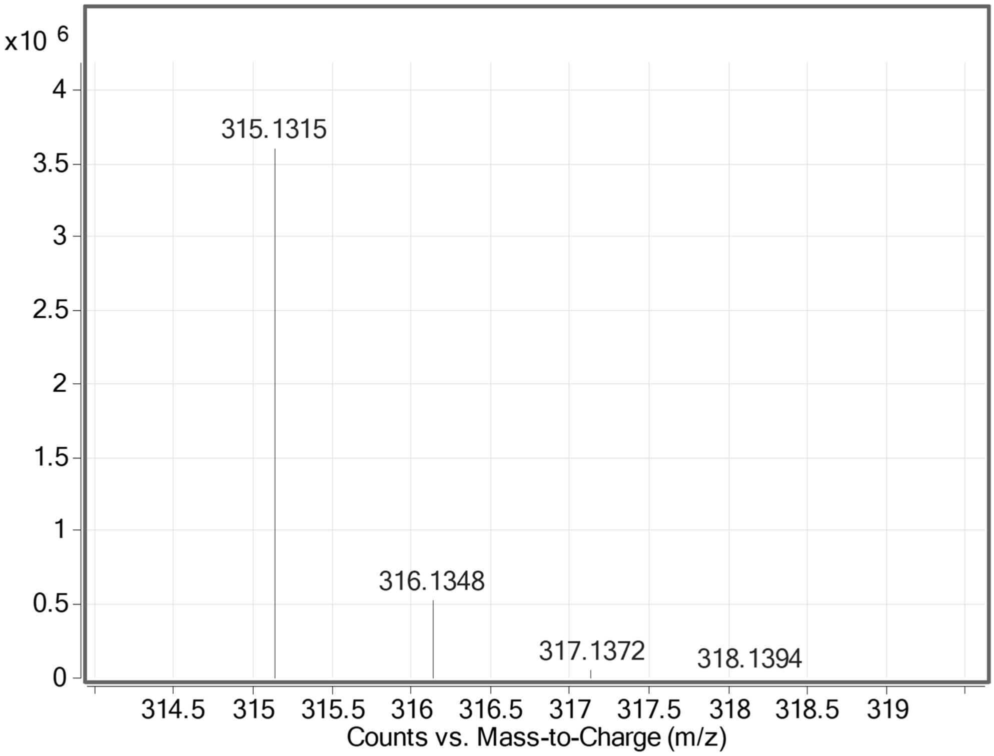

x10 6
4
3.5
3
2.5
2
1.5
1
0.5
0
315.1315
316.1348
317.1372
318.1394
314.5
315
315.5
316
316.5
317
317.5
318
318.5
319
Counts vs. Mass-to-Charge (m/z)

3-((2-methylphenyl)sulfonamido)-*N*-(4-(trifluoromethyl)phenyl)propanamide (7)

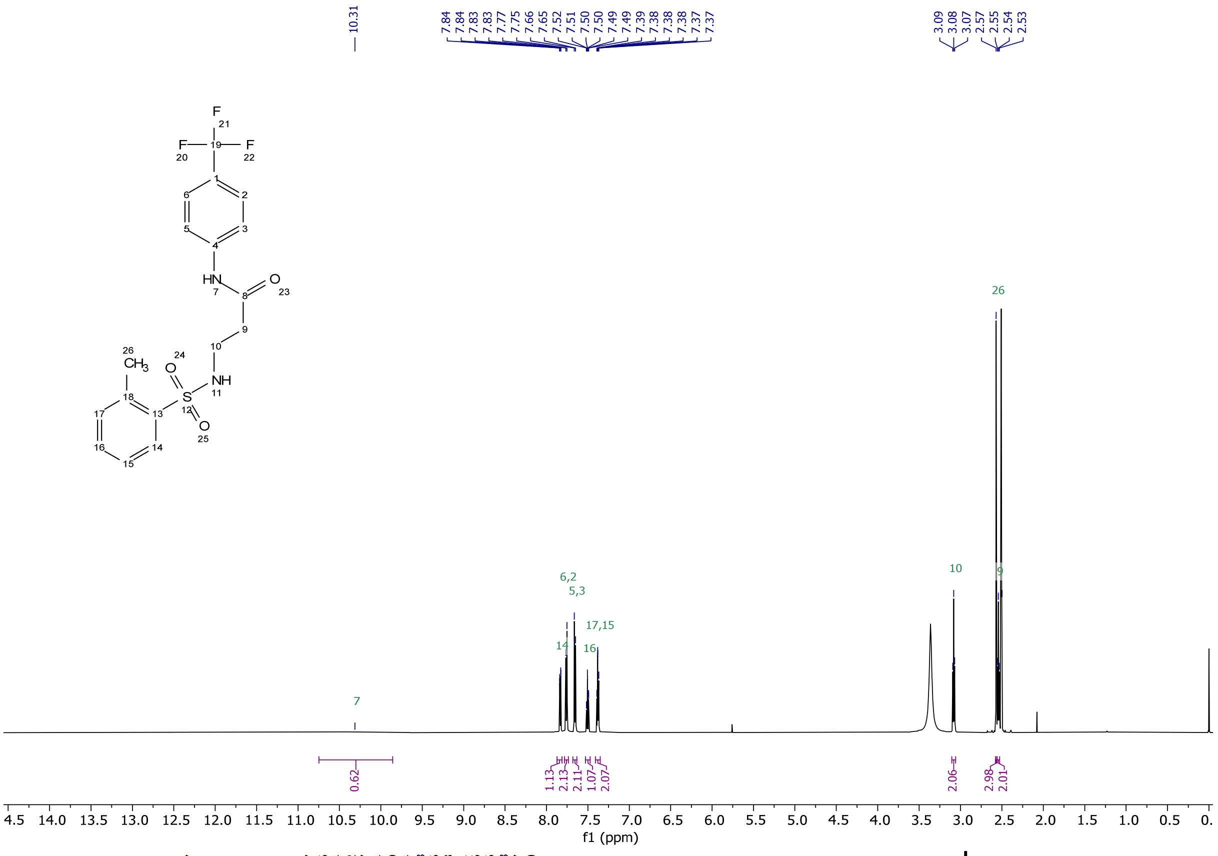

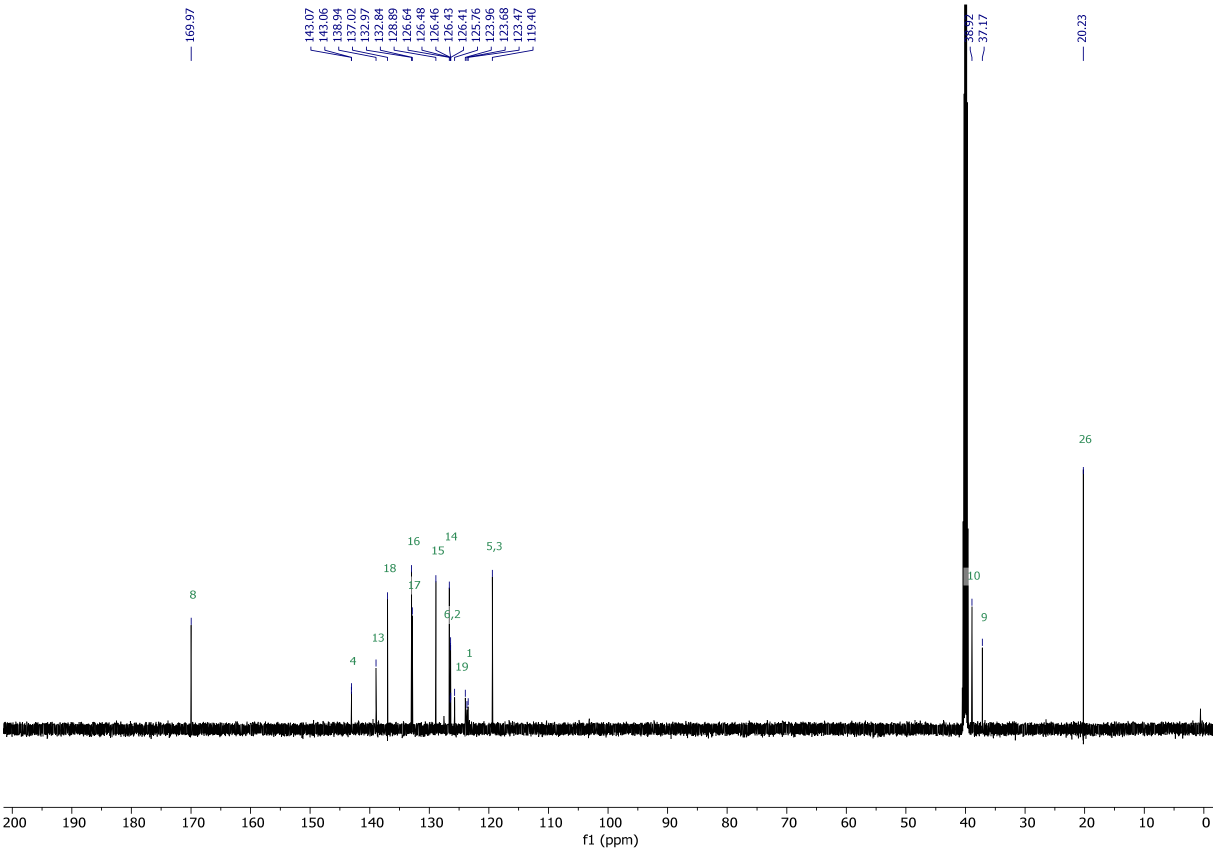

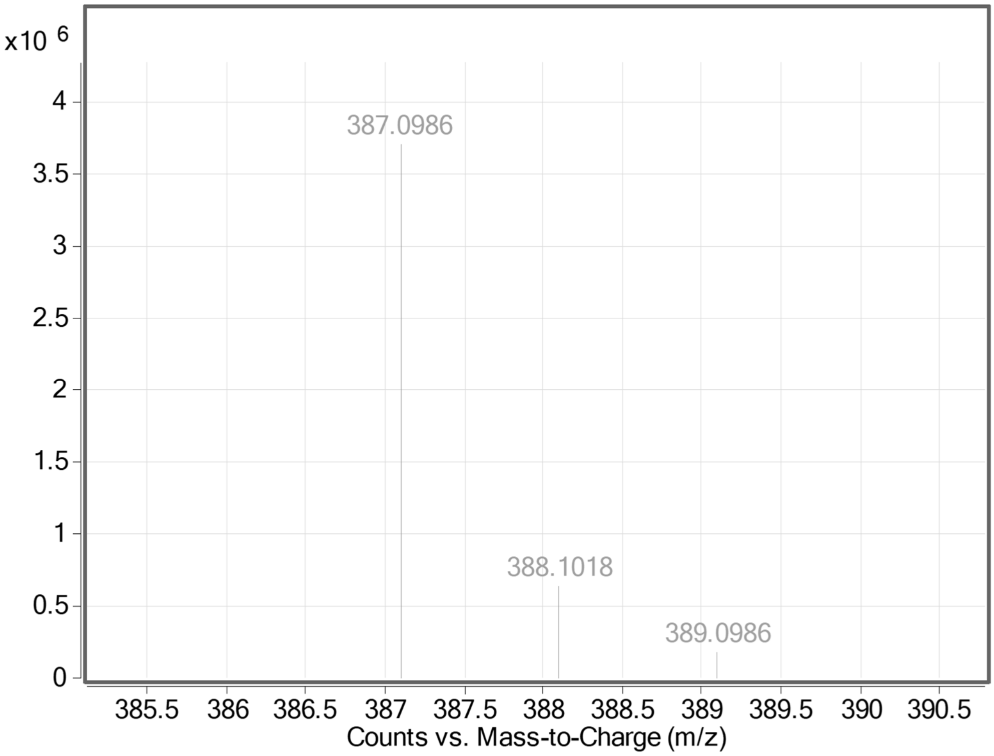

x10 6
4
3.5
3
2.5
2
1.5
1
0.5
0
387.0986
388.1018
389.0986
385.5
386
386.5
387
387.5
388
388.5
389
389.5
390
390.5
Counts vs. Mass-to-Charge (m/z)

3-((4-methylphenyl)sulfonamido)-*N*-(4-(trifluoromethyl)phenyl)propanamide (8)

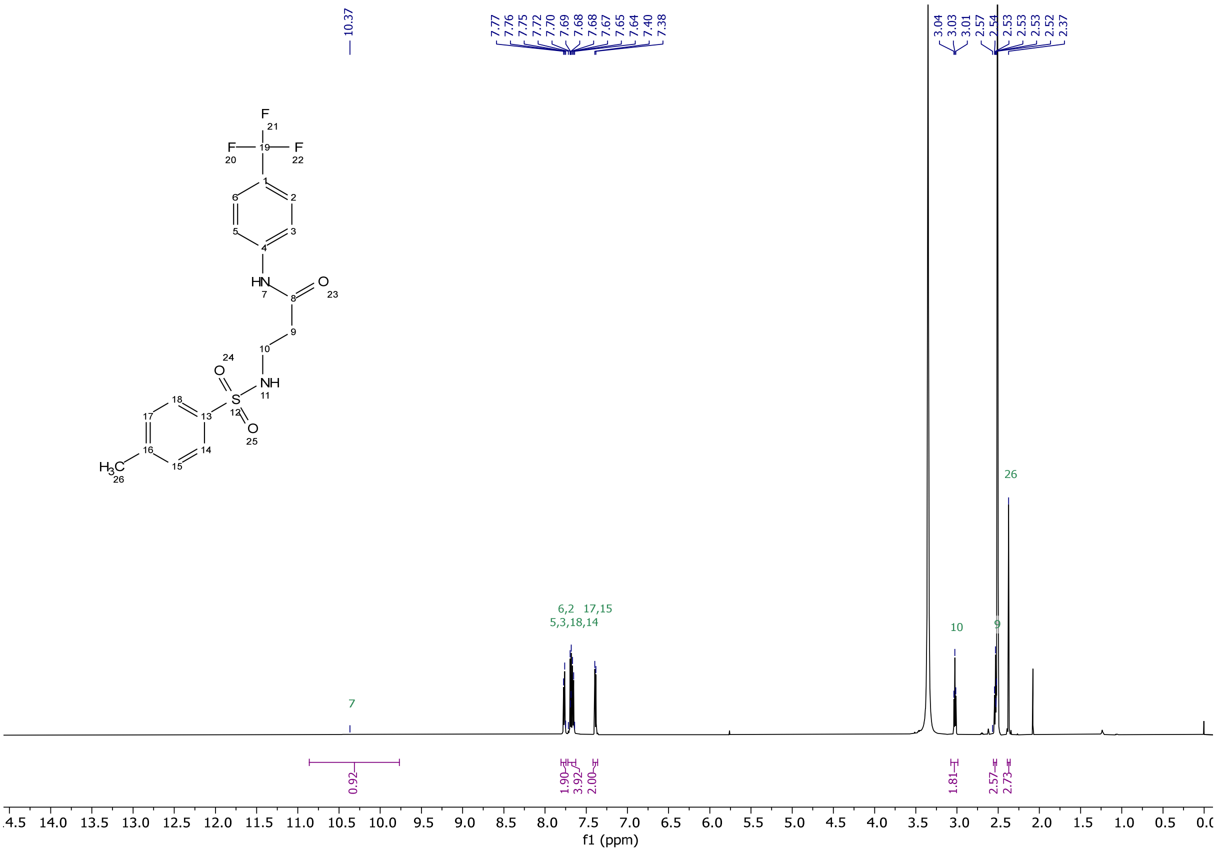

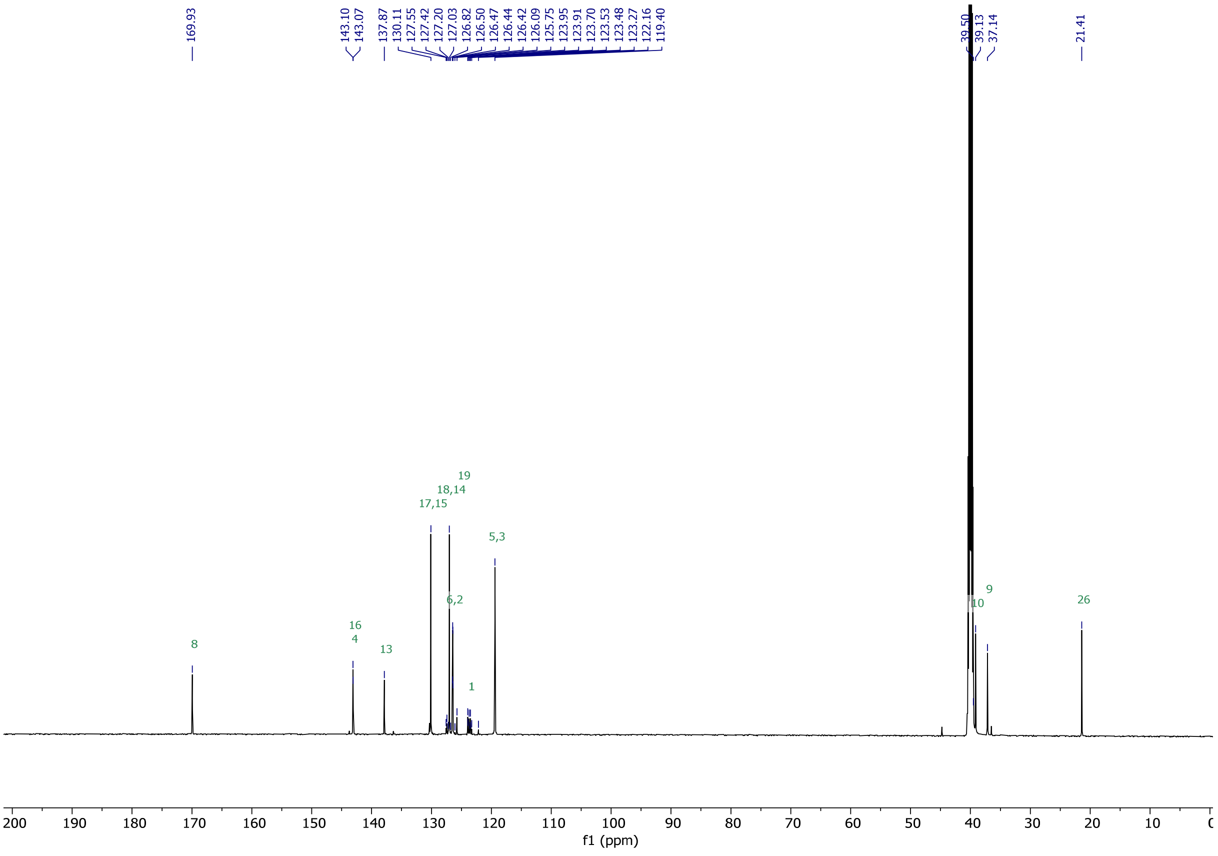

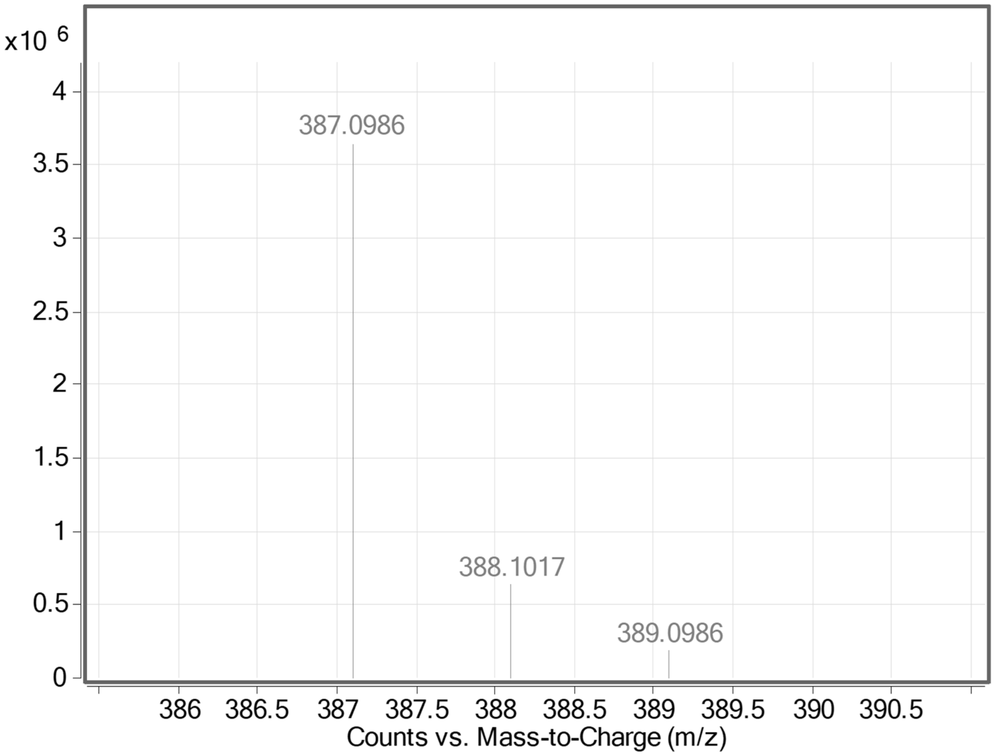

x10 6
4
3.5
3
2.5
2
1.5
1
0.5
0
387.0986
388.1017
389.0986
386
386.5
387
387.5
388
388.5
389
389.5
390
390.5
Counts vs. Mass-to-Charge (m/z)

2-(3-oxo-2,3-dihydro-4*H*-benzo[*b*][1,4]thiazin-4-yl)-*N*-(4-(trifluoromethyl)phenyl)acetamide (9)

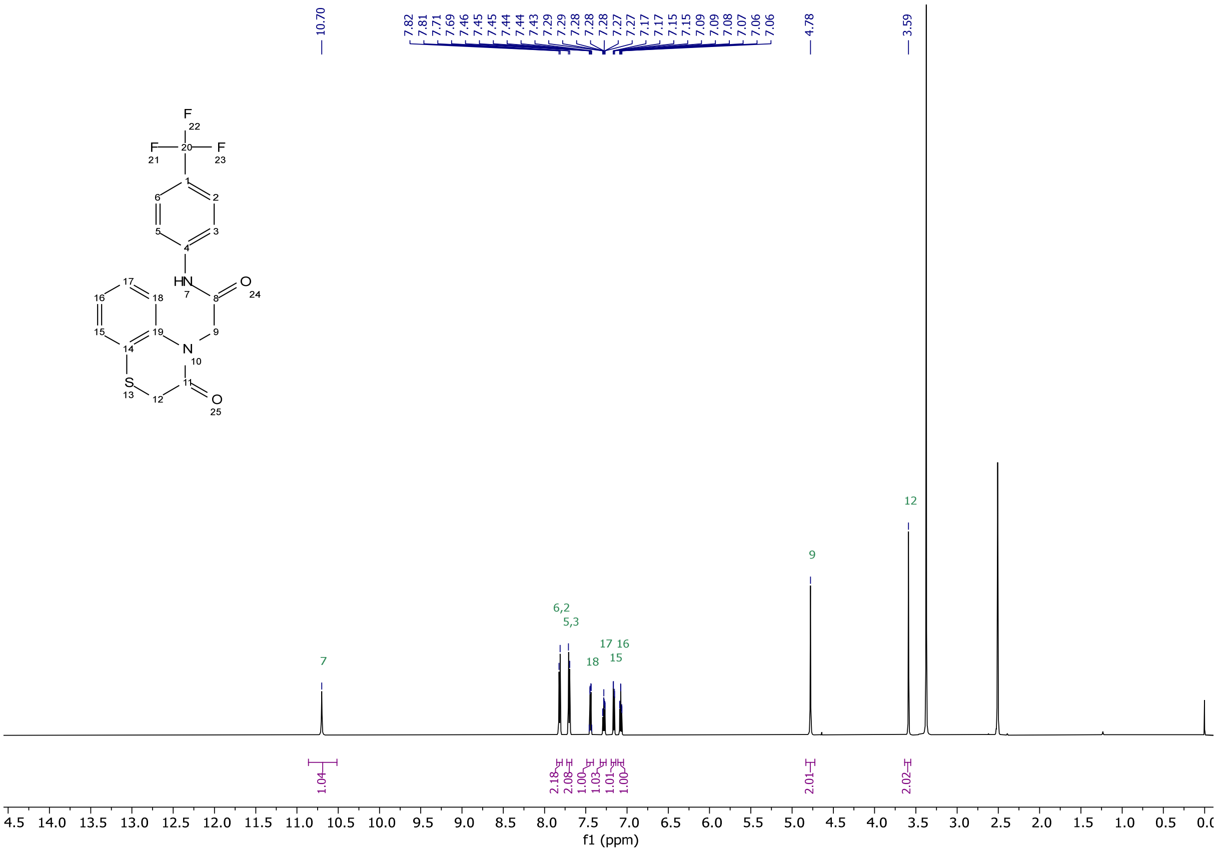

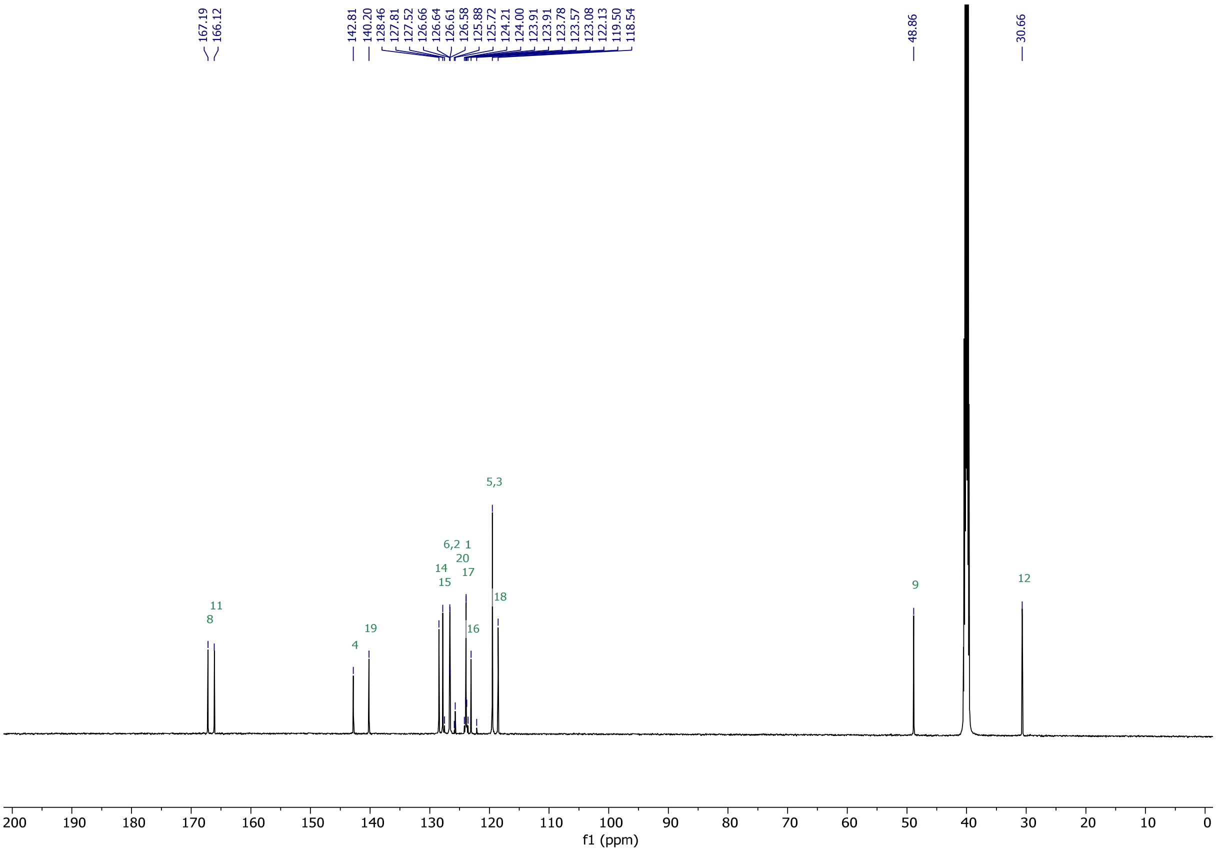

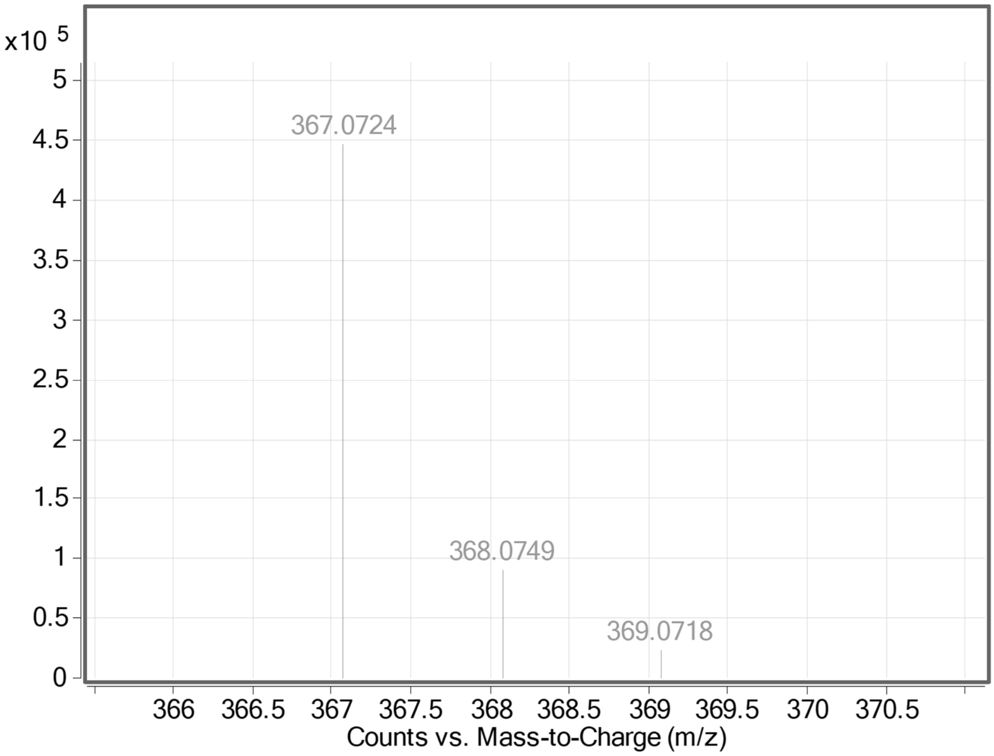
x10 5
5
4.5
4
3.5
3
2.5
2
1.5
1
0.5
0
367.0724
368.0749
369.0718
366
366.5
367
367.5
368
368.5
369
369.5
370
370.5
Counts vs. Mass-to-Charge (m/z)

3-(3-oxo-2,3-dihydro-4*H*-benzo[*b*][1,4]thiazin-4-yl)-*N*-phenylpropanamide (10)

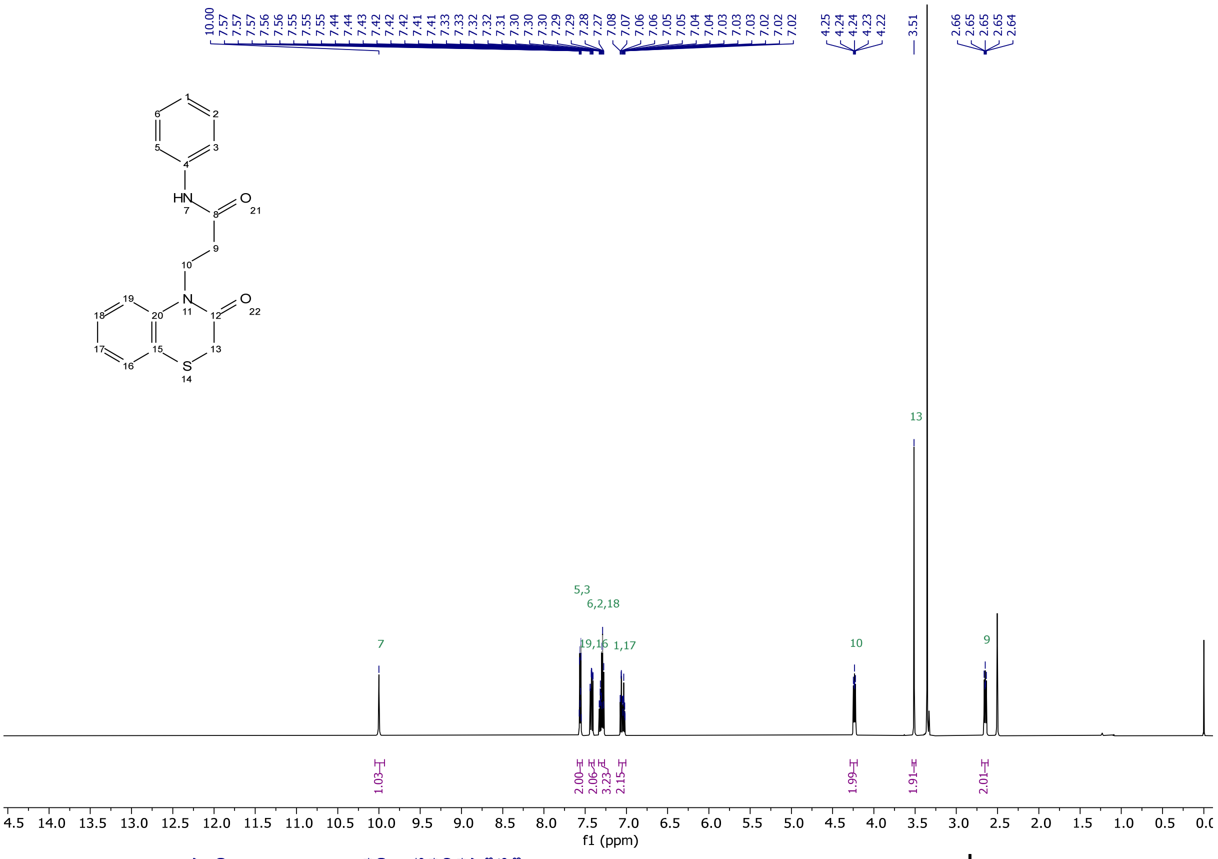

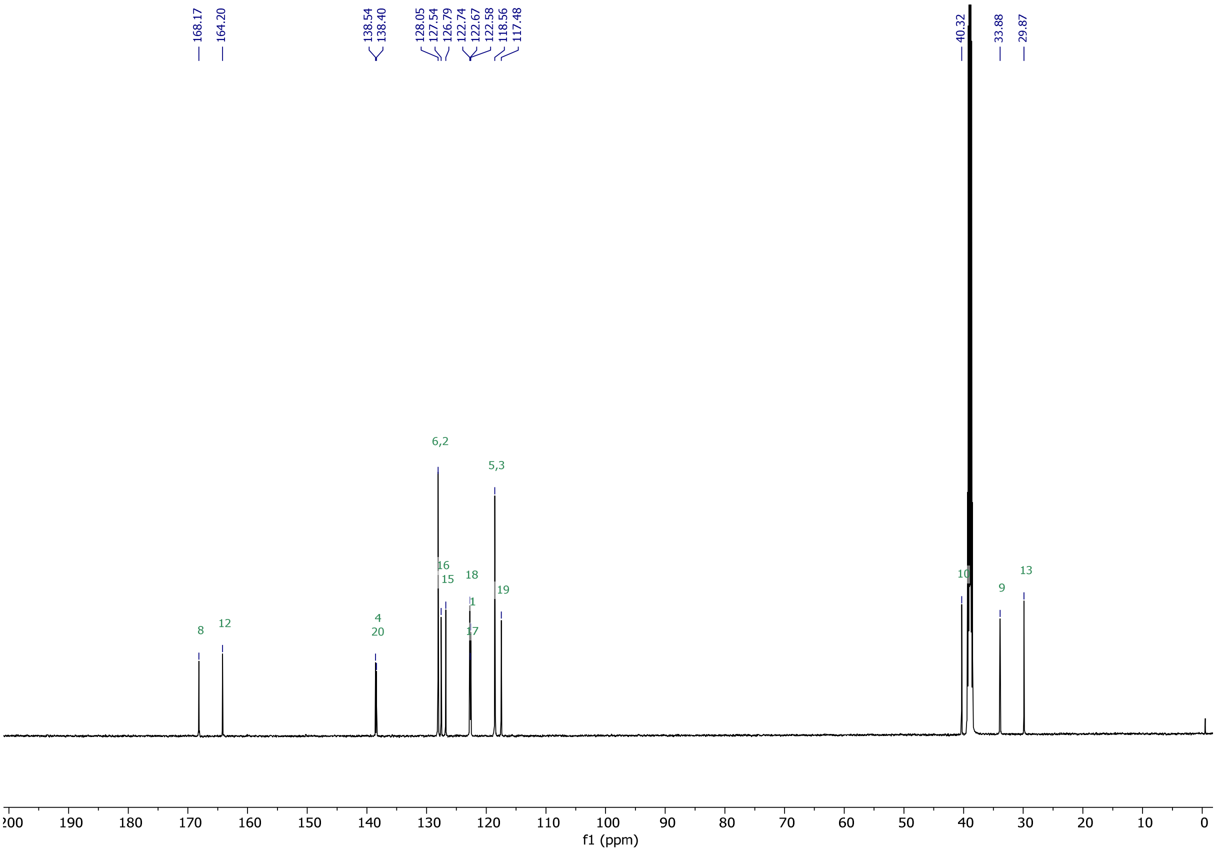

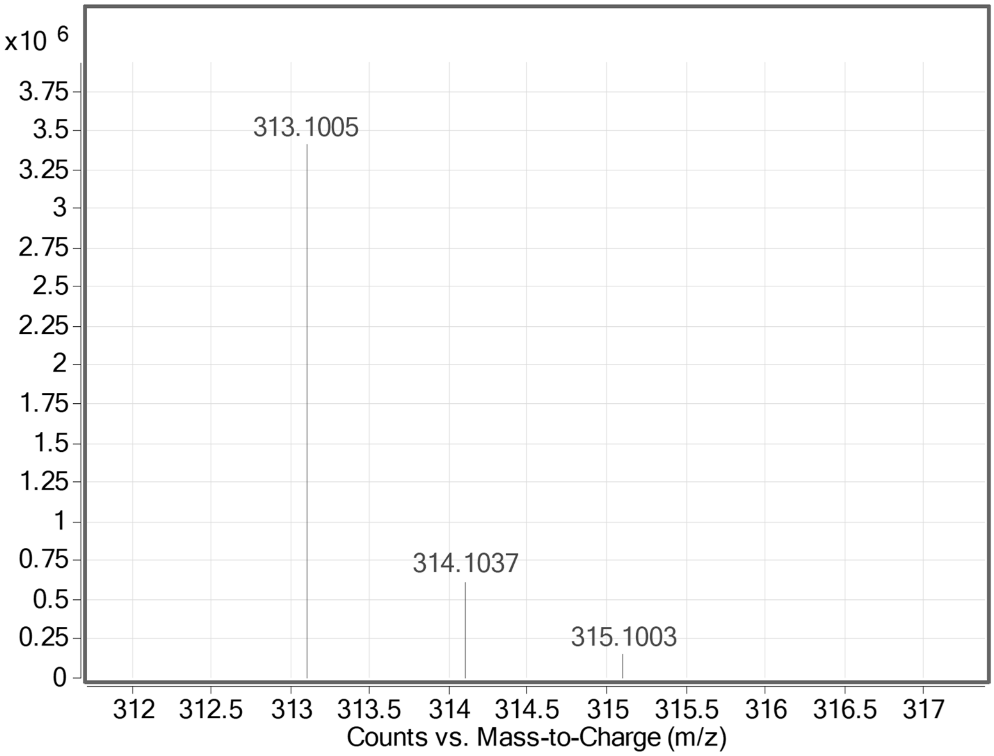
x10 6
3.75
3.5
3.25
3
2.75
2.5
2.25
2
1.75
1.5
1.25
1
0.75
0.5
0.25
0
313.1005
314.1037
315.1003
312
312.5
313
313.5
314
314.5
315
315.5
316
316.5
317
Counts vs. Mass-to-Charge (m/z)

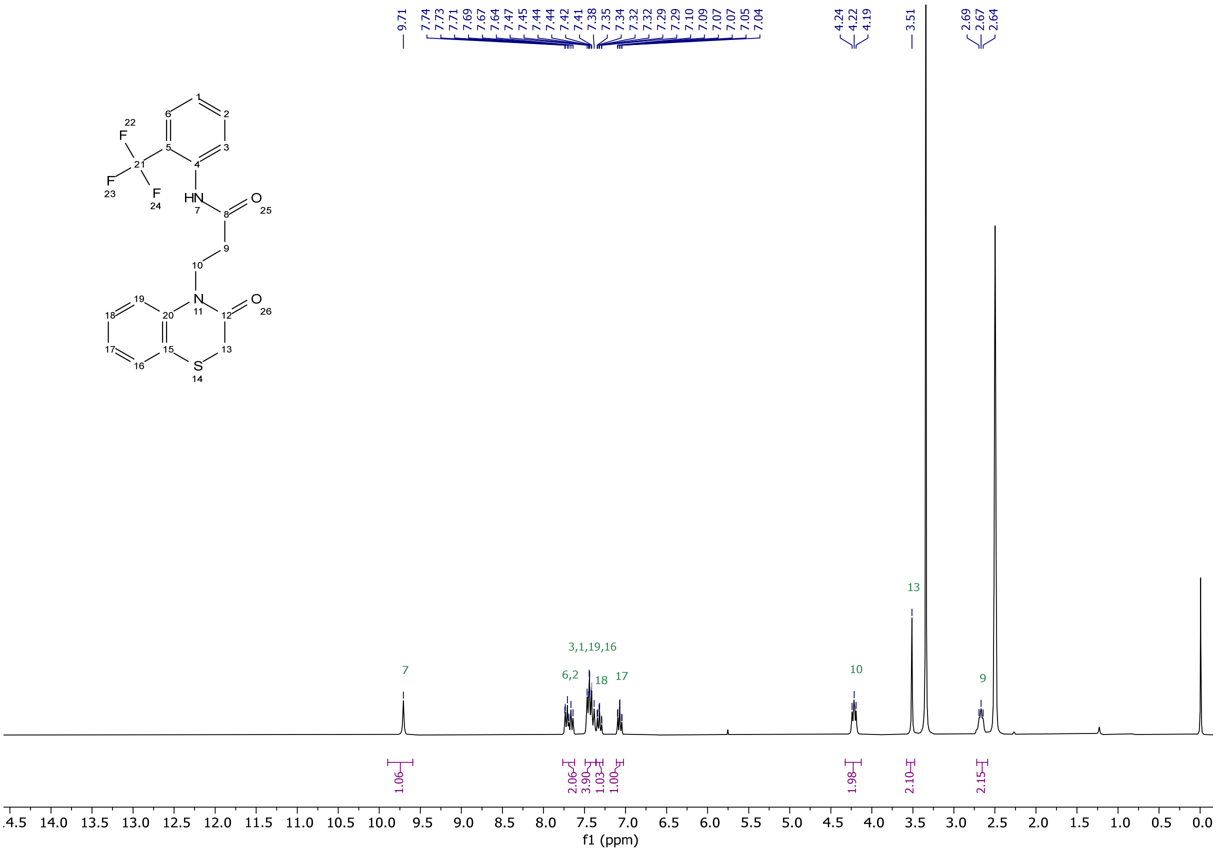

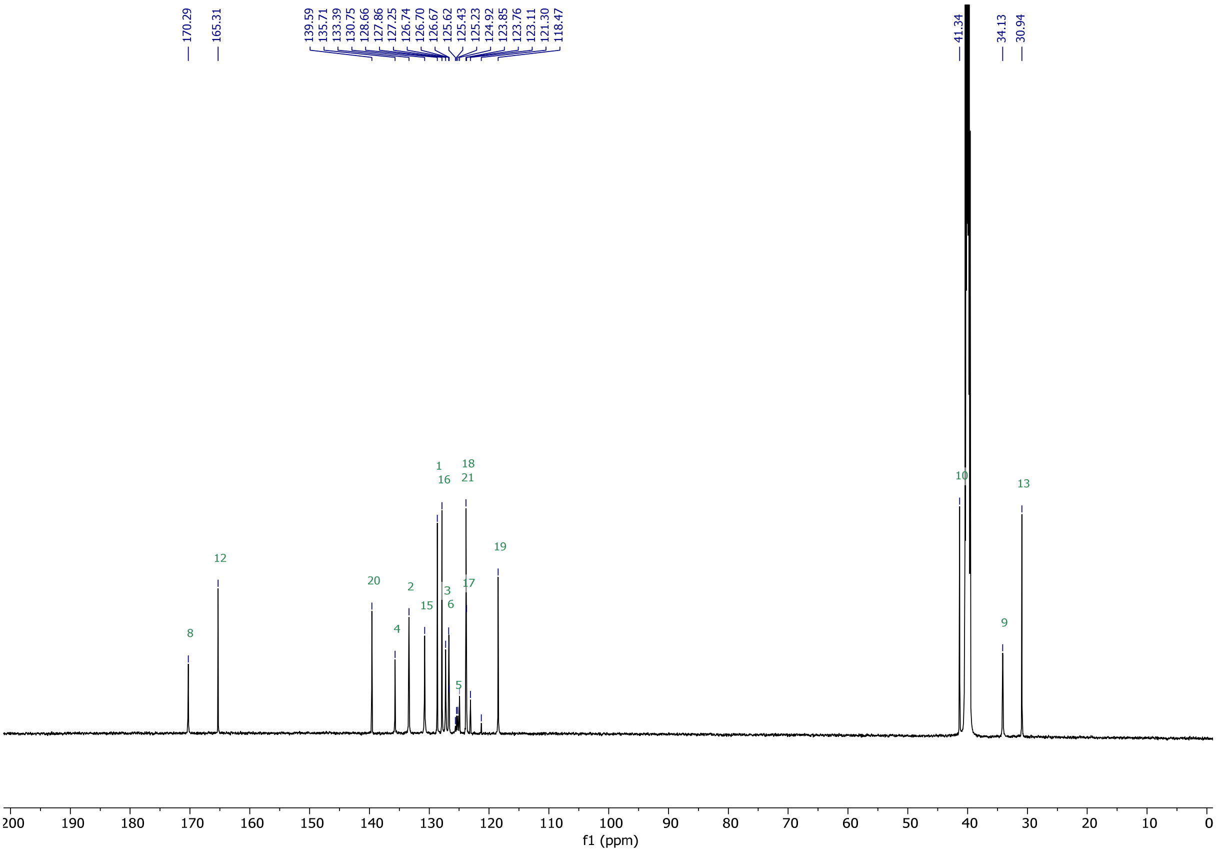

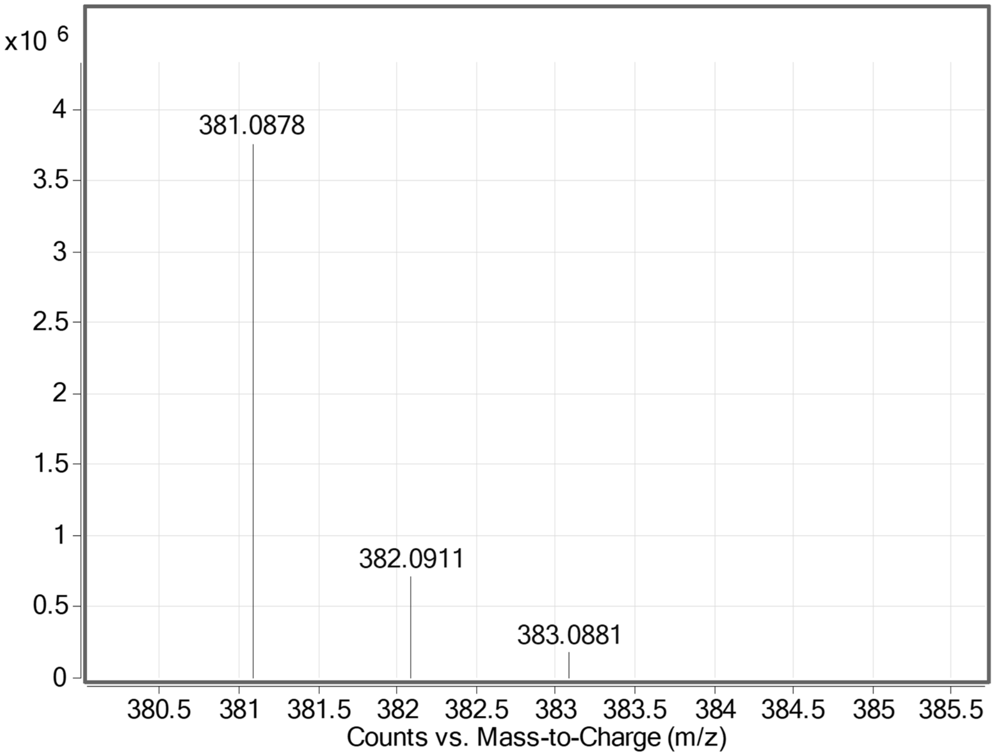
x10 6
4
3.5
3
2.5
2
1.5
1
0.5
0
381.0878
382.0911
383.0881
380.5
381
381.5
382
382.5
383
383.5
384
384.5
385
385.5
Counts vs. Mass-to-Charge (m/z)

3-(3-oxo-2,3-dihydro-4*H*-benzo[*b*][1,4]thiazin-4-yl)-*N*-(*o*-tolyl)propanamide (12)

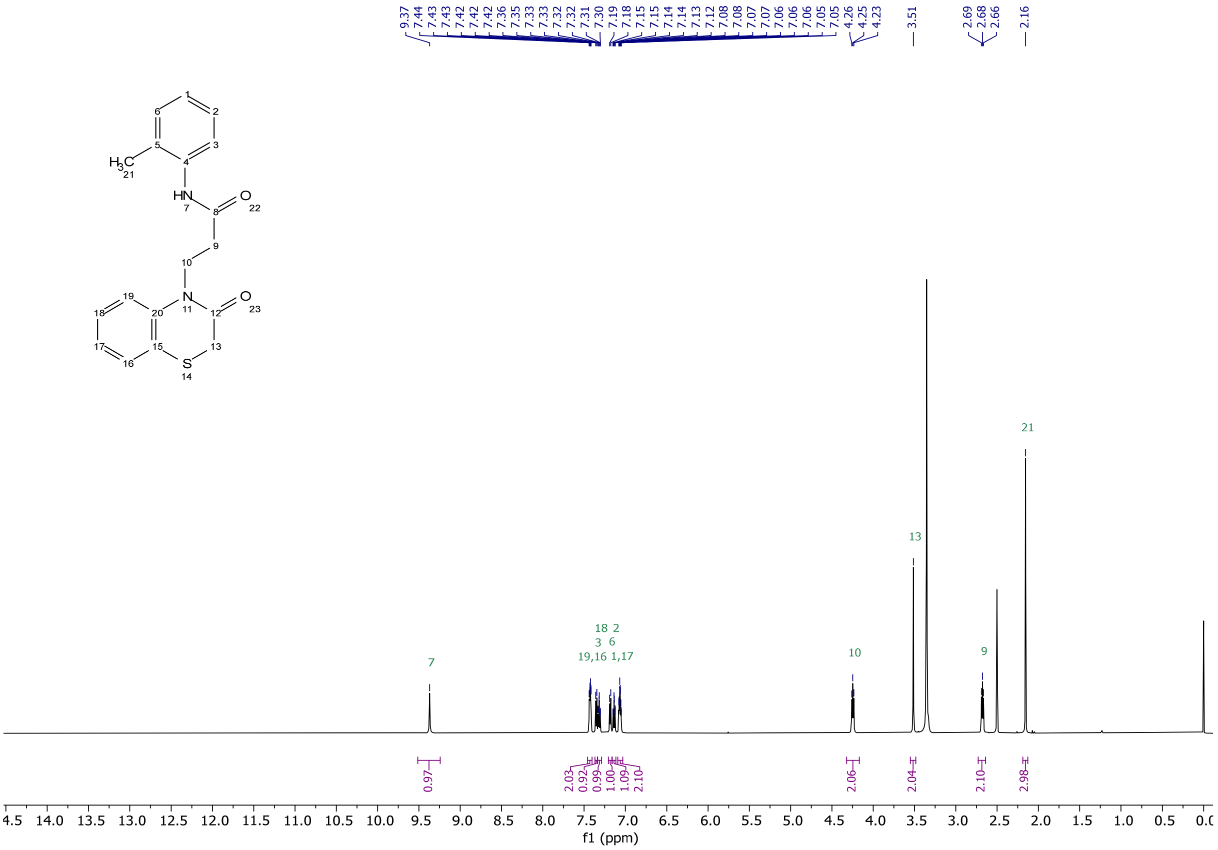

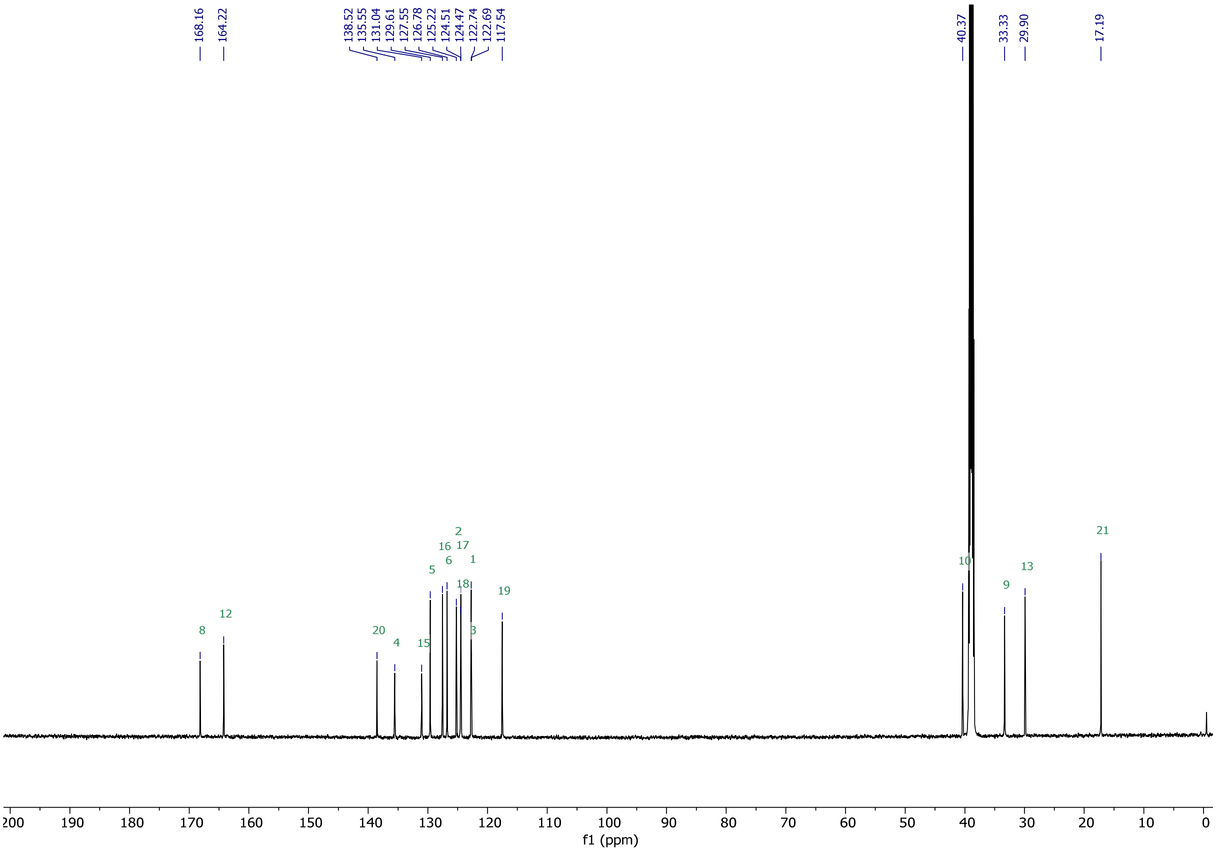

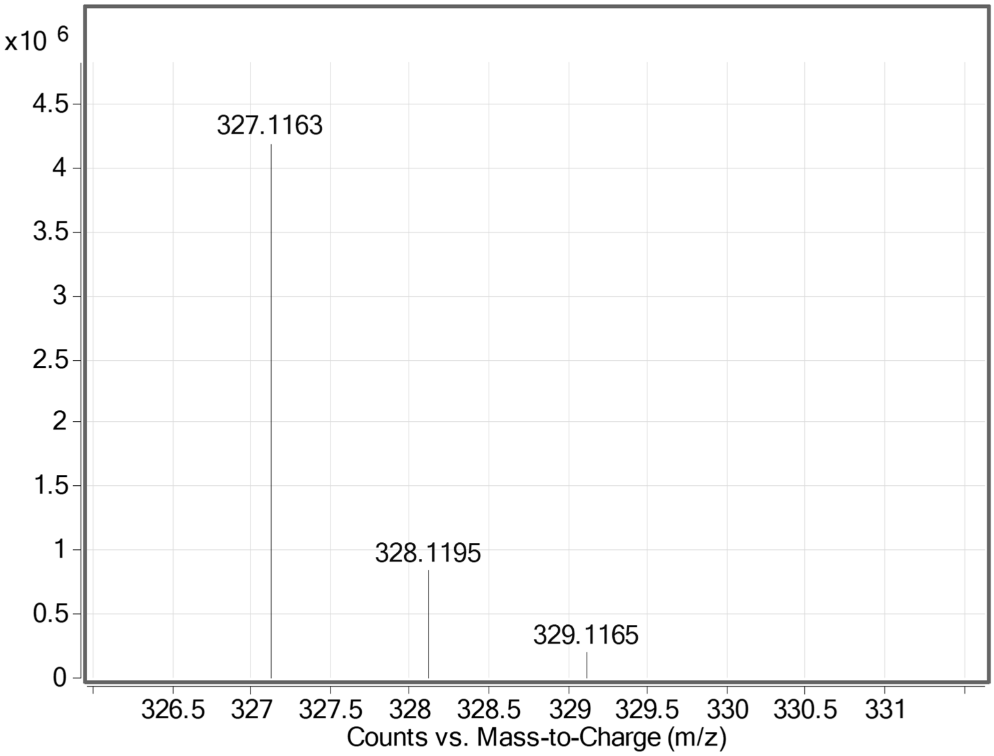

x10 6
4.5
4
3.5
3
2.5
2
1.5
1
0.5
0
327.1163
328.1195
329.1165
326.5
327
327.5
328
328.5
329
329.5
330
330.5
331
Counts vs. Mass-to-Charge (m/z)

3-(3-oxo-2,3-dihydro-4*H*-benzo[*b*][1,4]thiazin-4-yl)-*N*-(*m*-tolyl)propanamide (13)

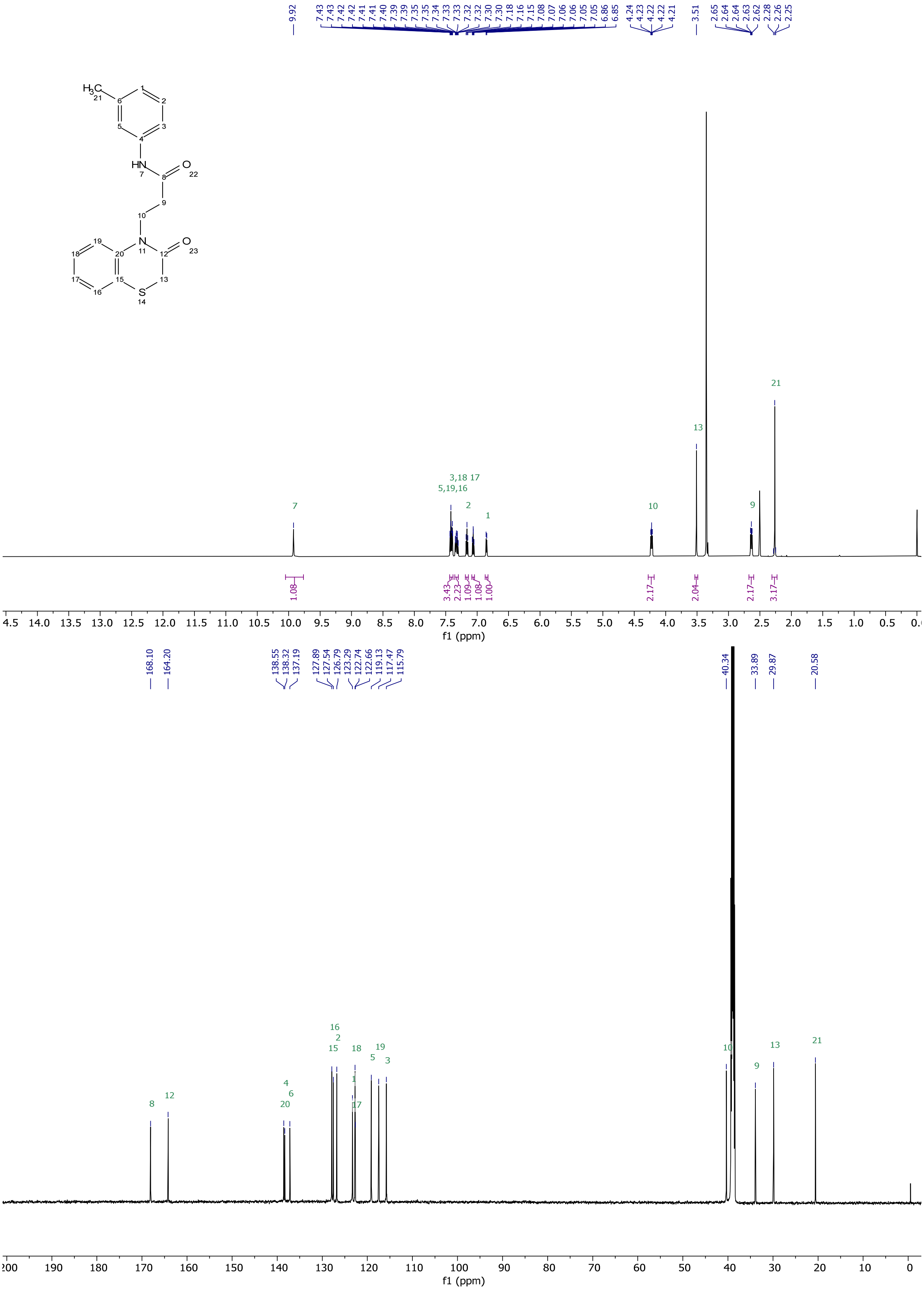

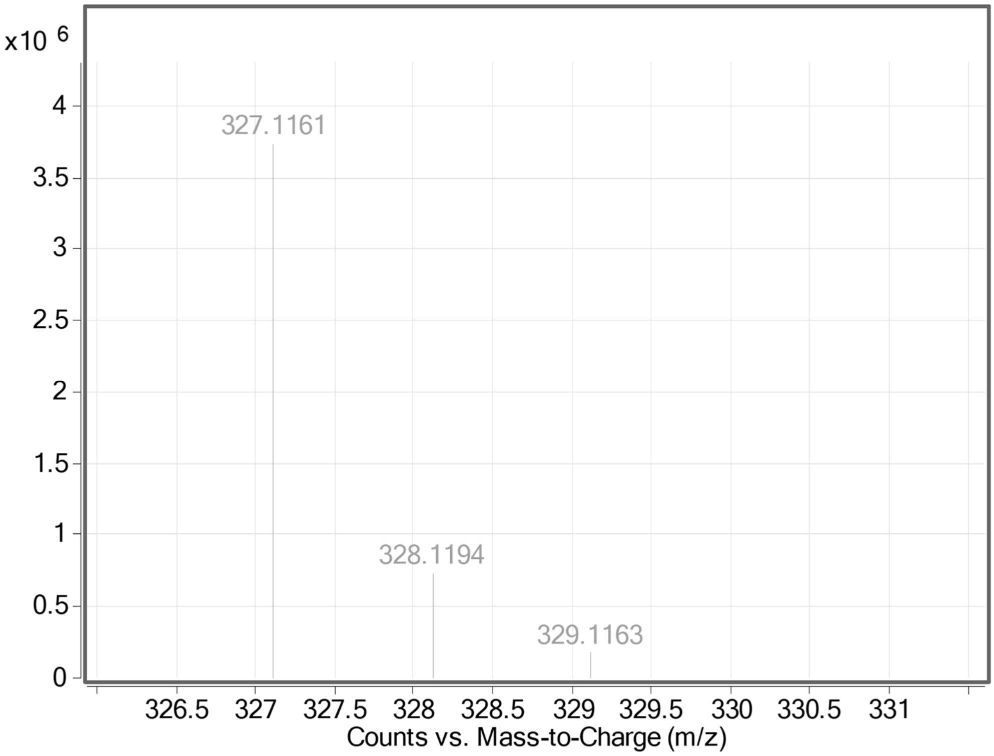

x10 6
4
3.5
3
2.5
2
1.5
1
0.5
0
327.1161
328.1194
329.1163
326.5
327
327.5
328
328.5
329
329.5
330
330.5
331
Counts vs. Mass-to-Charge (m/z)

3-(3-oxo-2,3-dihydro-4*H*-benzo[*b*][1,4]thiazin-4-yl)-*N*-(*p*-tolyl)propanamide (14)

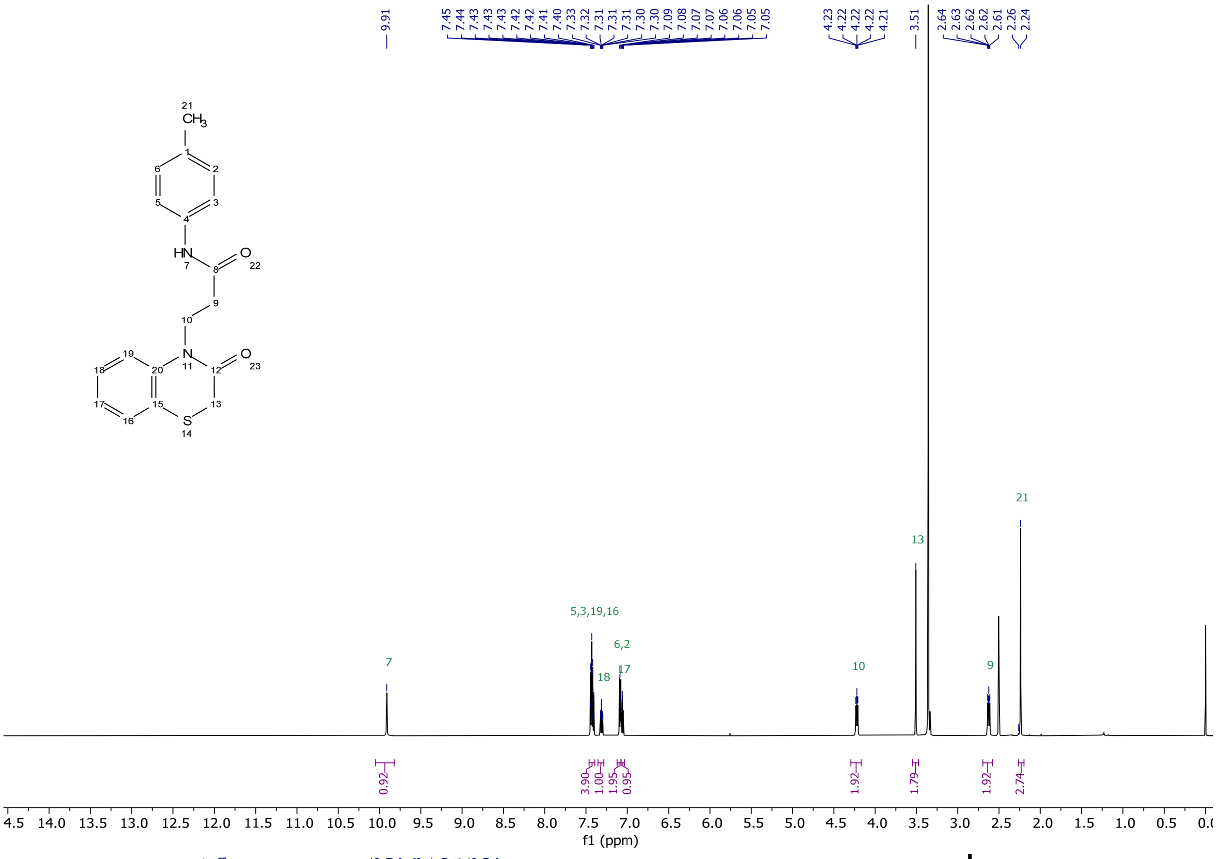

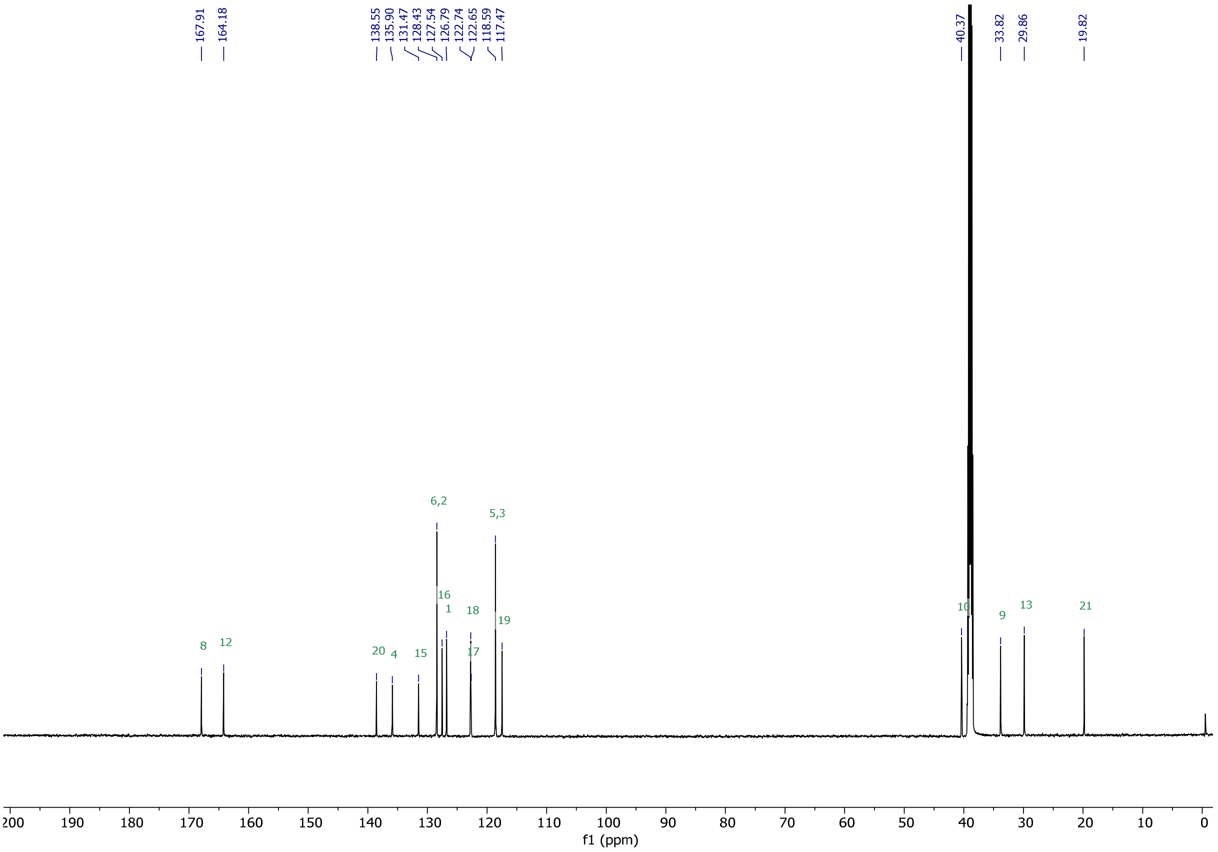

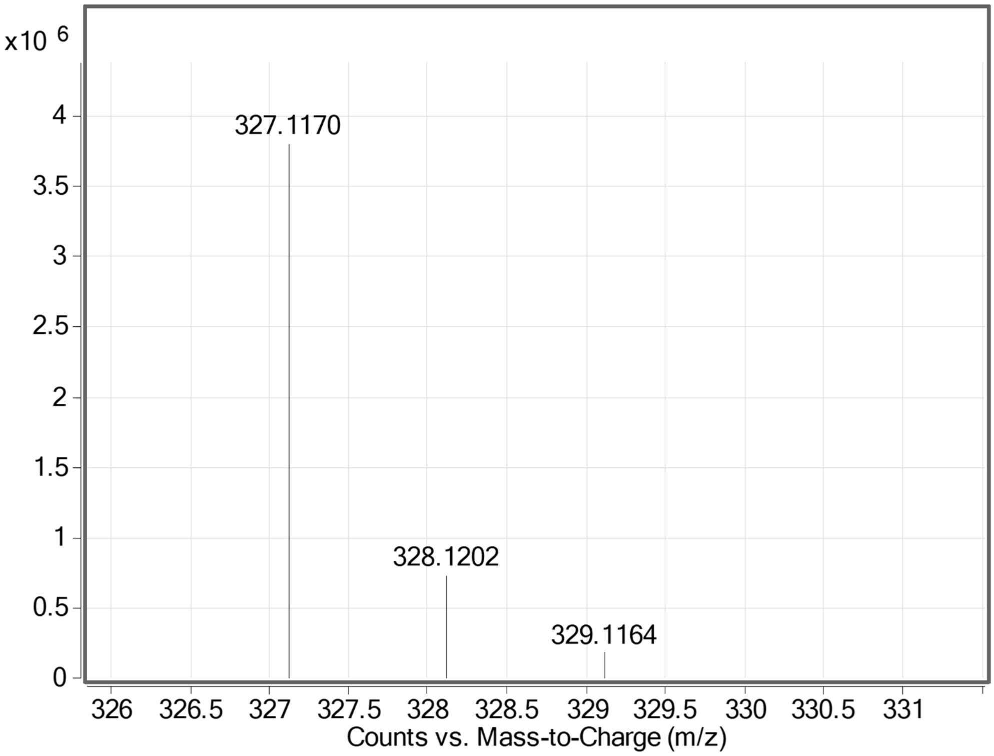

x10 6
4
3.5
3
2.5
2
1.5
1
0.5
0
327.1170
328.1202
329.1164
326
326.5
327
327.5
328
328.5
329
329.5
330
330.5
331
Counts vs. Mass-to-Charge (m/z)

***N*-(2-chlorophenyl)-3-(3-oxo-2,3-dihydro-4*H*-benzo[*b*][1,4]thiazin-4-yl)propanamide (15)**

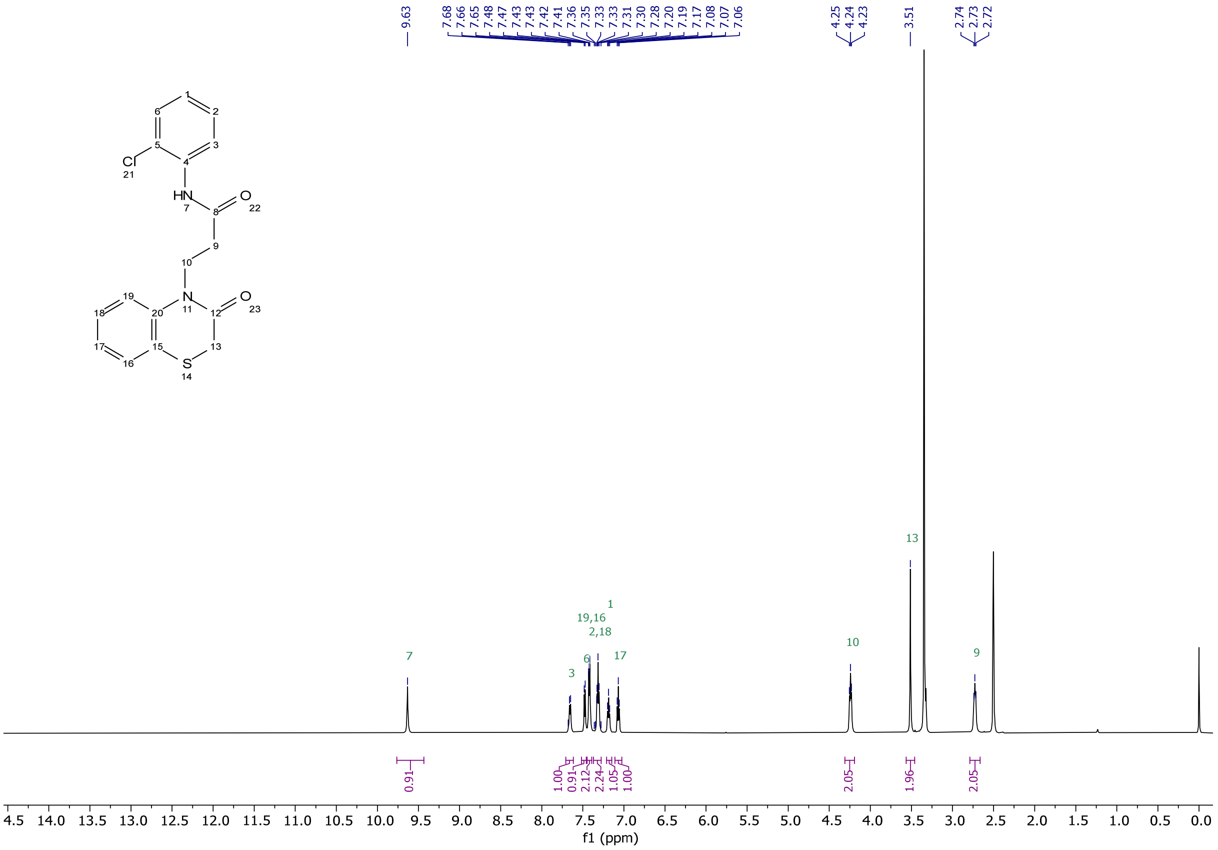

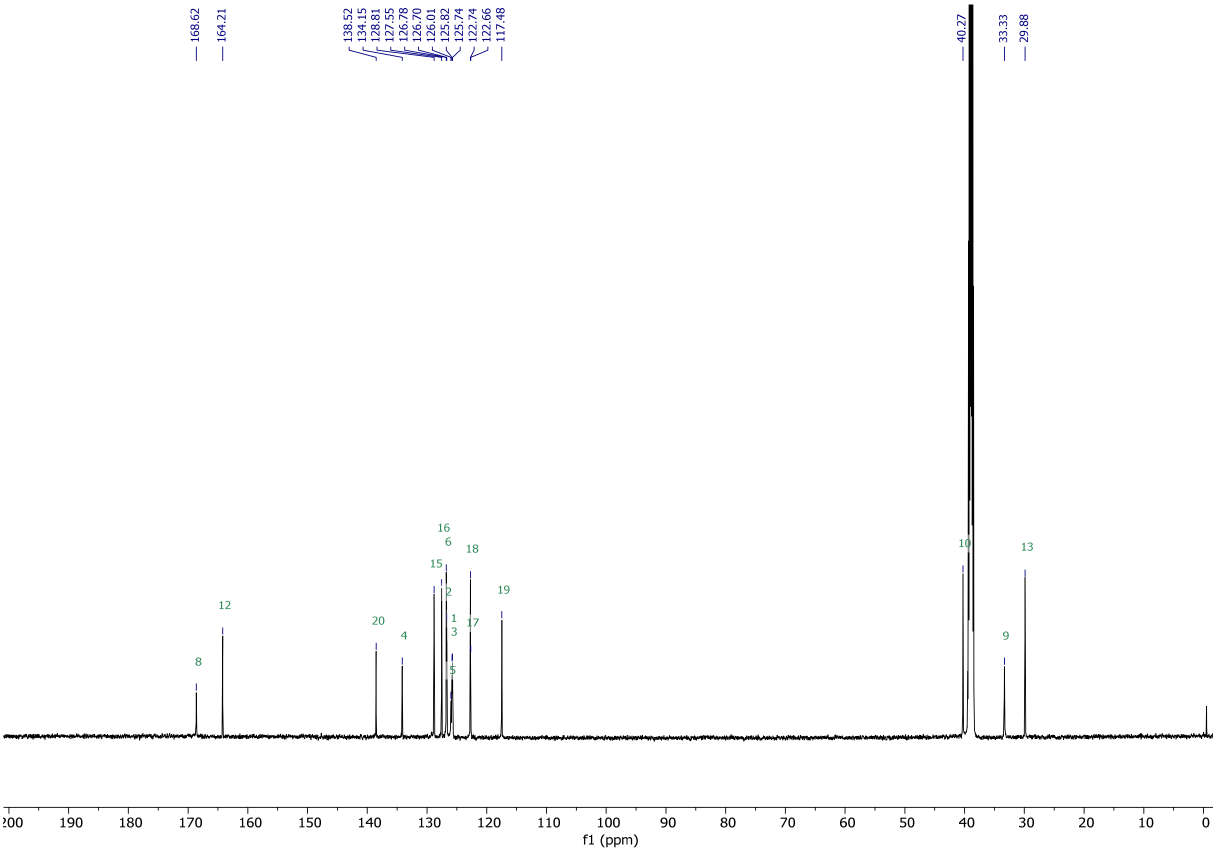

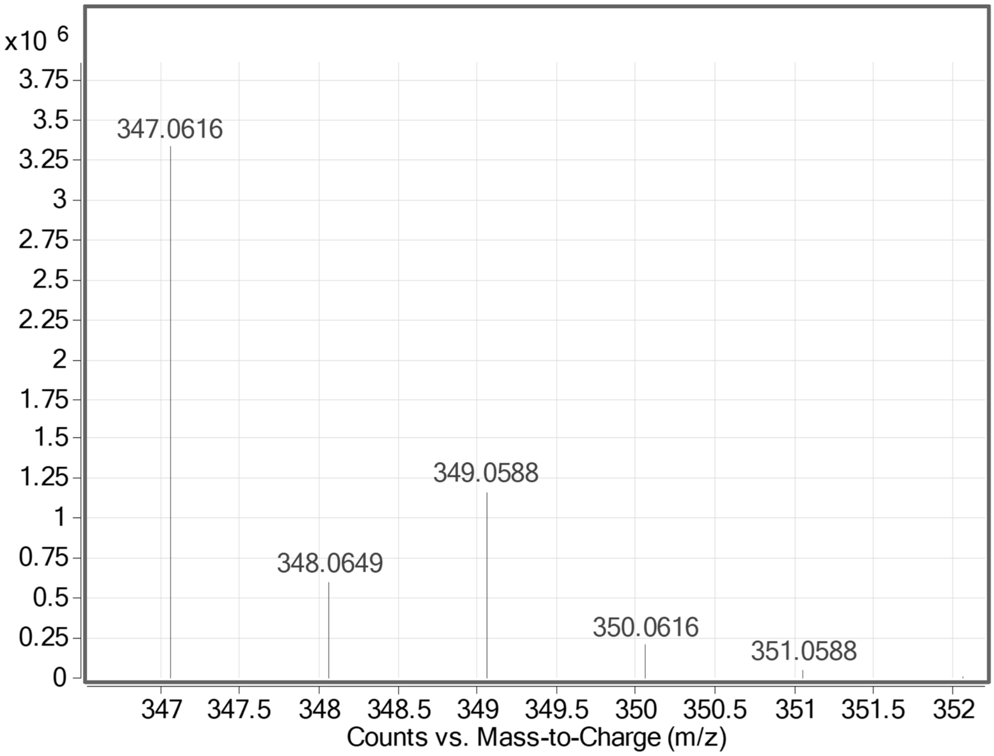
x10 6
3.75
3.5
3.25
3
2.75
2.5
2.25
2
1.75
1.5
1.25
1
0.75
0.5
0.25
0
347.0616
348.0649
349.0588
350.0616
351.0588
347
347.5
348
348.5
349
349.5
350
350.5
351
351.5
352
Counts vs. Mass-to-Charge (m/z)

N-(3-chlorophenyl)-3-(3-oxo-2,3-dihydro-4*H*-benzo[*b*][1,4]thiazin-4-yl)propanamide (16)

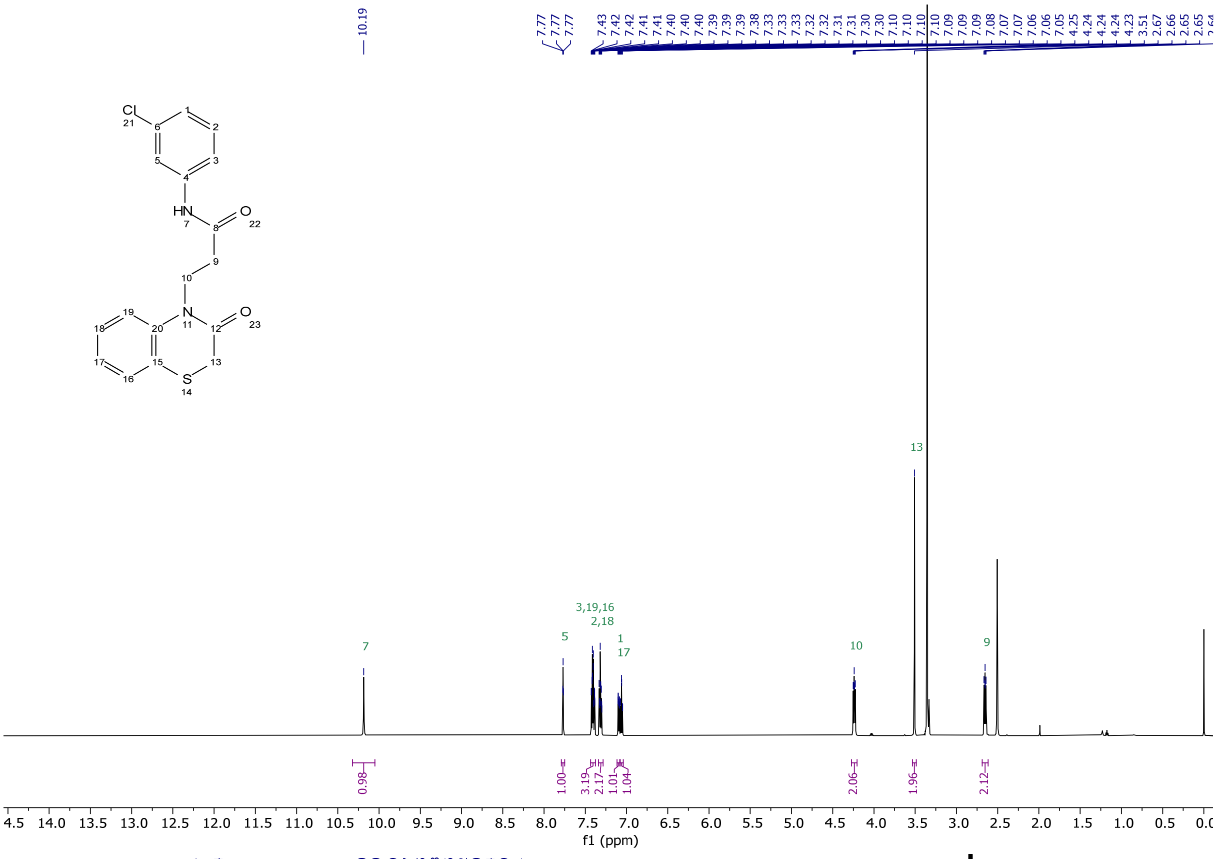

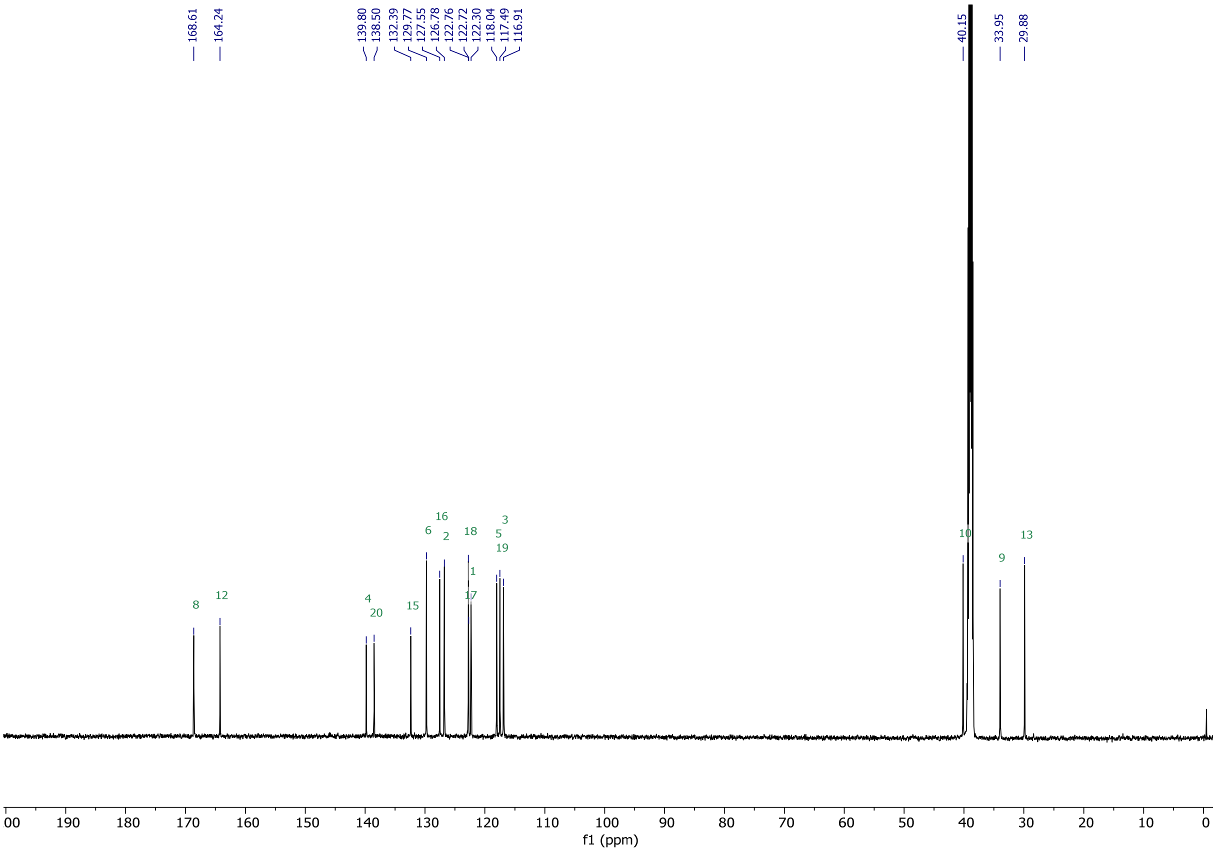

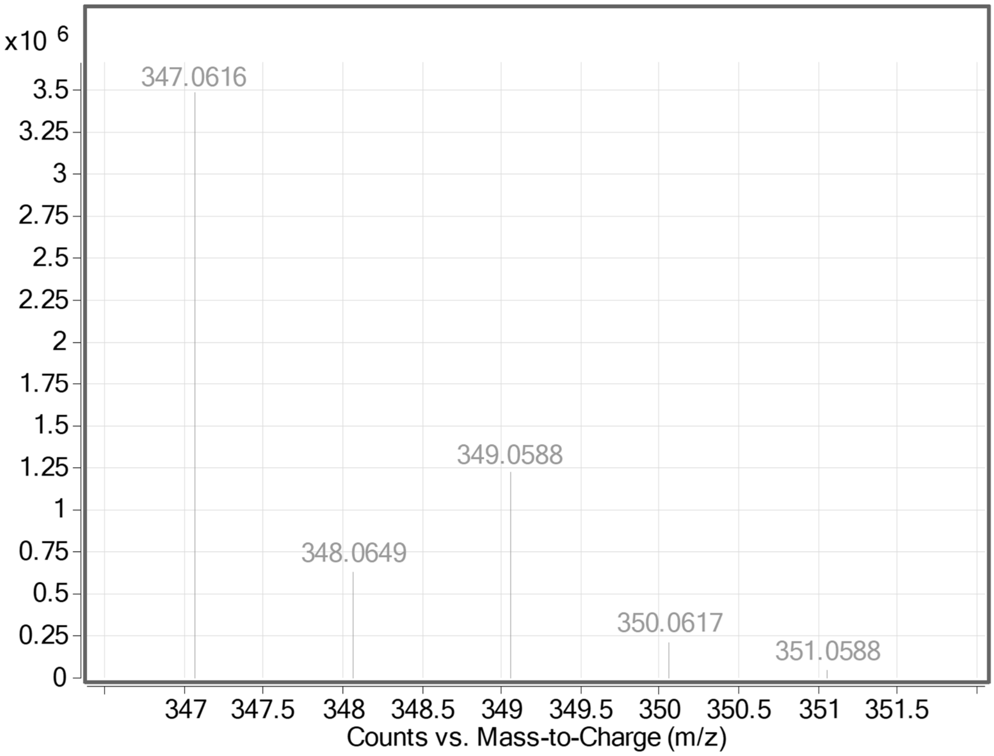
x10 6
3.5
3.25
3
2.75
2.5
2.25
2
1.75
1.5
1.25
1
0.75
0.5
0.25
0
347.0616
348.0649
349.0588
350.0617
351.0588
347
347.5
348
348.5
349
349.5
350
350.5
351
351.5
Counts vs. Mass-to-Charge (m/z)

***N*-(4-chlorophenyl)-3-(3-oxo-2,3-dihydro-4*H*-benzo[*b*][1,4]thiazin-4-yl)propanamide (17)**

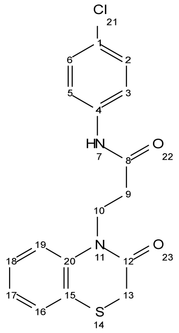

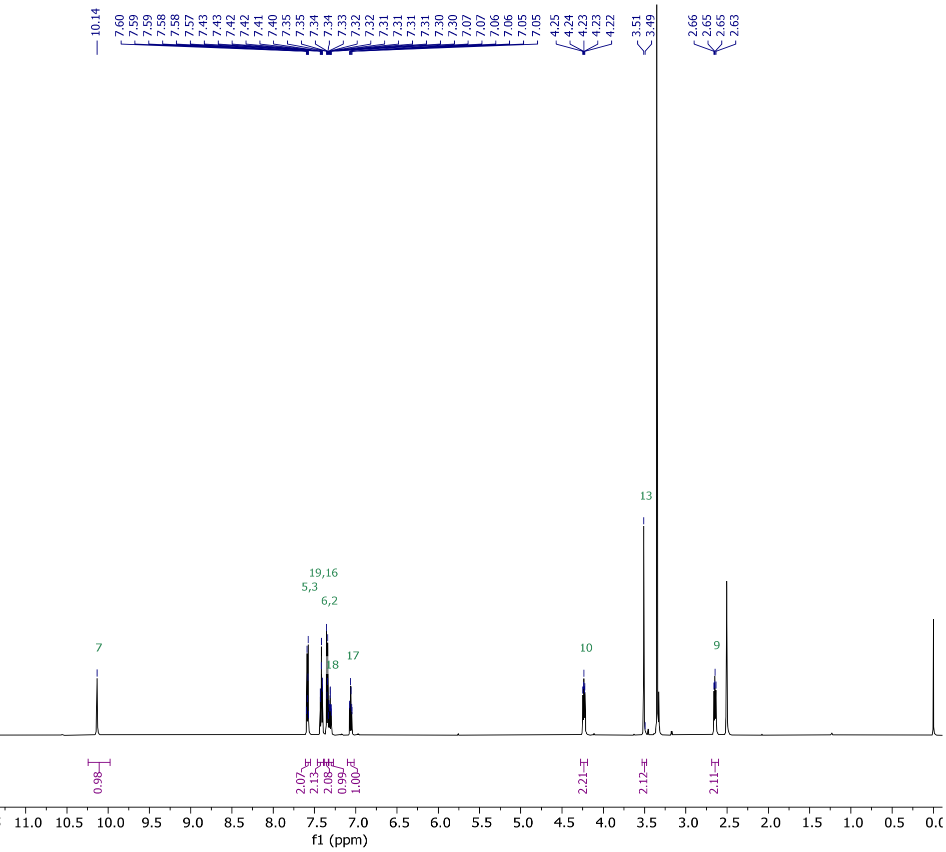

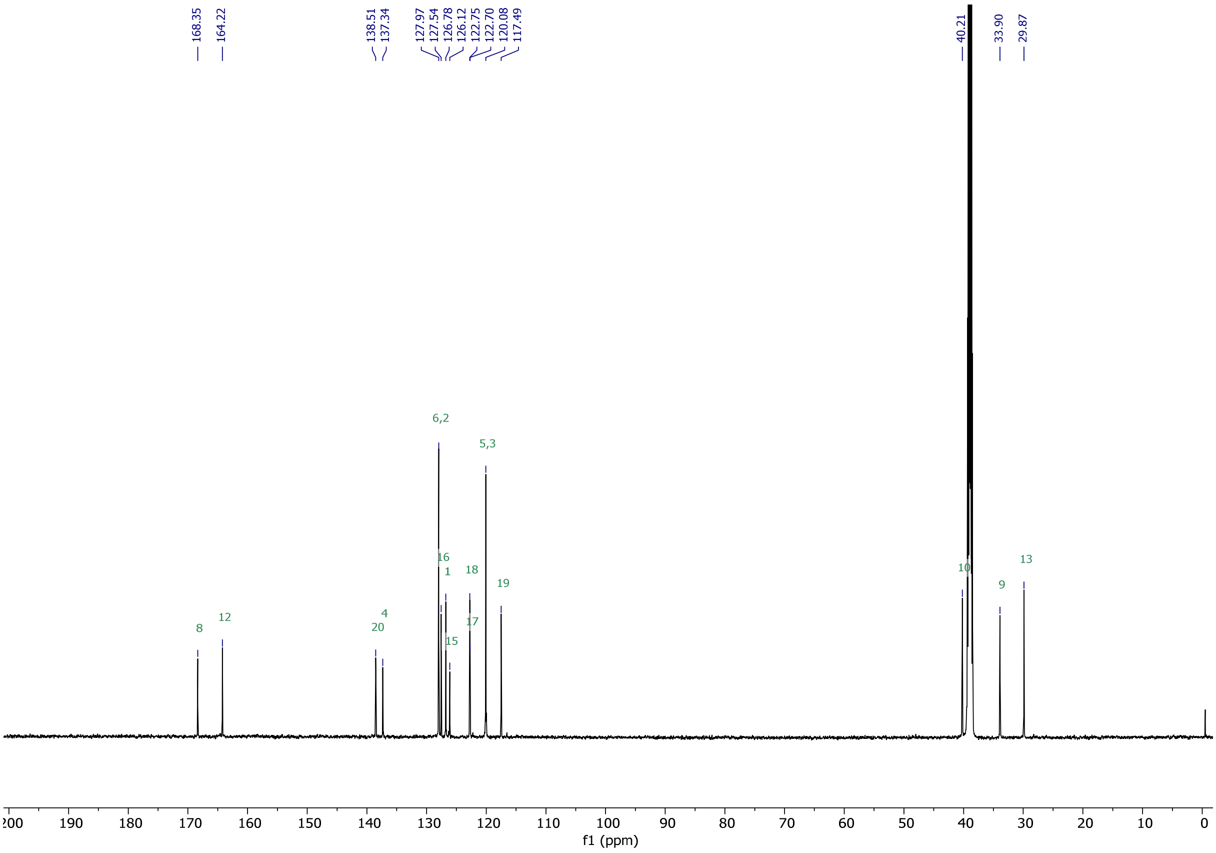

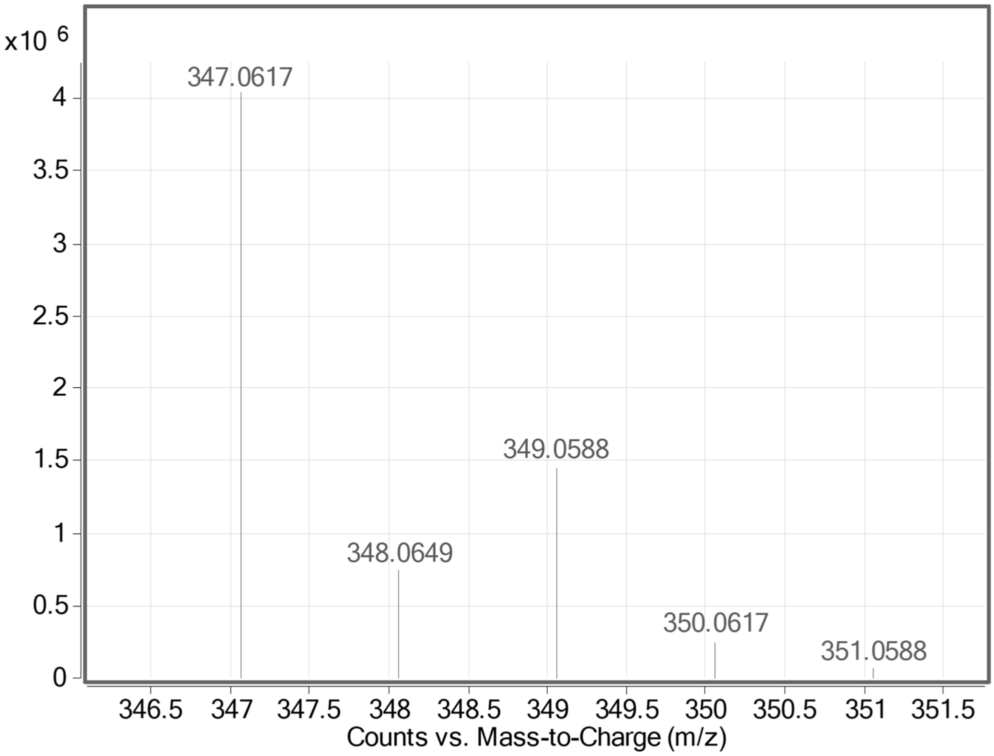
x10 6
4
3.5
3
2.5
2
1.5
1
0.5
0
347.0617
348.0649
349.0588
350.0617
351.0588
346.5 347 347.5 348 348.5 349 349.5 350 350.5 351 351.5
Counts vs. Mass-to-Charge (m/z)

_N_-(2-methoxyphenyl)-3-(3-oxo-2,3-dihydro-4_H_-benzo[_b_][1,4]thiazin-4-yl)propanamide (18)

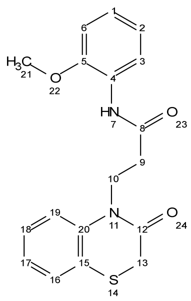

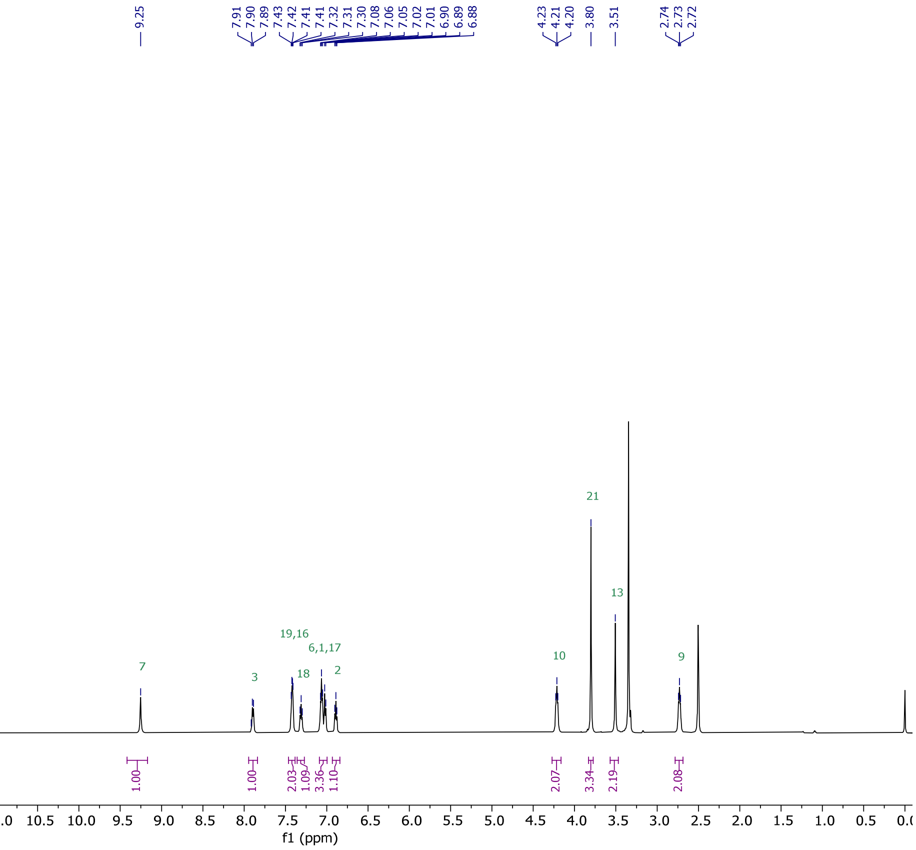

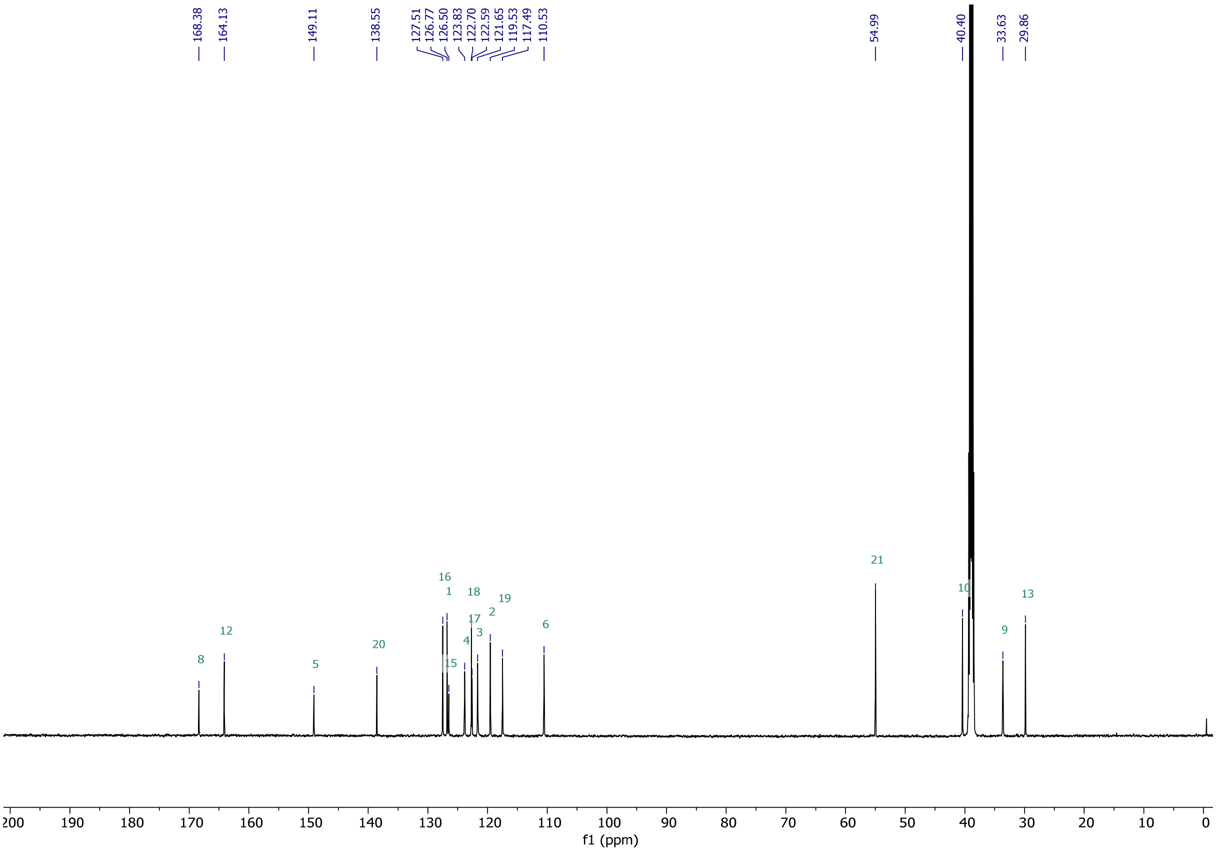

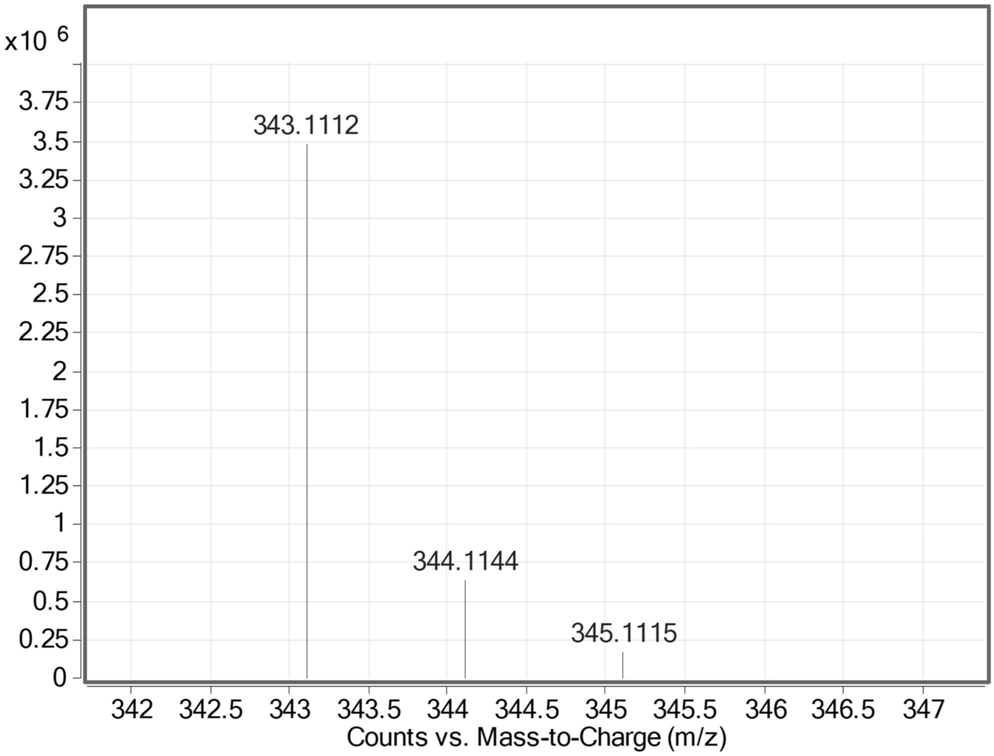

x10 6
3.75
3.5
3.25
3
2.75
2.5
2.25
2
1.75
1.5
1.25
1
0.75
0.5
0.25
0
343.1112
344.1144
345.1115
342
342.5
343
343.5
344
344.5
345
345.5
346
346.5
347
Counts vs. Mass-to-Charge (m/z)

N-(3-methoxyphenyl)-3-(3-oxo-2,3-dihydro-4H-benzo[b][1,4]thiazin-4-yl)propanamide (19)

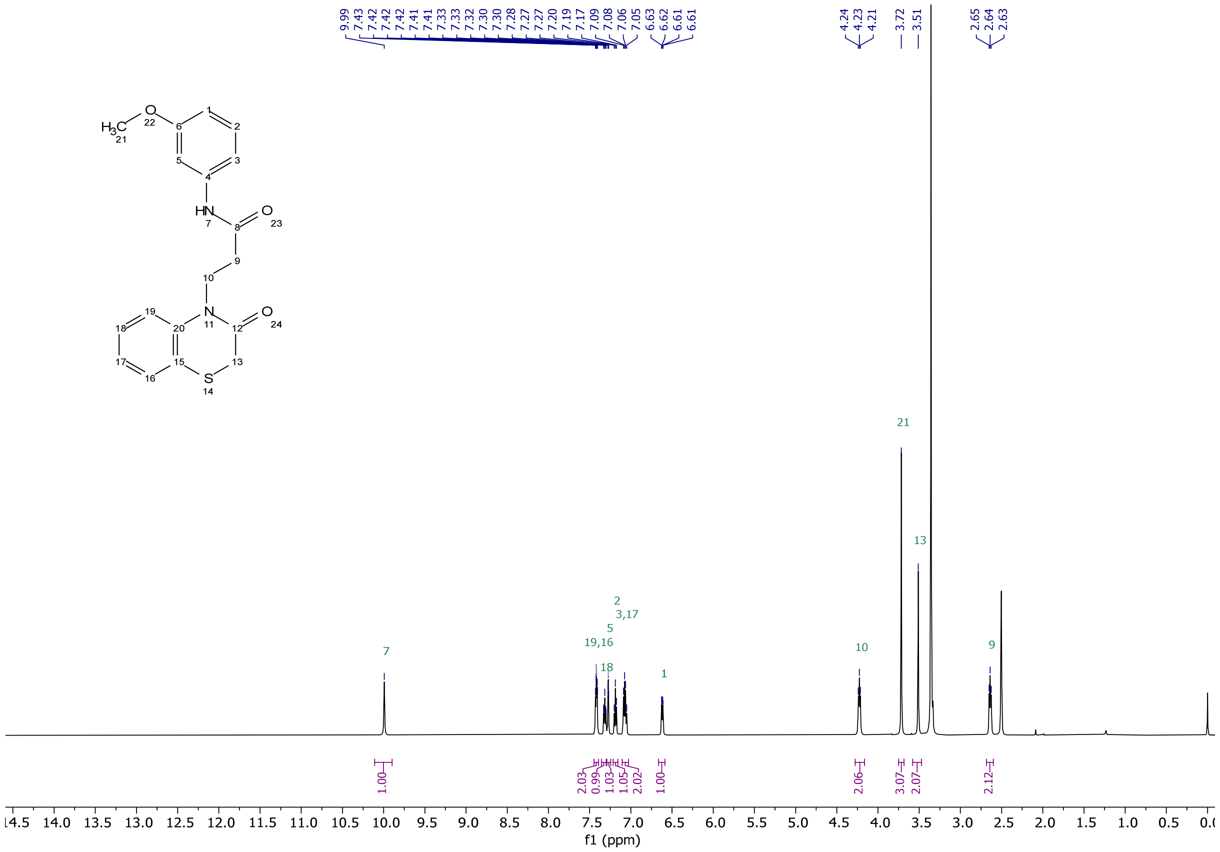

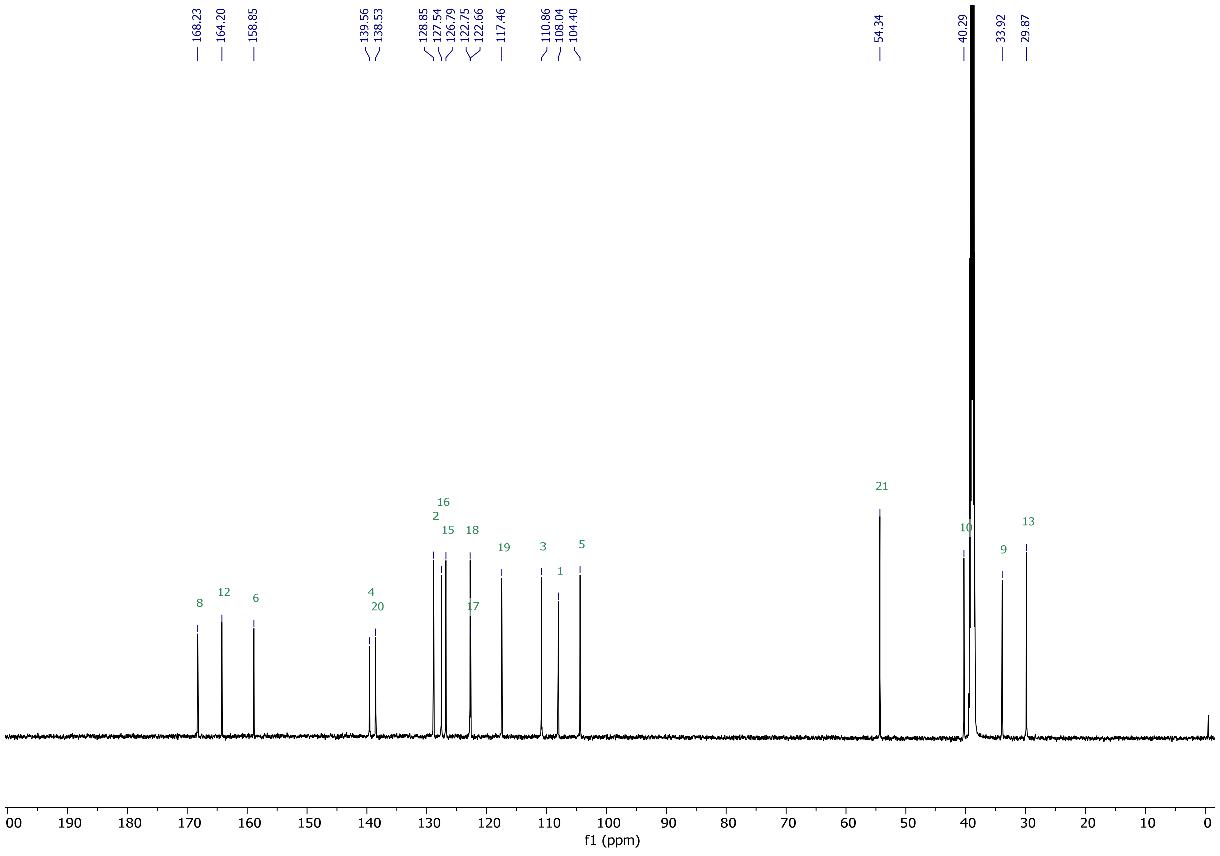

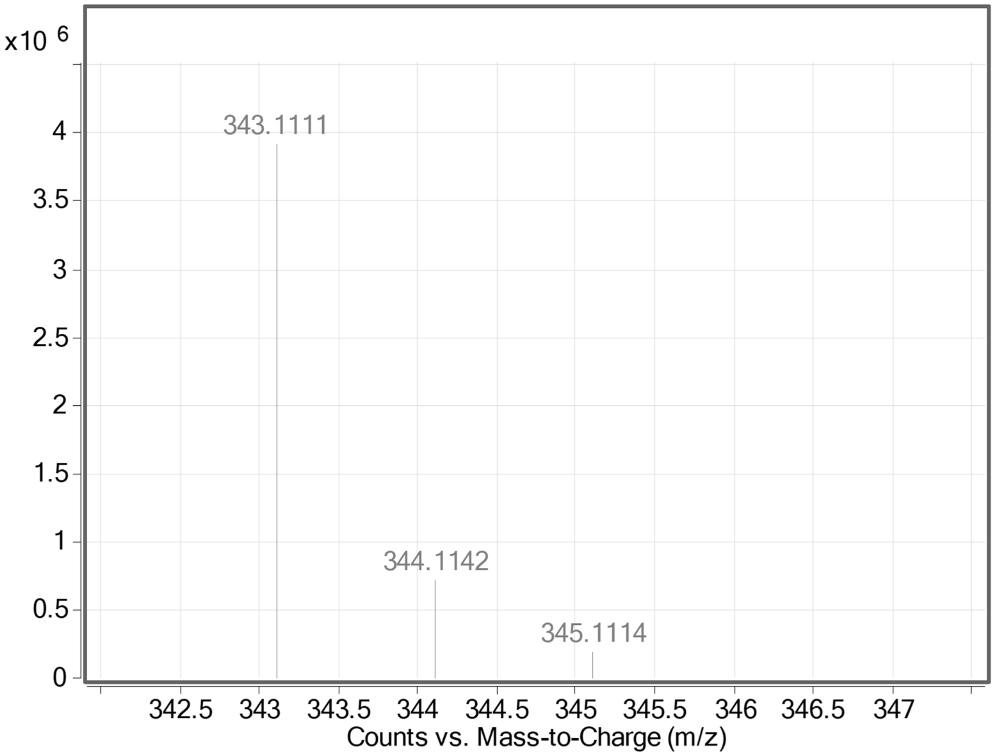
x10 6
343.1111
344.1142
345.1114
0
0.5
1
1.5
2
2.5
3
3.5
4
342.5
343
343.5
344
344.5
345
345.5
346
346.5
347
Counts vs. Mass-to-Charge (m/z)

_N_-(4-methoxyphenyl)-3-(3-oxo-2,3-dihydro-4_H_-benzo[_b_][1,4]thiazin-4-yl)propanamide (20)

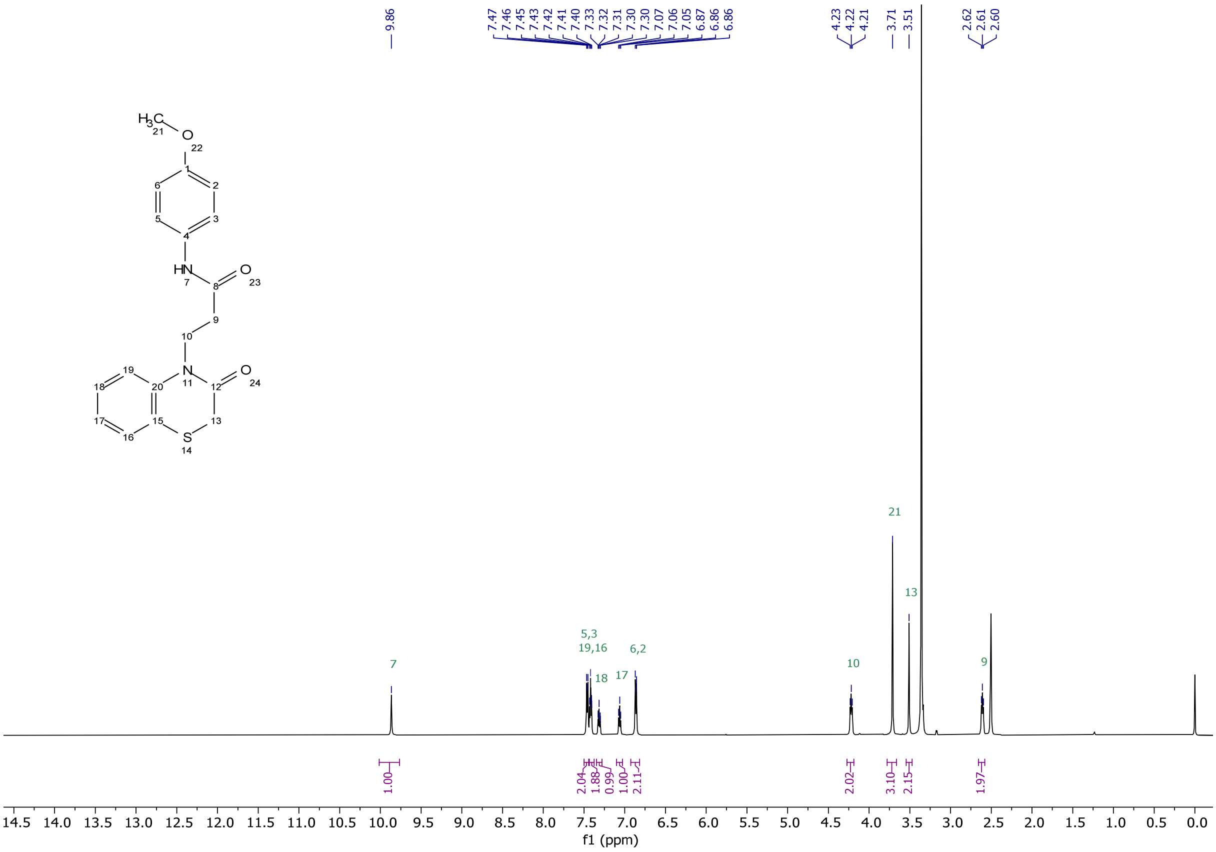

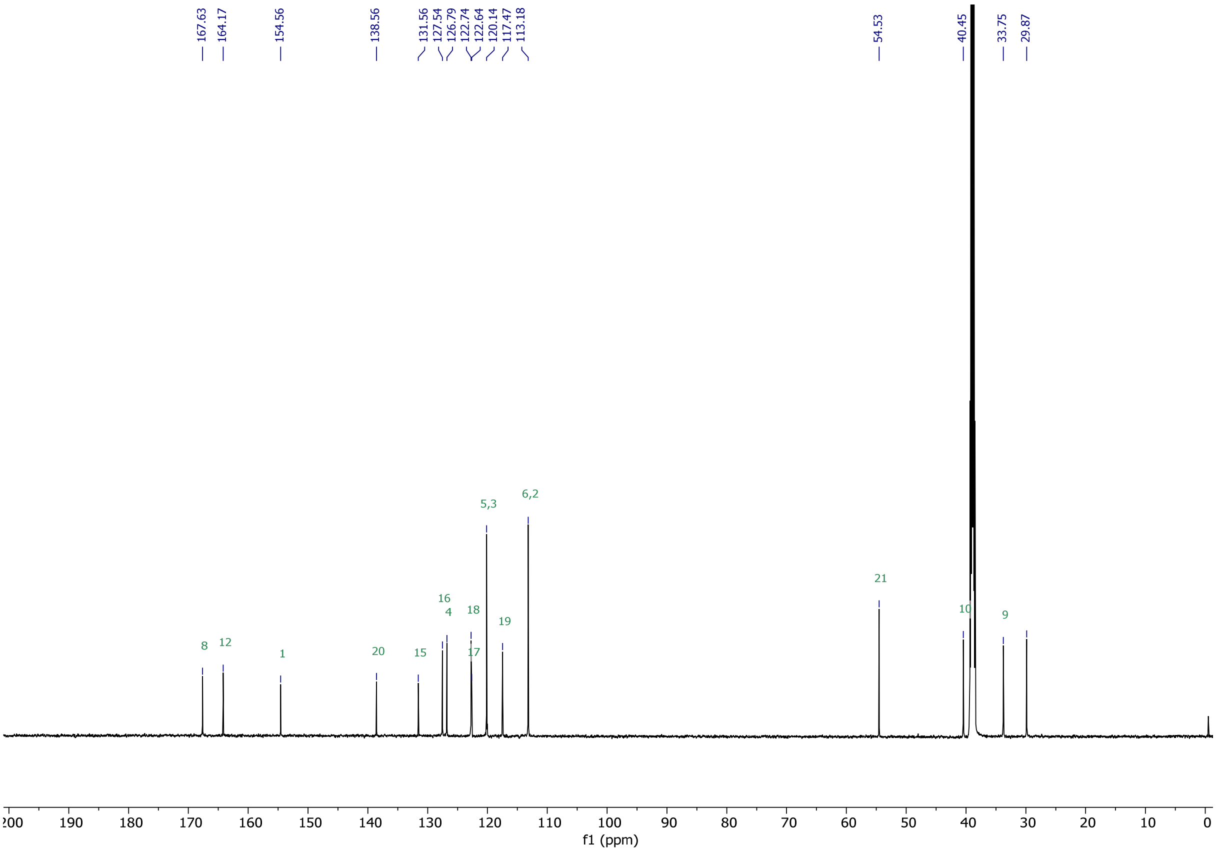

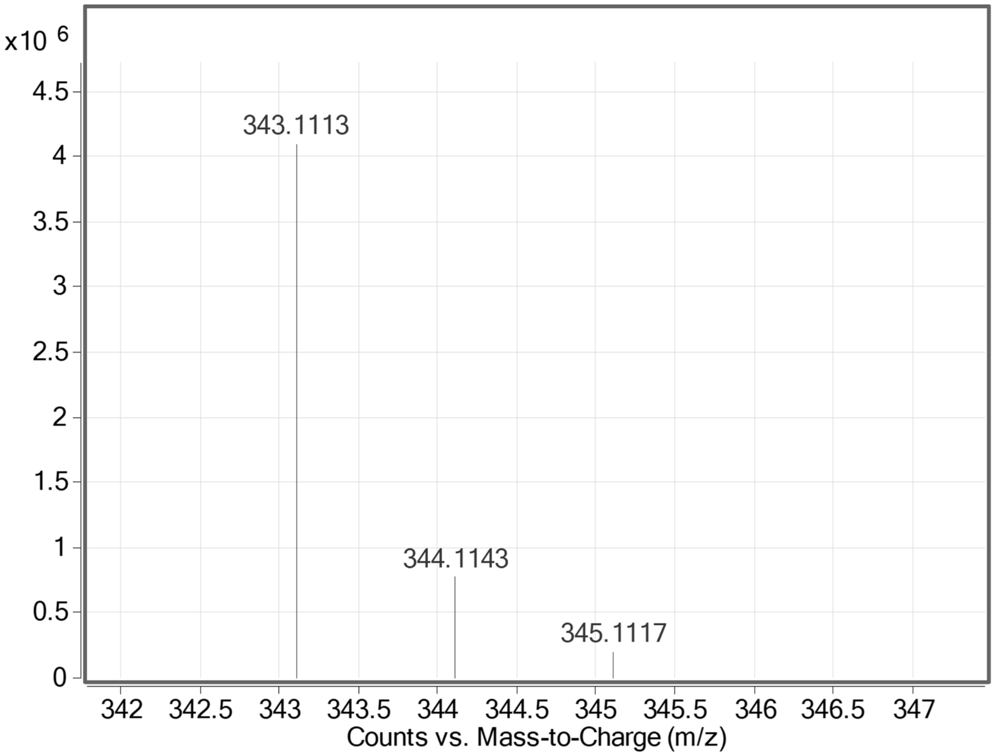
x10 6
4.5
4
3.5
3
2.5
2
1.5
1
0.5
0
343.1113
344.1143
345.1117
342
342.5
343
343.5
344
344.5
345
345.5
346
346.5
347
Counts vs. Mass-to-Charge (m/z)

N-(2-nitrophenyl)-3-(3-oxo-2,3-dihydro-4*H*-benzo[*b*][1,4]thiazin-4-yl)propanamide (21)

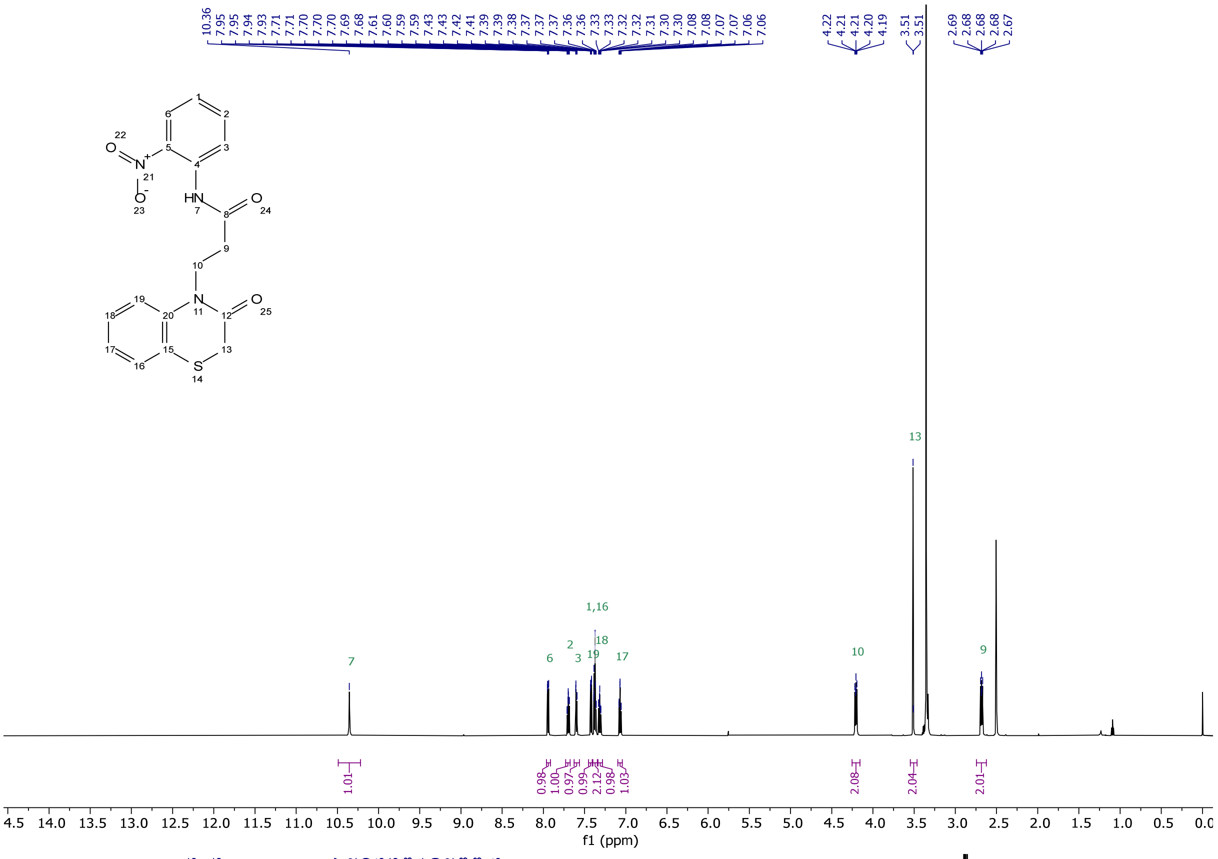

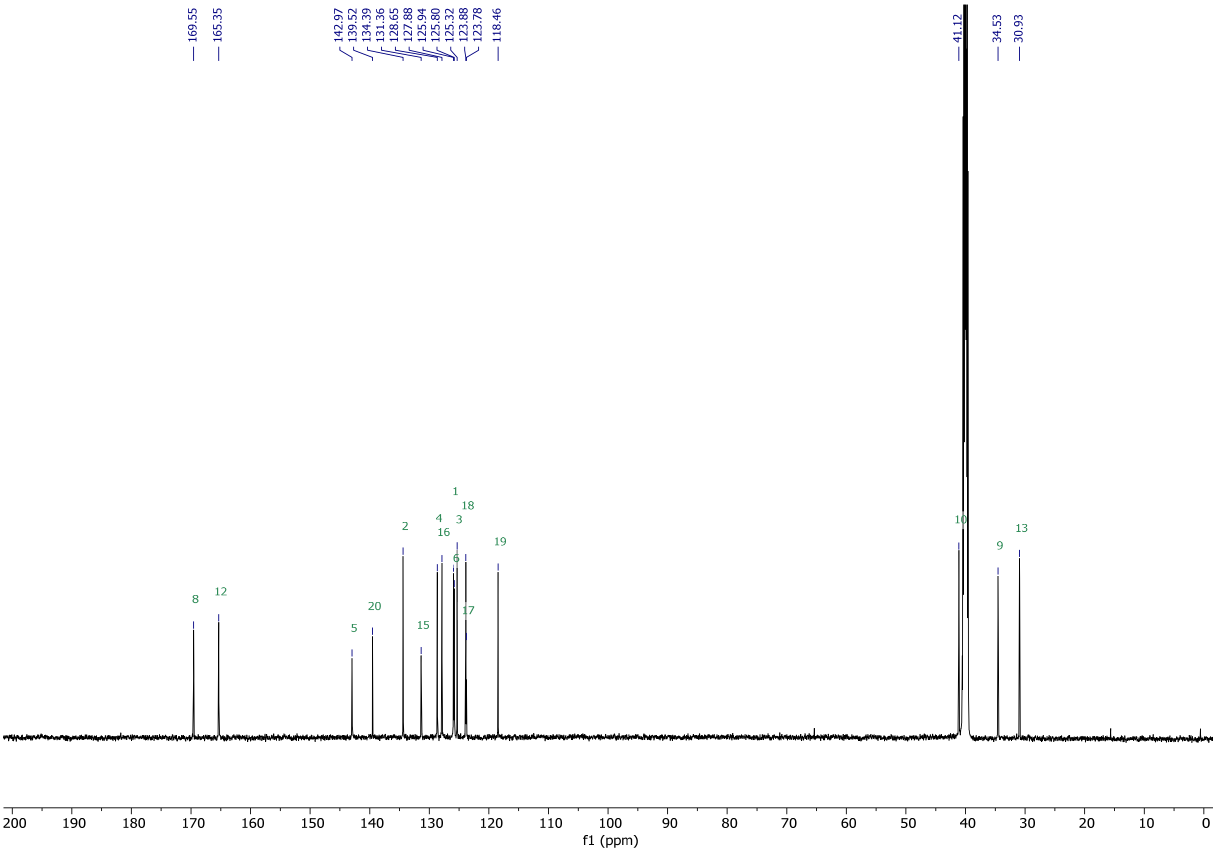

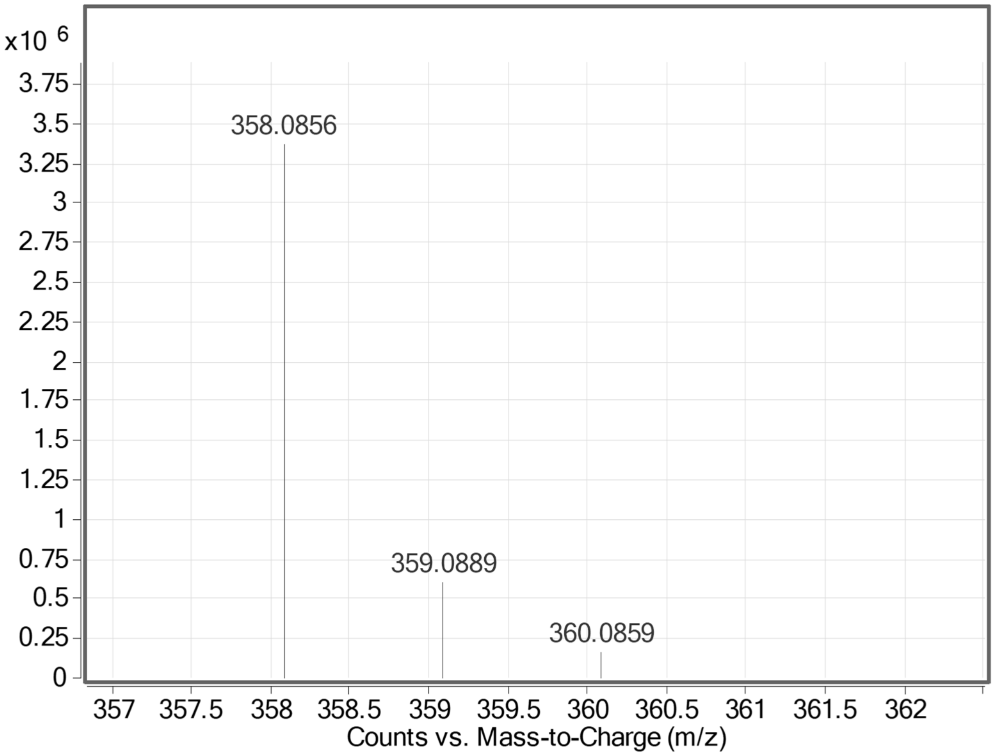
x10 6
3.75
3.5
3.25
3
2.75
2.5
2.25
2
1.75
1.5
1.25
1
0.75
0.5
0.25
0
358.0856
359.0889
360.0859
357
357.5
358
358.5
359
359.5
360
360.5
361
361.5
362
Counts vs. Mass-to-Charge (m/z)

_N_-(3-nitrophenyl)-3-(3-oxo-2,3-dihydro-4_H_-benzo[_b_][1,4]thiazin-4-yl)propanamide (22)

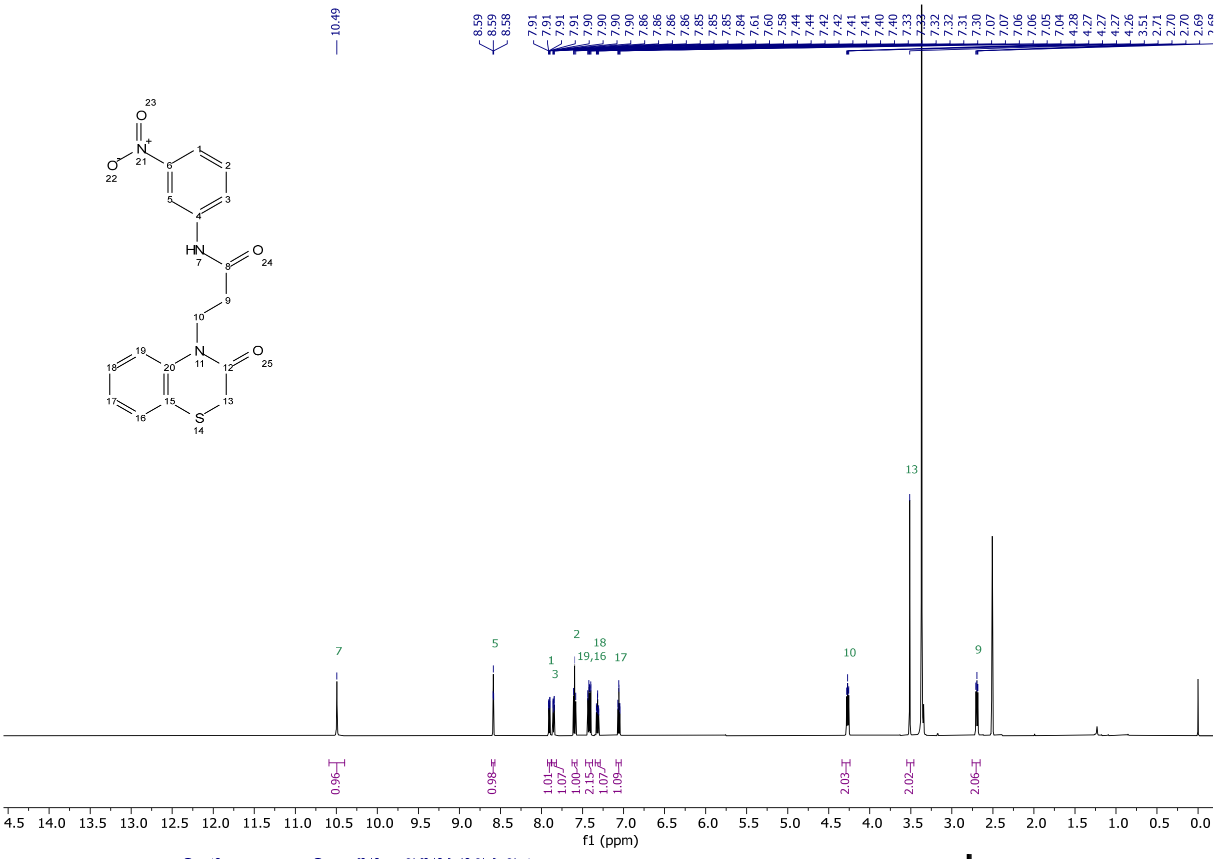

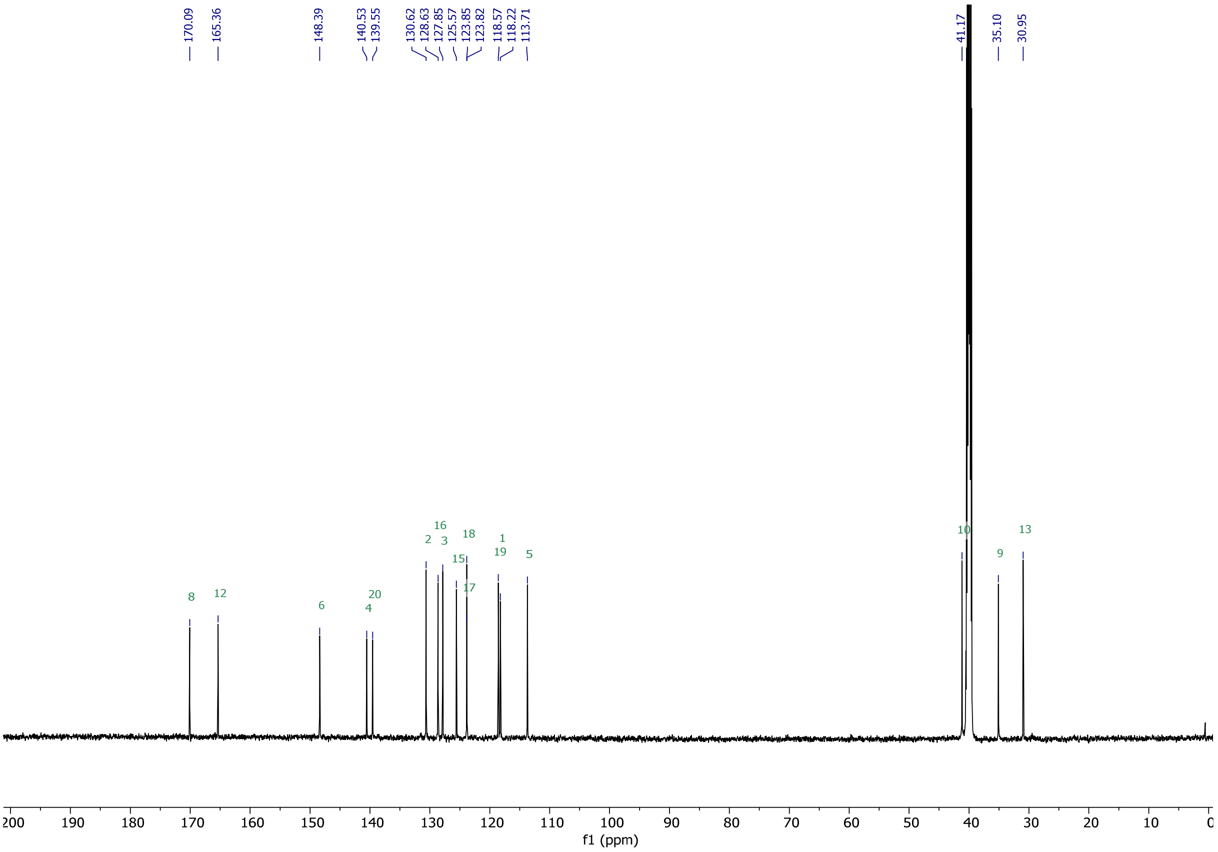

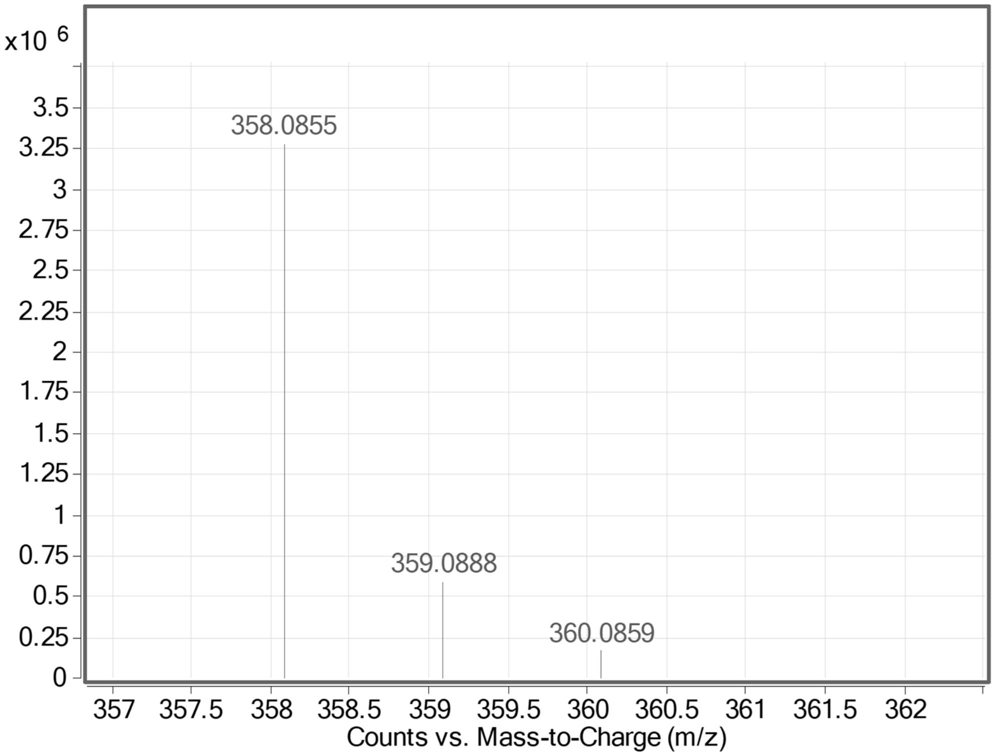

x10 6
3.5
3.25
3
2.75
2.5
2.25
2
1.75
1.5
1.25
1
0.75
0.5
0.25
0
358.0855
359.0888
360.0859
357
357.5
358
358.5
359
359.5
360
360.5
361
361.5
362
Counts vs. Mass-to-Charge (m/z)

_N_-(4-nitrophenyl)-3-(3-oxo-2,3-dihydro-4_H_-benzo[_b_][1,4]thiazin-4-yl)propanamide (23)

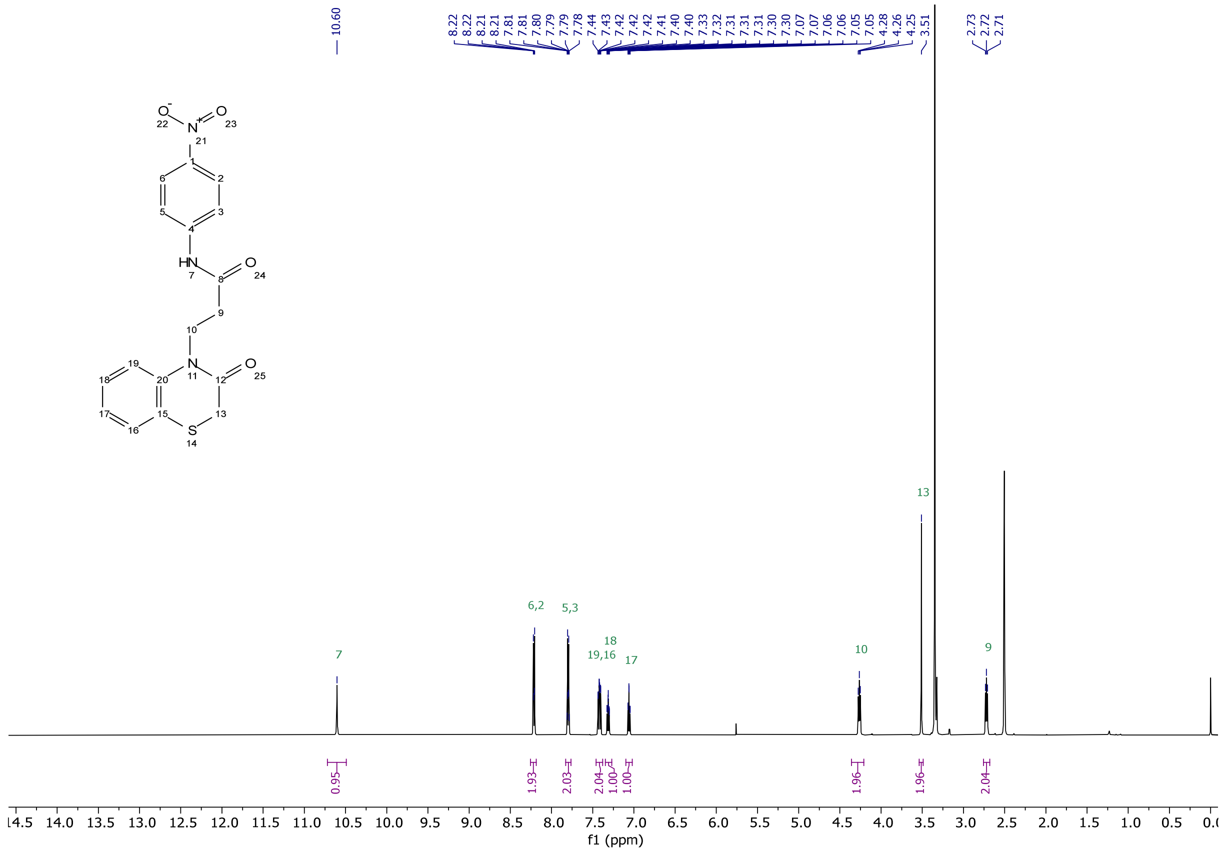

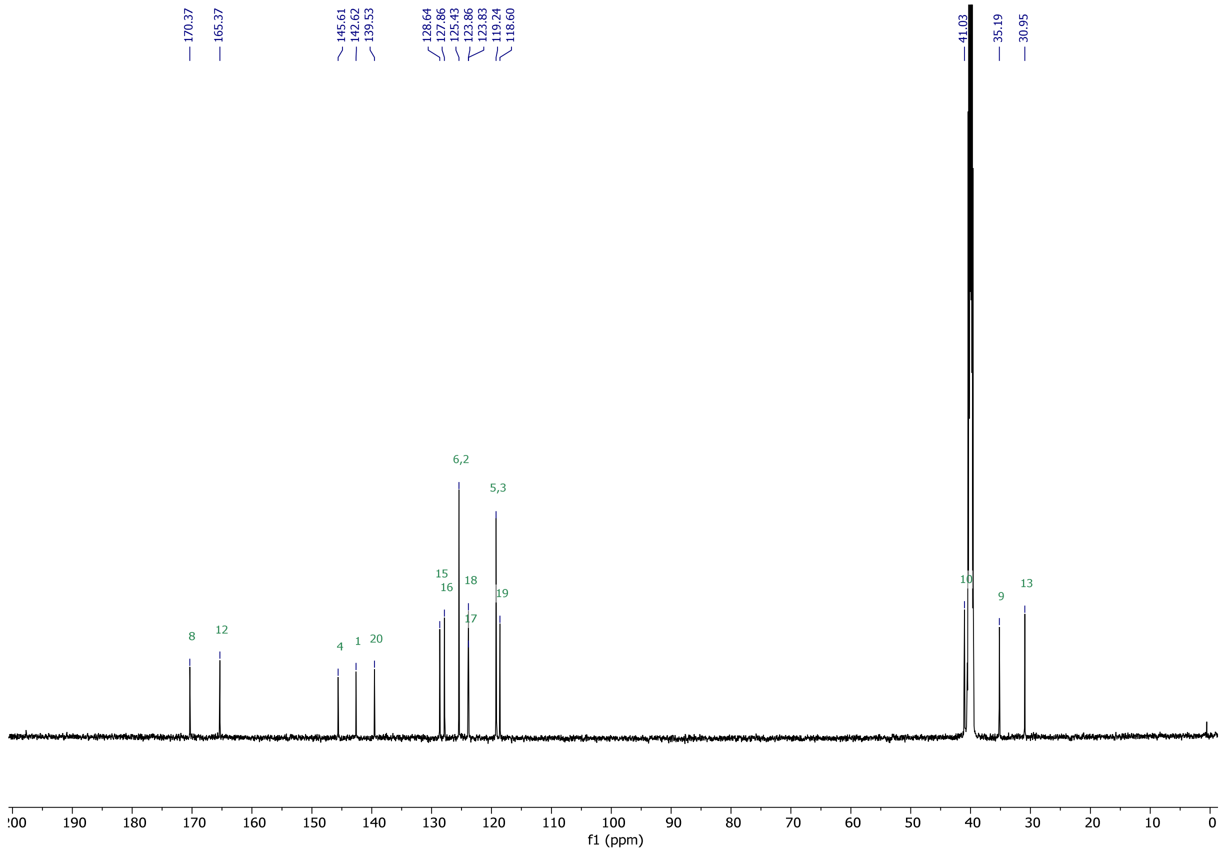

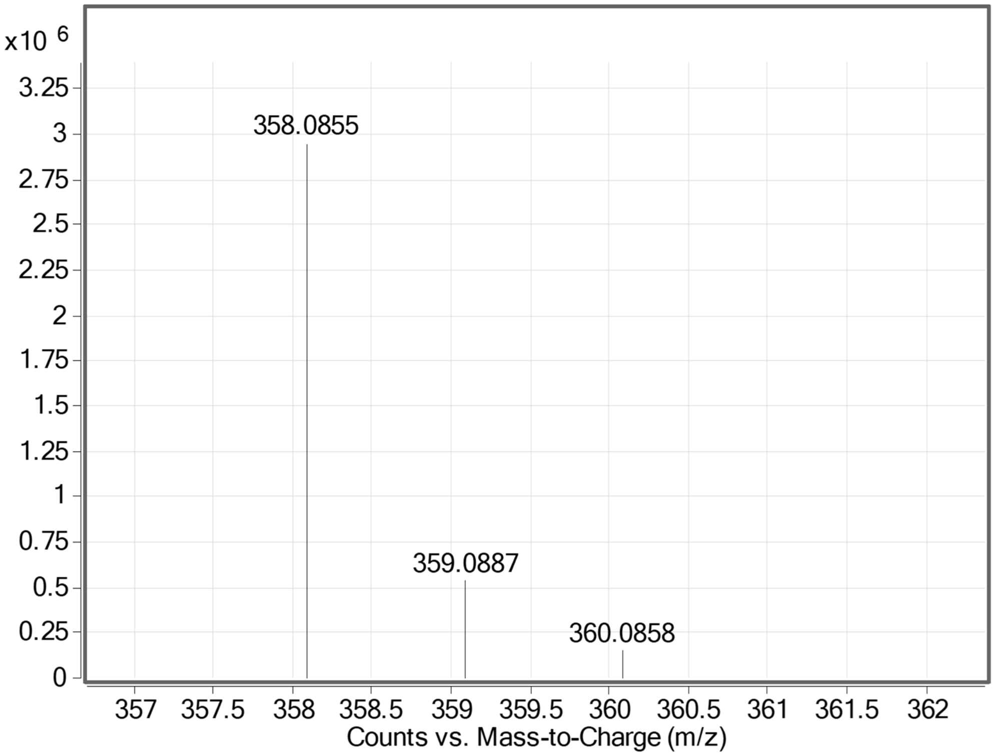
x10 6
3.25
3
2.75
2.5
2.25
2
1.75
1.5
1.25
1
0.75
0.5
0.25
0
358.0855
359.0887
360.0858
357
357.5
358
358.5
359
359.5
360
360.5
361
361.5
362
Counts vs. Mass-to-Charge (m/z)
